# Impact of air pollution exposure on the severity of major depressive disorder: Results from the DeprAir study

**DOI:** 10.1192/j.eurpsy.2024.1767

**Published:** 2024-09-27

**Authors:** E. Borroni, M. Buoli, G. Nosari, A. Ceresa, L. Fedrizzi, L. M. Antonangeli, P. Monti, V. Bollati, A. C. Pesatori, M. Carugno

**Affiliations:** 1 EPIGET Lab, Department of Clinical Sciences and Community Health, University of Milan, Milan, Italy; 2 Department of Pathophysiology and Transplantation, University of Milan, Milan, Italy; 3 Department of Neurosciences and Mental Health, Fondazione IRCCS Ca’ Granda Ospedale Maggiore Policlinico, Milan, Italy; 4 Occupational Health Unit, Fondazione IRCCS Ca’ Granda Ospedale Maggiore Policlinico, Milan, Italy

**Keywords:** air pollution, depression severity, environmental health, major depressive disorder

## Abstract

**Background:**

Major depressive disorder (MDD) is one of the most prevalent medical conditions worldwide. Different factors were found to play a role in its etiology, including environmental ones (e.g., air pollution). The aim of this study was to evaluate the association between air pollution exposure and MDD severity.

**Methods:**

Four hundred sixteen MDD subjects were recruited. Severity of MDD and functioning were evaluated through five rating scales: Montgomery–Asberg Depression Rating Scale (MADRS), Hamilton Depression Rating Scale (HAMD), Clinical Global Impression (CGI), Global Assessment of Functioning (GAF), and Sheehan Disability Scale (SDS). Daily mean estimates of particulate matter with diameter ≤10 (PM10) and 2.5 μm (PM2.5), nitrogen dioxide (NO_2_), and apparent temperature (AT) were estimated based on subjects’ residential addresses. Daily estimates of the 2 weeks preceding recruitment were averaged to obtain cumulative exposure. Multivariate linear and ordinal regression models were applied to assess the associations between air pollutants and MDD severity, overall and stratifying by hypersusceptibility and AT.

**Results:**

Two-thirds of subjects were women and one-third had a family history of depression. Most women had depression with symptoms of anxiety, while men had predominantly melancholic depression. NO_2_ exposure was associated with worsening of MDD severity (HAMD: β = 1.94, 95% confidence interval [CI], [0.41–3.47]; GAF: β = −1.93, 95% CI [−3.89 to 0.02]), especially when temperatures were low or among hypersusceptible subjects. PM exposure showed an association with MDD severity only in these subgroups.

**Conclusions:**

Exposure to air pollution worsens MDD severity, with hypersusceptibility and lower temperatures being exacerbating factors.

## Introduction

Major depressive disorder (MDD) is one of the most common mental disorders affecting 264 million people worldwide: it also represents a leading cause of disability [1] and entails a high risk of suicidal behaviors [2]. Despite its huge social impact, etiopathology of the disorder has not been totally clarified: heritability accounts for approximately 35% [3] but other risk factors have been reported in research studies such as early trauma (sexual, physical, or emotional abuse during childhood) and adverse life events (e.g., lack of a partner, illness or loss of close relatives or friends, financial or social problems, unemployment) [4–6]. In addition, some unchangeable aspects were found to modify the course of the disorder including gender [7], onset in adolescence/advanced age [8, 9], and a history of obstetric complications [10]. Among modifiable risk factors, environmental ones have received increasing interest in the last years [11]. Lack of green spaces [12], poor quality of housing [13], extreme weather events [14], and air pollution [12] have all been associated with risk of MDD.

Ambient or outdoor air pollution is a complex mixture of particulate matter (PM) and gases suspended in the air, including PM of varying sizes, nitrogen oxides, ozone (O_3_), volatile organic compounds (VOCs), and polycyclic aromatic hydrocarbons (PAHs) [15, 16]. This mixture of particles has been found to be the world’s largest single environmental health risk factor, causing about 9 million deaths around the world every year [17, 18]. The overall burden of mortality and morbidity associated with air pollution exposure is very relevant, and includes various diseases, such as asthma and pulmonary illnesses, heart diseases, cancer, as well as mental health disorders [19–22]. In particular, the evidence regarding the potential role of air pollution on MDD has become stronger: with a recent meta-analysis we documented that both short- and long-term exposures to most particulate and gaseous air pollutants have been associated with increased risk of depression, and estimated that ultrafine particles could be annually responsible for 0.64–1.3 million attributable cases of depression in Europe [23]. Even a more recent meta-analysis, specifically focused on cohort studies only, confirmed the long-term effect of particulate and gaseous pollutants on the risk of depression [24]. The first study investigating this potential association dates back to 2007 [25] but the number of investigations on the topic has been steadily increasing since then. Recently, a large cohort study conducted in Rome (Italy) further confirmed the role of most pollutants in increasing the risk of some mental disorders, including depression [26].

Biological mechanisms underlying the air pollution-depression association are not yet fully understood but multiple hypotheses have been formulated. Exposure to atmospheric pollutants could trigger a systemic inflammatory response, fostered by cytokines released by directly exposed tissues [27, 28]. Such pollution-driven responses could also affect the brain, causing neurotoxicity and neuroinflammation [27]. Air pollution exposure could also increase the levels of hippocampal proinflammatory cytokines and affect hippocampal dendrites [29], as well as produce oxidative stress in the brain [30]. The latter can also interplay with innate or cell-mediated immunity, which has been associated with depression onset [31, 32]. Pollutants could also negatively affect microvascular endothelial viability in humans and decrease tight junction protein levels, thus damaging the blood–brain barrier [27]. Finally, air pollution could trigger the activation of the hypothalamic–pituitary–adrenal axis and the release of certain hormones (e.g., cortisol), which characterizes subjects affected by MDD [33].

To further confirm the association between air pollution and MDD and to try to clarify the biological mechanisms underlying this association, we designed the “Depression is in the air” (DeprAir) study. We hereby focus on the very first of the study aims, which is to assess the association between short-/medium-term exposure to PMs and nitrogen dioxide and severity of MDD. Further details on the protocol of the study and its various phases, including biological markers that are being investigated as potentially relevant, can be found in [34].

## Methods

### Study population

Briefly, subjects receiving a diagnosis of MDD were recruited at the Psychiatry Unit of the Policlinico Hospital in Milan (Lombardy), Italy, from September 2020 to December 2022. Study participants were considered eligible if they met the following criteria: (1) being older than 18 years at the time of enrollment; (2) having received a diagnosis of MDD; (3) being resident in Lombardy at the time of the recruitment; and (4) agreeing to sign an informed consent and donate a blood sample. Participants were excluded when they (1) were younger than 18 years old; (2) had a medical condition associated with behavioral disorders (e.g., unbalanced hypothyroidism or stroke); (3) had abused of drugs in the last 4 weeks; (4) had comorbidities related to other psychiatric disorders (except for personality disorders different from borderline personality disorder); (5) had medical conditions which may alter inflammatory markers (e.g., autoimmune diseases); (6) had known ongoing infections; (7) were taking treatments which could influence the biological markers relevant for the study (e.g., corticosteroids or interferons); and (8) were pregnant. All the information related to inclusion and exclusion criteria was verified by a trained psychiatrist when contacting (either by phone or in person) the patients to propose participation in the study. For most subjects, clinical records allowed the physician to double-check self-reported information. If subjects did not meet inclusion criteria or fell within exclusion criteria, the physician did not proceed with administering the two structured questionnaires (see below).

The study has been approved by the Institutional Review Board of the Fondazione IRCCS Ca’ Granda Ospedale Maggiore Policlinico (approval numbers 498_2020bis and 950_2020).

### Collection of personal data

Each study participant was asked to answer two questionnaires administered by a set of trained psychiatrists.

The first questionnaire included information on sociodemographic data (birth date, gender, height, weight, education, occupational status), recent residential history (current complete address, previous complete address if changed in the last year, traffic status in the residential area), smoking history, current health status and medication, physical activity levels and sedentary behavior, type of diet, and drinking habits.

The second questionnaire was aimed at collecting information on MDD, its history, and characteristics, in detail: family psychiatric history, age at onset, duration of untreated illness (in months), total duration of illness (in years), duration of the latest episode (in months), number of depressive episodes and/or hospitalizations, suicide attempts, psychotic symptoms, seasonality of depression, subtype of depression, history of lifetime substance use disorders, antidepressant treatment.

### Diagnostic criteria and rating scales

The Structural Clinical Interview for DSM-5 (Diagnostic and Statistical Manual of Mental Disorders, Fifth Edition) (SCID – Italian version) was used to confirm the diagnosis of MDD [35]. The psychiatrist evaluated the MDD severity of enrolled subjects by administering five rating scales, which are commonly used in clinical practice to assess the severity of affective symptoms:

- Montgomery–Asberg Depression Rating Scale (MADRS): this tool assesses the core symptoms of MDD (e.g., anhedonia, sadness, and agitation). It is composed of 10 items, as follows: apparent sadness, reported sadness, inner tension, reduced sleep, reduced appetite, concentration difficulties, lassitude, inability to feel, pessimistic thoughts, and suicidal thoughts. Each item is scored through a severity scale which ranges from 0 to 6, with higher scores reflecting more severe symptoms. Ratings have been summed up to obtain an overall score ranging from 0 to 60 [36];
- Hamilton Depression Rating Scale (HAMD) 21-item: this tool assesses anxiety and somatization symptoms associated with MDD. It is based on 21 questions about the types of symptoms associated with depression such as anxiety, mood, insomnia, and somatic symptoms. Each symptom is rated on a scale of 0–2, 0–3, or 0–4 with 0 being absent and 2, 3, or 4 being the most severe. To obtain the overall score of severity (from 0 to 67), ratings have been added [37];
- Clinical Global Impression-severity of illness (CGI): with this tool the psychiatrist is asked to evaluate the global severity of illness by answering the following question: “Considering your total clinical experience with this particular population, how mentally ill is the patient at this moment?.” The answer is given based on a seven-point rating scale: 1 = normal, not at all ill; 2 = borderline mentally ill; 3 = mildly ill; 4 = moderately ill; 5 = markedly ill; 6 = severely ill; 7 = among the most extremely ill subjects [38];
- Sheehan Disability Scale (SDS): this scale is used to evaluate the social dysfunction associated with MDD. It consists of a self-reported assessment of functional impairment composed of five items. The first three are global rating scales which assess impairment in work, home, and family responsibilities. Two additional questions measure perceived stress and social support. All the items are scored individually on a 10-point numerical rating scale, except for the “social support” one: this latest is an answer to the question “in the last week, how much have you been supported by friends, family, colleagues, etc., in percent compared to what needed to function properly?,” and can be scored from 0% to 100% [39].
- Global Assessment of Functioning (GAF): this tool is used to evaluate the overall impairment associated with MDD. In particular, it measures how much a person’s symptoms affect his/her day-to-day life on a scale of 0 to 100. The higher the score, the better the patient is able to handle daily activities, suggesting that a lower score indicates a greater social disfunction associated with depression [40]. As such, this is the only scale where a higher score indicates a less severe disorder.

All these tools have a high inter-rater reliability even if administered by health professionals different from psychiatrists [41].

### Exposure assessment

Air pollution exposure was evaluated as exposure to PM with diameter ≤10 (PM10) and 2.5 μm (PM2.5), and nitrogen dioxide (NO_2_). In order to assign the exposure to each pollutant, each patient’s residential address was translated into spatial coordinates using the web tool GPS Visualizer (https://www.gpsvisualizer.com/, last accessed: January 17, 2023) and geocoded using QGIS (QGIS Development Team, 2022. QGIS Geographic Information System. Open-Source Geospatial Foundation Project. https://qgis.org/it/site/, last accessed: January 17, 2023).

Levels were assigned to each patient using daily mean estimates derived from a chemical transport model developed by the regional environmental protection agency of the Lombardy region (ARPA Lombardia) [42, 43]. The model estimated pollutant concentration levels in each cell of a 1 km × 1 km grid overlaid over the entire regional territory: each subject was thus assigned exposure values based on the cell where his/her residential address fell. To analyze short- and medium-term effects of air pollution, different time windows were investigated (i.e., moving averages) by averaging pollutant levels of the day of recruitment with the levels of the day before (lag 0–1) and of each preceding day up to 30 days before (lag 0–30). However, we identified a priori as more relevant for the study outcome lag 0–14, since all the used severity scales investigate symptoms and characteristics which roughly refer to some days to few weeks preceding the evaluation.

As a sensitivity analysis, we also assessed exposure to air pollutants by assigning daily measurements retrieved from the ARPA Lombardia air quality monitoring network to each subject, based on the monitor closest to his/her residential address. Missing values for each pollutant measurement on a specific day and monitor were imputed by computing the average of measurements of that pollutant for the previous and the following 7 days.

We also calculated exposure to apparent temperature (AT), which is a measure of temperature that takes into account humidity and wind speed. All meteorological data were retrieved from the weather monitoring stations of ARPA Lombardia. Daily means of temperature, humidity, and wind speed were then assigned to each subject considering the closest station to his/her residential address. Missing values for each meteorological variable were managed as for air pollution data: out of a total of >35.000 records (1.096 days of recruiting period × 32 weather stations), data were imputed for about 30% of observations. The formula for calculation of AT can be found in [44]. As for air pollutants’ exposure, daily estimates of AT exposure were obtained for different lags.

### Statistical analyses

Descriptive analyses of study population characteristics, variables describing characteristics of MDD, and related severity scales were performed on the entire population and stratifying by gender. When variables followed a continuous normal distribution, data were summarized reporting mean values and standard deviation (SD); for categorical variables, absolute and relative frequencies were reported. Differences by gender were tested using *t* test for unmatched data for continuous normal variables and chi-squared test for categorical variables.

To study the association between different air pollutants exposure and severity of MDD, multivariate linear regression models were used for all the severity scales, except for CGI where a multivariate ordinal regression model was used, due to the ordinal nature of this variable. Models were adjusted for AT (same lag as for air pollution), gender, age, occupation, education, and month and year of recruitment. Adjustment variables were chosen using a DAG-based approach (Supplementary Figure 1). We also considered the potential effect modification of hypersusceptibility status and AT. Hypersusceptibility was defined as the presence of at least one among type II diabetes, current smoking, obesity (body mass index [BMI] ≥ 30), hypertension, and hypercholesterolemia, as all these conditions are characterized by low-grade chronic inflammation [45]. AT was stratified in two categories: ≤25th percentile vs >25th percentile of the variable distribution. To estimate effect modification, the same linear and ordinal regression models previously mentioned were applied, adding the interaction term between the specific pollutant and hypersusceptibility or AT, and extracting stratified estimates from these models.

The results that follow are expressed as regression coefficients or slopes (β) per 10 μg/m^3^ increase in air pollutants concentrations, with corresponding 95% confidence intervals (95% CI). Analyses were performed using Stata (Stata Corp. 2023; Stata Statistical Software: Release 17; College Station, Texas, USA: Stata Corp LLC).

## Results

The main socio-demographic characteristics of the study participants are summarized in Table 1. Two-thirds of the enrolled subjects were women, who were on average older than men (52 vs. 48 years old, *p* = 0.042) and more likely underweight. Most subjects were of normal weight, never smokers, employed, recruited during outpatient visits, and had a high school degree, with no substantial differences between men and women.

**Table 1.** Demographic and lifestyle characteristics of the 416 included subjects

	Mean (SD)/*N* (%)			
Socio-demographic characteristics	Overall	Males	Females	*p* value
Age	50.8 (17.8)	48.4 (17.5)	52.1 (17.8)	**0.042**
Gender				
Females	266 (63.9%)	–	–	
Males	150 (36.1%)	–	–	–
BMI				
Underweight	28 (6.7%)	3 (2.0%)	25 (9.4%)	
Normal weight	228 (54.8%)	85 (56.7%)	143 (53.8%)	
Overweight	101 (24.3%)	39 (26.0%)	62 (23.3%)	
Obese	59 (14.2%)	23 (15.3%)	36 (13.5%)	**0.038**
Education level				
Primary school or less	23 (5.5%)	2 (1.3%)	21 (7.9%)	
Secondary school	78 (18.8%)	30 (20.0%)	48 (18.1%)	
High school	192 (46.2%)	72 (48.0%)	120 (45.1%)	
University	123 (29.6%)	46 (30.7%)	77 (29.0%)	**0.049**
Occupation				
Employed	170 (40.9%)	64 (42.7%)	106 (39.9%)	
Unemployed	102 (24.5%)	38 (25.3%)	64 (24.1%)	
Retired	98 (23.6%)	31 (20.7%)	67 (25.2%)	
Other	46 (11.1%)	17 (11.3%)	29 (10.9%)	0.778
Smoking status				
Never smoker	225 (54.1%)	70 (46.7%)	155 (58.3%)	
Former smoker	49 (11.8%)	21 (14.0%)	28 (10.5%)	
Current smoker	142 (34.1%)	59 (39.3%)	83 (31.2%)	0.073
Second–hand smoking exposure				
Yes	134 (32.2%)	47 (31.3%)	87 (32.7%)	
No	282 (67.8%)	103 (68.7%)	179 (67.3%)	0.773
Residence traffic exposure				
Mild	101 (24.3%)	40 (26.7%)	61 (22.9%)	
Moderate	152 (36.5%)	50 (33.3%)	102 (38.4%)	
Heavy	163 (39.2%)	60 (40.0%)	103 (38.7%)	0.535
Source of recruitment				
Outpatients	158 (38.0%)	55 (36.7%)	103 (38.7%)	
Day–hospital	77 (18.5%)	25 (16.7%)	52 (19.6%)	
Hospitalizations	82 (19.7%)	40 (26.7%)	42 (15.8%)	
Already known outpatients recontacted for the study	99 (23.8%)	30 (20.6%)	69 (25.9%)	0.052

When looking at the main characteristics of MDD (Table 2), our population showed an average age of disorder onset of 39 years. About half of the study subjects had a positive family history of psychiatric disorders, with a higher prevalence among women (51% vs. 34% in men). Women also showed a greater proportion of family history of MDD, while men were more likely hospitalized for MDD (35% vs. 24% in women). Prevalence of psychotic symptoms, suicide attempts, and seasonality of MDD were present in 9%, 18%, and 27% of subjects, respectively, with no differences across genders. The most frequent subtypes of MDD were the melancholic one and the one characterized by prominent symptoms of anxiety, with the first one being more frequent in men (45%) and the second one more prevalent among women (42%). About 20% of the entire population declared a single or multiple lifetime substance use disorder, with alcohol and cannabis representing the more frequent substances among ever abusers. The largest majority of the study population was taking at least one antidepressant treatment at the time of recruitment, with Selective Serotonin Reuptake Inhibitors (SSRIs) or Serotonin and Norepinephrine Reuptake Inhibitors (SNRIs) being the most frequently prescribed.

**Table 2.** Major depressive disorder (MDD) characteristics of the 416 included subjects

	Mean (SD)/*N* (%)			
Major depressive disorder (MDD) characteristics	Overall	Males	Females	*p* value
Age at onset of MDD	39.3 (17.7)	38.6 (17.3)	39.8 (18.0)	0.527
Number of MDD episodes	2.9 (2.8)	2.8 (2.5)	3.0 (3.0)	0.515
Family history of psychiatric disorders				
Yes	187 (45.0%)	51 (34.0%)	136 (51.1%)	
No	229 (55.1%)	99 (66.0%)	130 (48.9%)	**0.001**
Family history of MDD				
Yes	134 (32.2%)	39 (26.0%)	95 (35.7%)	
No	282 (67.8%)	111 (74.0%)	171 (64.3%)	**0.042**
Total duration of untreated MDD in months	20.3 (49.7)	20.4 (46.8)	20.2 (51.3)	0.973
Total duration of MDD in years	10.8 (12.7)	9.5 (10.7)	11.5 (13.7)	0.130
Duration of last MDD episode in months	9.1 (12.9)	9.0 (15.2)	9.2 (11.5)	0.907
Hospitalizations for MDD				
Yes	115 (27.6%)	52 (34.7%)	63 (23.7%)	
No	301 (72.4%)	98 (65.3%)	203 (76.3%)	**0.016**
Among those hospitalized for MDD (115), N of hospitalizations	1.8 (1.5)	1.7 (1.2)	1.9 (1.8)	0.541
Psychotic symptoms				
Yes	36 (8.7%)	14 (9.3%)	22 (8.3%)	
No	380 (91.4%)	136 (90.7%)	244 (91.7%)	0.711
Suicide attempts				
Yes	75 (18.0%)	31 (20.7%)	44 (16.5%)	
No	341 (82.0%)	119 (79.3%)	222 (83.5%)	0.293
Seasonality of MDD				
Yes	113 (27.2%)	36 (24.0%)	77 (29.0%)	
No	303 (72.8%)	114 (76.0%)	189 (71.1%)	0.276
MDD subtype				
Melancholic	159 (38.2%)	67 (44.7%)	92 (34.6%)	
Atypical	60 (14.4%)	20 (13.3%)	40 (15.0%)	
Psychotic	19 (4.6%)	10 (6.7%)	9 (3.4%)	
Strong symptoms of anxiety	146 (35.1%)	35 (23.3%)	111 (41.7%)	
No prevalent type	32 (7.7%)	18 (12.0%)	14 (5.3%)	**0.001**
Lifetime substances abuse				
Single abuse	61 (14.7%)	28 (18.7%)	33 (12.4%)	
Multiple abuse	25 (6.0%)	16 (10.7%)	9 (3.4%)	
No	330 (79.3%)	106 (70.7%)	224 (84.2%)	**0.001**
Type(s) of abuse (among ever abusers (86))				
Alcohol	45 (52.3%)	25 (56.8%)	20 (47.6%)	0.393
Cannabis	37 (43.0%)	21 (47.7%)	16 (38.1%)	0.367
Heroine	8 (9.3%)	6 (13.6%)	2 (4.8%)	0.157
Cocaine	14 (16.3%)	9 (20.5%)	5 (11.9%)	0.283
LSD	5 (5.8%)	3 (6.8%)	2 (4.8%)	0.684
Amphetamine	4 (4.7%)	3 (6.8%)	1 (2.4%)	0.329
Methyl enedioxy methamphetamine (MDMA)	1 (1.2%)	0 (0.0%)	1 (2.4%)	0.303
Drugs	18 (20.9%)	5 (11.4%)	13 (31.0%)	**0.026**
Any antidepressant treatment				
Yes	365 (87.7%)	133 (88.7%)	232 (87.2%)	
No	51 (12.3%)	17 (11.3%)	34 (12.8%)	0.665
Treatment type (among 280 subjects taking treatment)				
Selective serotonin reuptake inhibitors (SSRIs)	228 (62.5%)	86 (64.7%)	142 (61.2%)	
Serotonin and norepinephrine reuptake inhibitors (SNRIs)	59 (16.2%)	19 (14.3%)	40 (17.2%)	
Tricyclics	32 (8.8%)	9 (6.8%)	23 (9.9%)	
Bupropion	2 (0.6%)	1 (0.8%)	1 (0.4%)	
Mirtazapine	14 (3.8%)	7 (5.3%)	7 (3.0%)	
Vortioxetine	11 (3.0%)	5 (3.8%)	6 (2.6%)	
Trazodone	12 (3.3%)	1 (0.8%)	11 (4.7%)	
Others	7 (1.9%)	5 (3.8%)	2 (0.9%)	0.138

Based on both the MADRS and the HAMD rating scale scores, about 70% of the enrolled subjects were considered to have a moderate-to-severe depression. According to the GAF scale, an average score of about 59 was observed, suggesting that, on average, subjects had moderate difficulties in social functioning. When using the CGI, the enrolling psychiatrists judged about 60% of recruited subjects as being moderately to mildly ill. Finally, the subdomains of the SDS investigating the social, domestic, and family impairment due to MDD as well as the perceived stress deriving from the disorder returned average scores above 6. On average, the enrolled subjects declared they obtained a social support that was about 58% of what needed to function properly. All severity scales showed similar scores in the two genders, even if the “perceived social support” domain of the SDS was higher among women (Table 3).

**Table 3.** Scales of major depressive disorder (MDD) severity in the 416 included subjects

	Mean (SD)/*N* (%)			
Major depressive disorder (MDD) severity	Overall	Males	Females	*p* value
Montgomery–Asberg Depression Rating Scale (MADRS; 0–60)	28.3 (12.6)	28.7 (12.7)	28.1 (12.5)	0.632
0–6 (no depression)	17 (4.1%)	6 (4.0%)	11 (4.1%)	
7–19 (mild depression)	84 (20.2%)	29 (19.3%)	55 (20.7%)	
20–34 (moderate depression)	182 (43.8%)	62 (41.3%)	120 (45.1%)	
≥35 (severe depression)	133 (32.0%)	53 (35.3%)	80 (30.1%)	0.745
Hamilton Depression Rating Scale (HAMD; 0–67)	23.9 (12.3)	23.8 (12.3)	23.9 (12.4)	0.929
0–7 (no depression)	27 (6.5%)	11 (7.3%)	16 (6.0%)	
8–16 (mild depression)	93 (22.4%)	32 (21.3%)	61 (22.9%)	
17–23 (moderate depression)	108 (26.0%)	43 (28.7%)	65 (24.4%)	
≥24 (severe depression)	188 (45.2%)	64 (42.7%)	124 (46.6%)	0.715
Global Assessment of Functioning (GAF; 100–0)	58.6 (15.1)	57.2 (16.0)	59.3 (14.6)	0.164
Clinical Global Impression (CGI; 0–7)				
Normal, not at all ill	18 (4.3%)	5 (3.3%)	13 (4.9%)	
Borderline mentally ill	38 (9.1%)	9 (6.0%)	29 (10.9%)	
Mildly ill	92 (22.1%)	32 (21.3%)	60 (22.6%)	
Moderately ill	129 (31.0%)	48 (32.0%)	81 (30.5%)	
Markedly ill	75 (18.0%)	27 (18.0%)	48 (18.1%)	
Severely ill	52 (12.5%)	22 (14.7%)	30 (11.3%)	
Among the most extremely ill patients	12 (2.9%)	7 (4.7%)	5 (1.9%)	0.353
Sheehan Disability Scale (SDS)				
Impairment at work (0–10)	7.1 (2.8)	7.2 (2.7)	7.0 (2.7)	0.513
Impairment in home relationships (0–10)	6.8 (2.6)	7.0 (2.7)	6.7 (2.5)	0.195
Impairment in family responsibilities (0–10)	6.6 (2.8)	6.7 (2.8)	6.5 (2.8)	0.570
Perceived stress (0–10)	6.3 (2.8)	6.5 (3.0)	6.2 (2.7)	0.453
Perceived social support (0–100)	57.5 (27.2)	53.6 (28.6)	59.7 (26.1)	0.028

**Figure 1.**
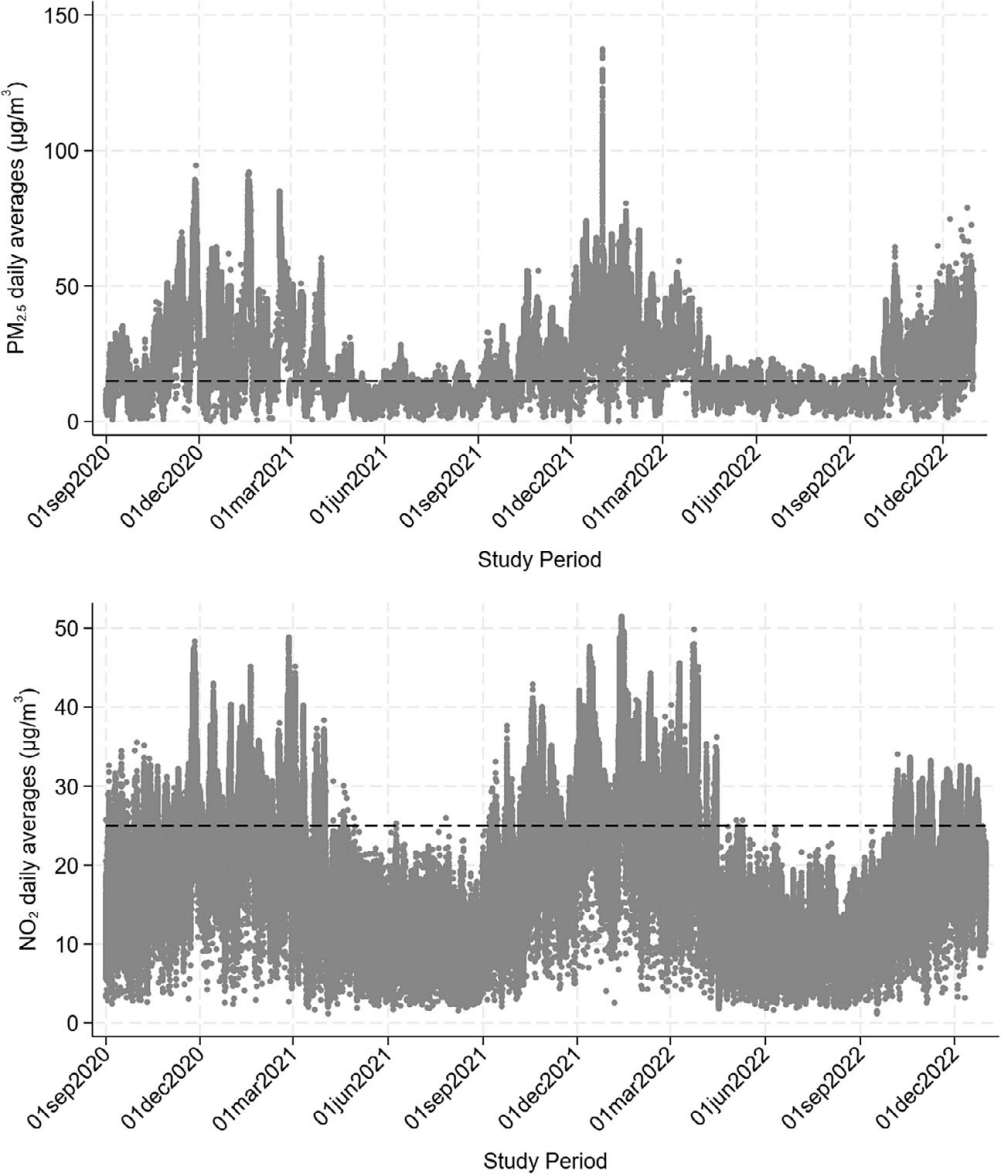
Trend of PM2.5 (upper box) and NO_2_ (lower box) levels in the period of recruitment estimated by the FARM model within the grid cells where the residential addresses of the study population fell. The dashed line corresponds to the World Health Organization 2021 Air Quality Guidelines for the pollutants daily average (i.e., 15 and 25 μg/m^3^, respectively).

The trends of concentration levels of the three pollutants of interest during the period of recruitment estimated in the grid cells where residential addresses of the study subjects fell are depicted in Figure 1 and Supplementary Figure 2: all three pollutants show a clear seasonal pattern, with higher values in the cold season and lower values in the warm season. The air quality guidelines (AQG) recommended by the World Health Organization as daily average [46] were very often exceeded, in detail: 55% of observations were above the 15 μg/m^3^ AQG for PM2.5, 22% were above the 45 μg/m^3^ AQG for PM10, and 69% were above the 25 μg/m^3^ AQG for NO_2_.

When we evaluated the association with air pollution exposure in the 2 weeks preceding recruitment, no effects of PM2.5 (Table 4 and Supplementary Figures 3–7) or PM10 (Supplementary Table 1) were observed on MDD severity in any of the scales, while a 10 μg/m^3^ increase in NO_2_ concentrations (Table 4 and Supplementary Figures 8–12) was associated with an increasing score of 1.94 (95% CI, 0.41–3.47) for the HAMD scale. Even for the GAF scale (where lower scores indicate more social impairment) we observed a worsening in social dysfunction associated with MDD for higher NO_2_ exposures (β = −1.93, 95% CI, −3.89 to 0.02). On the contrary, increasing NO_2_ values were associated with a decrease in the score of the “perceived social support” domain of the SDS scale (β = −3.77, 95% CI, −7.35 to −0.19).

**Table 4.** Estimates (β) with corresponding 95% confidence intervals (95% CI) and *p*-values of the association between air pollutant exposure (10 μg/m^3^ increase) and major depressive disorder severity rating scales

	β (95% CI) *p* value	
MDD rating scale	PM2.5	NO_2_
MADRS	0.12 (–1.94 to 2.19) *p* = 0.906	0.75 (–0.83 to 2.32) *p* = 0.353
HAMD	0.61 (–1.00 to 2.62) *p* = 0.555	**1.94 (0.41; 3.47)** *p* = **0.013**
GAF	–1.73 (–4.29 to 0.84) *p* = 0.187	**–1.93 (–3.89 to 0.02) *p =* 0.053**
CGI	0.05 (–0.27 to 0.37) *p =* 0.746	0.17 (–0.07 to 0.40) *p* = 0.164
SDS		
Impairment at work	0.25 (–0.32 to 0.81) *p* = 0.391	0.19 (–0.22 to 0.61) *p* = 0.361
Impairment in home relationships	0.12 (–0.32 to 0.57) *p* = 0.588	0.21 (–0.13 to 0.55) *p* = 0.224
Impairment in family responsibilities	0.06 (–0.42 to 0.54) *p* = 0.800	0.33 (–0.03 to 0.70) *p* = 0.074
Perceived stress	0.05 (–0.45 to 0.54) *p* = 0.854	0.17 (–0.20 to 0.55) *p* = 0.368
Perceived social support	–3.33 (–8.02 to 1.36) *p* = 0.164	**–3.77 (–7.35 to –0.19) *p =* 0.039**

Abbreviations: MDD, major depressive disorder; MADRS, Montgomery–Asberg Depression Rating Scale; HAMD, Hamilton Depression Rating Scale; GAF, Global Assessment of Functioning; CGI, Clinical Global Impression; SDS, Sheehan Disability Scale; PM2.5, particulate matter with diameter ≤2.5; NO_2_, nitrogen dioxide; β, beta estimate; 95% CI, confidence interval at 95% level.

When using air quality monitoring data as exposure variables, results were substantially confirmed, with no effects seen for PM2.5 and a positive association observed for NO_2_, even if the latter findings were stronger and significant for a larger number of scales if compared to the main analysis using dispersion models to assign exposures (Supplementary Table 2).

PM2.5 exposure showed to be negatively associated with MDD among hypersusceptible subjects (Table 5 and Supplementary Figures 13 − 17), with significant interactions detected for the MADRS (*p* = 0.006), HAMD (*p* = 0.041), and CGI (*p* = 0.014) scales and for the sub-domains “impairment of home relationships” (*p* = 0.015), “impairment in family responsibilities” (*p* = 0.013), and “perceived stress” (*p* = 0.006) of the SDS scale. Results for PM10 were similar (Supplementary Table 3). Hypersusceptibility status was found to formally be an effect modifier of the association between NO_2_ exposure and MDD severity only for the CGI scale (*p* = 0.039) and for the sub-domain “perceived stress” (*p* = 0.046) of the SDS scale (Table 6 and Supplementary Figures 18 − 22); nonetheless, a pattern similar to particulate pollutants was observed, with stronger (and often significant) associations among hypersusceptible subjects for most scales.

**Table 5.** Stratified estimates (β) by hypersusceptibility status (defined as presence of at least one of the following: obesity, hypercholesterolemia, hypertension, type II diabetes, current smoking), with corresponding 95% confidence intervals (95% CI), *p*-values, and interaction *p*-values of the association between PM2.5 exposure (10 μg/m^3^ increase) and major depressive disorder severity rating scales

	β (95% CI) *p* value		
MDD rating scale	Hypersusceptible subjects	Not hypersusceptible subjects	Interaction *p* value
MADRS	1.32 (–0.90 to 3.54) *p* = 0.242	–1.94 (–4.46 to 0.57) *p* = 0.130	**0.006**
HAMD	1.48 (–0.69 to 3.66) *p* = 0.181	–0.89 (–3.36 to 1.58) *p* = 0.480	**0.041**
GAF	–2.71 (–5.49 to 0.06) *p* = 0.055	–0.04 (–3.19 to 3.11) *p* = 0.979	0.071
CGI	0.21 (–0.13 to 0.56) *p* = 0.224	–0.26 (–0.66 to 0.15) *p* = 0.215	**0.014**
SDS			
Impairment at work	0.40 (–0.22 to 1.02) *p* = 0.202	0.03 (–0.64 to 0.70) *p* = 0.936	0.230
Impairment in home relationships	0.35 (–0.13 to 0.83) *p* = 0.149	–0.27 (–0.81 to 0.28) *p* = 0.336	**0.015**
Impairment in family responsibilities	0.31 (–0.20 to 0.83) *p* = 0.229	–0.36 (–0.94 to 0.22) *p* = 0.222	**0.013**
Perceived stress	0.33 (–0.20 to 0.87) *p* = 0.217	–0.45 (–1.06 to 0.15) *p* = 0.140	**0.006**
Perceived social support	–4.55 (–9.63 to 0.53) *p* = 0.079	–1.18 (–6.94 to 4.58) *p* = 0.687	0.213

Abbreviations: MDD, major depressive disorder; MADRS, Montgomery–Asberg Depression Rating Scale; HAMD, Hamilton Depression Rating Scale; GAF, Global Assessment of Functioning; CGI, Clinical Global Impression; SDS, Sheehan Disability Scale; β, beta estimate; 95% CI, confidence interval at 95% level.

**Table 6.** Stratified estimates (β) by hypersusceptibility status (defined as presence of at least one of the following: obesity, hypercholesterolemia, hypertension, type II diabetes, current smoking), with corresponding 95% confidence intervals (95% CI), *p*-values, and interaction *p*-values of the association between NO_2_ exposure (10 μg/m^3^ increase) and MDD severity rating scales

	β (95% CI) *p* value		
MDD rating scale	Hypersusceptible subjects	Not hypersusceptible subjects	Interaction *p* value
MADRS	1.39 (–0.32 to 3.10) *p* = 0.111	–0.57 (–2.64 to 1.50) *p* = 0.589	0.056
HAMD	**2.29 (0.62–3.95) *p* = 0.007**	1.28 (–0.73 to 3.30) *p* = 0.212	0.313
GAF	**–2.61 (–4.73 to –0.48) *p* = 0.017**	–0.62 (–3.20 to 1.95) *p* = 0.635	0.121
CGI	**0.27 (0.02–0.53) *p* = 0.035**	–0.06 (–0.38 to 0.26) *p* = 0.704	**0.039**
SDS			
Impairment at work	0.27 (–0.19 to 0.73) *p* = 0.241	0.07 (–0.45 to 0.60) *p* = 0.788	0.441
Impairment in home relationships	0.33 (–0.04 to 0.70) *p* = 0.077	–0.02 (–0.47 to 0.42) *p* = 0.917	0.108
Impairment in family responsibilities	**0.46 (0.06–0.85) *p* = 0.024**	0.10 (–0.37 to 0.58) *p* = 0.671	0.136
Perceived stress	0.33 (–0.08 to 0.74) *p* = 0.110	–0.16 (–0.65 to 0.34) *p* = 0.533	**0.046**
Perceived social support	**–4.97 (–8.85 to –1.08) *p* = 0.012**	–1.27 (–5.97 to 3.43) *p* = 0.595	0.113

Abbreviations: MDD, major depressive disorder; MADRS, Montgomery–Asberg Depression Rating Scale; HAMD, Hamilton Depression Rating Scale; GAF, Global Assessment of Functioning; CGI, Clinical Global Impression; SDS, Sheehan Disability Scale; β, beta estimate; 95% CI, confidence interval at 95% level.

We also explored the role of AT as effect modifier. When AT levels were below the first quartile of exposure, strong positive associations were found between PM2.5 exposure (Table 7 and Supplementary Figures 23–27) and severity scores of MDD and related social dysfunction, measured by MADRS (β = 3.64, 95% CI, 0.33–6.95), HAMD (β = 3.86, 95% CI, 0.61–7.11), and GAF (β = –4.71, 95% CI, −8.84 to −0.58). On the contrary, when AT levels were higher than the first quartile of exposure, associations followed the opposite direction, and the presence of interaction was confirmed across most scales. A similar pattern of results was observed for PM10 exposure (Supplementary Table 4). When NO_2_ exposure was considered, stronger estimates were again observed for lower temperatures even if a formal statistical interaction was present only for HAMD (Table 8 and Supplementary Figures 28–32).

**Table 7.** Stratified estimates (β) by apparent temperature (AT), with corresponding 95% confidence intervals (95% CI), *p*-values, and interaction *p*-values of the association between PM2.5 exposure (10 μg/m^3^ increase) and major depressive disorder severity rating scales

	β (95% CI) *p* value		
MDD rating scale	AT ≤ first quartile (5.86 °C)	AT > first quartile (5.86 °C)	Interaction *p* value
MADRS	**3.64 (0.33–6.95) *p* = 0.031**	–2.19 (–4.79 to 0.42) *p* = 0.100	**0.007**
HAMD	**3.86 (0.61–7.11) *p* = 0.020**	–1.60 (–4.15 to 0.95) *p* = 0.219	**0.009**
GAF	**–4.71 (–8.84 to –0.58) *p* = 0.025**	0.18 (–3.07 to 3.42) *p* = 0.915	0.067
CGI	0.50 (–0.03 to 1.03) *p* = 0.065	–0.18 (–0.59 to 0.22) *p* = 0.371	**0.045**
SDS			
Impairment at work	0.52 (–0.39 to 1.44) *p* = 0.263	–0.02 (–0.74 to 0.70) *p* = 0.964	0.360
Impairment in home relationships	0.63 (–0.09 to 1.35) *p* = 0.086	–0.20 (–0.77 to 0.36) *p* = 0.481	0.073
Impairment in family responsibilities	0.51 (–0.26 to 1.29) *p* = 0.193	–0.25 (–0.85 to 0.36) *p* = 0.426	0.128
Perceived stress	0.02 (–0.78 to 0.82) *p* = 0.959	0.09 (–0.54 to 0.72) *p* = 0.772	0.890
Perceived social support	–3.24 (–10.78 to 4.29) *p* = 0.398	–3.69 (–9.61 to 2.24) *p* = 0.222	0.927

Abbreviations: AT, apparent temperature; MDD, major depressive disorder; MADRS, Montgomery–Asberg Depression Rating Scale; HAMD, Hamilton Depression Rating Scale; GAF, Global Assessment of Functioning; CGI, Clinical Global Impression; SDS, Sheehan Disability Scale; β, beta estimate; 95% CI, confidence interval at 95% level.

**Table 8.** Stratified estimates (β) by apparent temperature (AT), with corresponding 95% confidence intervals (95% CI), *p*-values, and interaction *p*-values of the association between NO_2_ exposure (10 μg/m^3^ increase) and MDD severity rating scales

MDD rating scale	β (95% CI) *p* value		Interaction *p* value
	AT ≤ first quartile (5.86 °C)	AT > first quartile (5.86 °C)	
MADRS	**2.56 (0.04–5.08) *p* = 0.047**	–0.19 (–2.20 to 1.83) *p* = 0.854	0.093
HAMD	**3.97 (1.52–6.42) *p* = 0.002**	0.77 (–1.18 to 2.73) *p* = 0.437	**0.044**
GAF	**–3.82 (–6.94 to –0.70) *p* = 0.017**	–1.08 (–3.56 to 1.41) *p* = 0.393	0.177
CGI	**0.44 (0.04–0.84) *p* = 0.029**	0.07 (–0.22 to 0.36) *p* = 0.643	0.136
SDS			
Impairment at work	0.40 (–0.27 to 1.07) *p* = 0.244	0.05 (–0.48 to 0.59) *p* = 0.841	0.429
Impairment in home relationships	0.51 (–0.03 to 1.05) *p* = 0.066	0.08 (–0.36 to 0.51) *p* = 0.727	0.220
Impairment in family responsibilities	0.49 (–0.10 to 1.07) *p* = 0.103	0.28 (–0.18 to 0.75) *p* = 0.232	0.593
Perceived stress	0.14 (–0.46 to 0.75) *p* = 0.640	0.19 (–0.29 to 0.67) *p* = 0.438	0.904
Perceived social support	–3.50 (–9.19 to 2.19) *p* = 0.227	–4.40 (–8.95 to 0.14) *p* = 0.058	0.806

Abbreviations: AT, apparent temperature; MDD, major depressive disorder; MADRS, Montgomery–Asberg Depression Rating Scale; HAMD, Hamilton Depression Rating Scale; GAF, Global Assessment of Functioning; CGI, Clinical Global Impression; SDS, Sheehan Disability Scale; β, beta estimate; 95% CI, confidence interval at 95% level.

## Discussion

To the best of our knowledge, DeprAir is the first study analyzing the relationship between air pollution exposure and severity of depressive symptoms, taking into account the possible role of hypersusceptibility and temperature as effect modifiers.

The characteristics of the study population mirror the ones described in other epidemiological studies on MDD [47–49]: about two-thirds of the enrolled subjects were women and about one-third had a family history of depression. In addition, a significant proportion was affected by obesity and attempted suicide at least once. In line with previous studies, the most frequent subtype of MDD was depression with symptoms of anxiety among women [50, 51], and melancholic depression among men. Furthermore, men were more likely to experience suicidal and addictive behaviors [52].

PM exposure did not directly impact the severity of depressive symptoms, although hypersusceptible subjects were more negatively affected by these pollutants. Of note, all medical conditions used to define hypersusceptibility (at least one among type II diabetes, obesity, hypertension, hypercholesterolemia, and current smoking) are characterized by higher baseline levels of chronic inflammation [53] and might thus represent a flourishing soil for PMs to exert their noxious effects. In addition, PM had a greater significant impact on MDD severity when temperatures were very low (below first quartile of exposure). This finding could have several explanations. First, it might be due to the potential synergistic effect of air pollution and temperature on human health, as documented for other health outcomes [54]. Second, low temperatures could be considered a proxy for the winter season (notoriously associated with a higher incidence of depression [55]) as well as a surrogate measure of irradiance, thus suggesting how light exposure could affect depressive symptoms [56, 57]. This latter interpretation is also supported by several studies showing an association between low sunlight, low temperatures, and high levels of PM2.5, deriving from the capacity of solid particles to reflect and refract sunlight [58, 59].

NO_2_ exposure was strongly associated with MDD severity in the whole population and showed higher effects among hypersusceptible subjects and with concomitant exposure to low temperatures. We can surmise that NO_2_ might have a stronger impact on MDD severity since, as a gas, it can more easily cross the alveolar-capillary and blood–brain barriers. Contrary to what expected, we also observed a decrease in the “perceived social domain” of the SDS scale associated to NO_2_ exposure. In order to interpret this apparently counterintuitive finding, we must acknowledge that the question feeding this domain can be considered ambiguous: technically, in this domain a higher percentage of needed social support corresponds to a poorer individuals’ social functioning. However, it is also known that a more robust social support may alleviate depressive symptoms and the corresponding deterioration of social/working performances [60]. A decrease in this score might thus also hinder a worsening of MDD severity. As such, we consider the interpretation of the relationship between this specific item and air pollution exposure challenging.

When using data from air pollution monitoring stations to assess our subjects’ exposure, associations for PM2.5 did not change, while results for NO_2_ exposure were stronger if compared to model estimates. A possible explanation for this difference might rely on the fact that monitoring stations lack to capture spatial variability of pollutant data [61], while chemical transport models are able to adequately capture both spatial and temporal variability of such data [62]. Since NO_2_ is a very spatial-dependent traffic-related pollutant [63], results based on the chemical transport model could be considered more reliable. In any case, results from the two methodologies were quite similar, probably also because of the high correlation (i.e., ≈90%) between monitor measurements and model estimates (Supplementary Figure 33).

This study has several strengths. This is the first study investigating whether exposure to air pollution could be an important modifiable environmental factor associated with MDD severity. Moreover, MDD severity and its effect on social functioning were assessed through five different rating scales. Detailed information on demographic and clinical aspects, and lifestyle habits have been collected for each subjects allowing to adjust our estimates for major confounders.

Nonetheless, this study has also limitations. It is a cross-sectional study, which does not allow to establish a cause–effect relationship or analyze longitudinal time trends and it has a limited sample size, which, however, is an intrinsic constraint of studies including case-only subjects. We can also acknowledge, though, that the characteristics of our study population are in line with previous investigations on depressed subjects, as mentioned above. Different raters administered the scales: however, the inter-reliability of the used tools is high [41]. In addition, as a sensitivity analysis we also ran our main models after adjusting for a variable identifying the psychiatrist administering the scale, but the results remained unchanged (results not shown). Finally, we cannot theoretically exclude that also long-term exposure to air pollution might have played a role in influencing both the levels of air pollution in the days preceding recruitment of the study population and the scores of the administered MDD rating scales. However, as a sensitivity analysis, we ran our main models also adding the exposure for lag 0–365 (i.e., the annual average) as adjustment variable and results were unmodified (results not shown).

In conclusion, the findings of this study confirm that air pollution has a detrimental effect on mental health, contributing to the severity of MDD. In this sense, preventive measures should be applied to limit the damage resulting from air pollution, such as the expansion of green areas in cities [64] or the monitoring of the mental health of the population living in high-risk areas [65]. In addition, future research will have to clarify whether specific biological mechanisms can explain the different risk of worsening of the depressive state in relation to single air pollutants.

## Supporting information

Borroni et al. supplementary material 1 Borroni et al. supplementary material

Borroni et al. supplementary material 2 Borroni et al. supplementary material

## Supplementary material

The supplementary material for this article can be found at http://doi.org/10.1192/j.eurpsy.2024.1767.
