# Supplementary material for: Impact of air pollution exposure on the severity of major depressive disorder: Results from the DeprAir study
Source: Eur Psychiatry. 2024 Sep 27;67(1):e61. doi: 10.1192/j.eurpsy.2024.1767 (PMC11457114; doi:10.1192/j.eurpsy.2024.1767)

**Supplementary Figure 1**

Directed Acyclic Graph (DAG) used to choose variables to adjust for in the main models investigating the association between air pollution and major depressive disorder (MDD) severity.


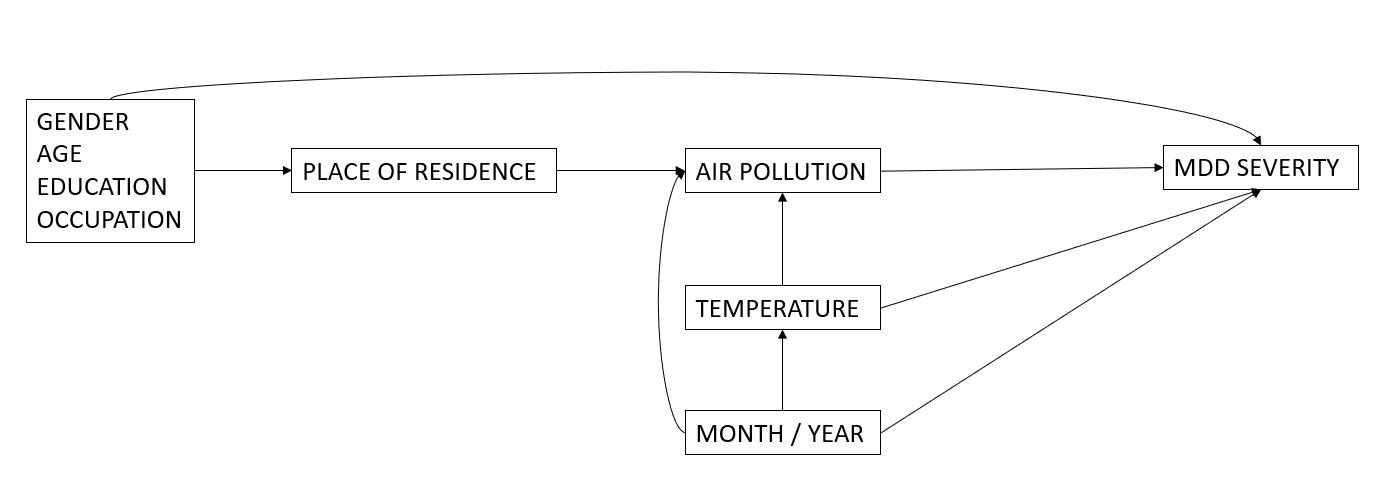


**Supplementary Figure 2**

Trend of PM10 levels in the period of recruitment estimated by the FARM model within the grid cells where the residential addresses of the study population fell. The dashed line corresponds to the World Health Organization 2021 Air Quality Guideline for the pollutant daily average (i.e., 45 µg/m^3^).


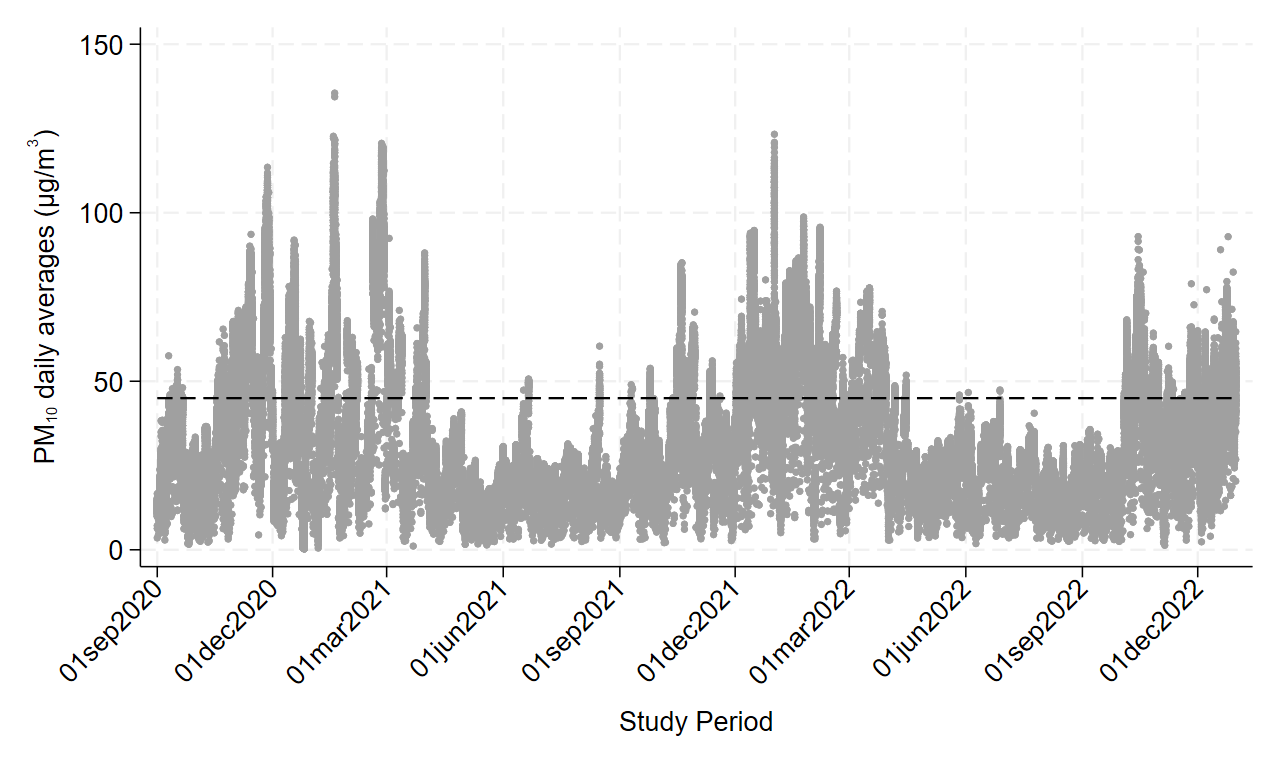


**Supplementary Figures 3-7**

Association between average PM2.5 exposure of the two weeks preceding recruitment and severity of depression, according to the rating scales MADRS (3), HAMD (4), GAF (5), CGI (6), and the five domains of the SDS (Impairment at work: 7a, Impairment in home relationships; 7b, Impairment in family responsibilities: 7c, Perceived stress: 7d, Perceived social support: 7e).

| **Supplementary Figure 3** |
| --- |
| 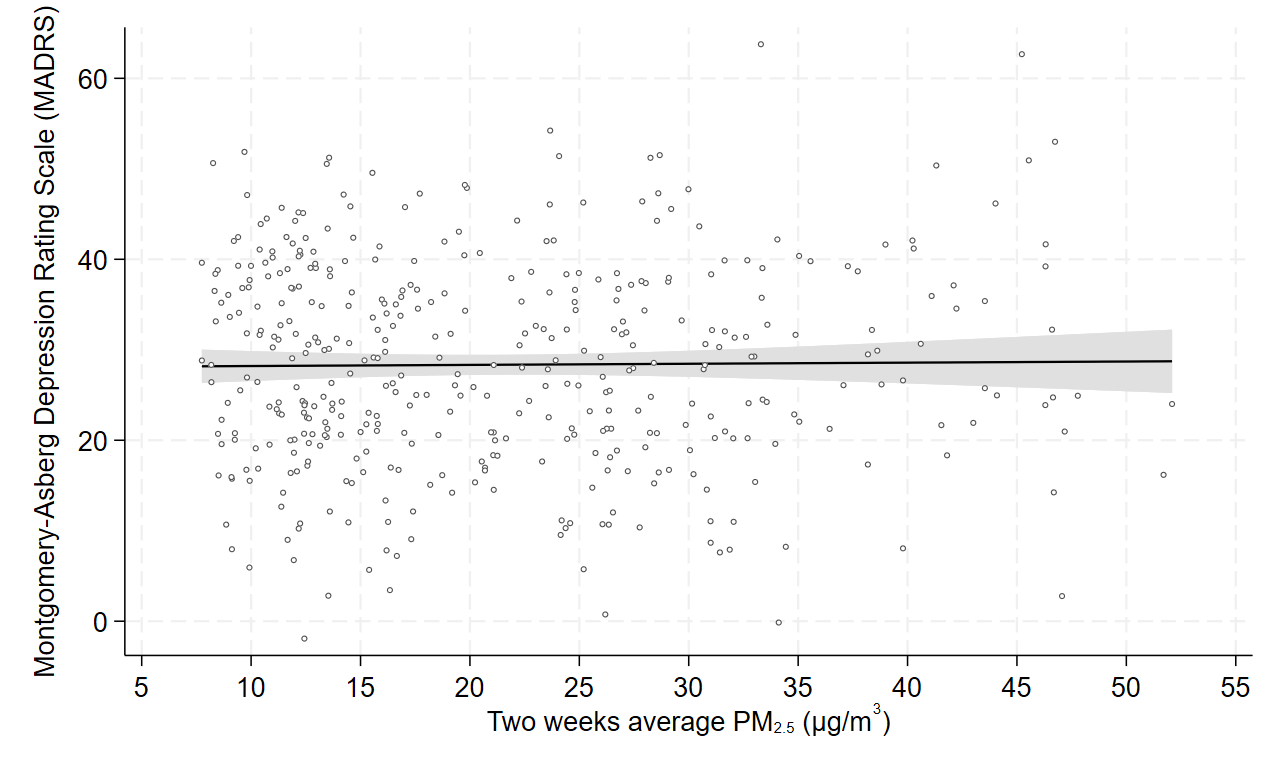 |
|  |
| **Supplementary Figure 4** |
| 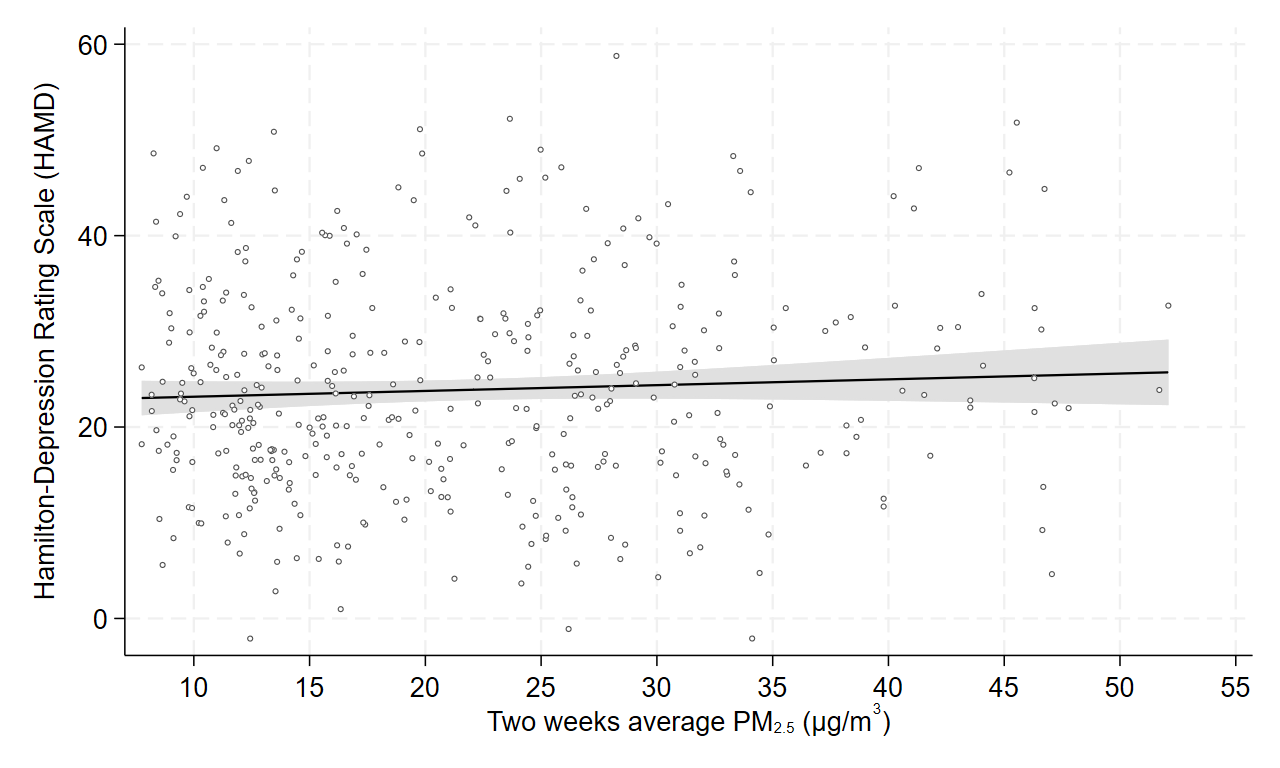 |

| **Supplementary Figure 5** |
| --- |
| 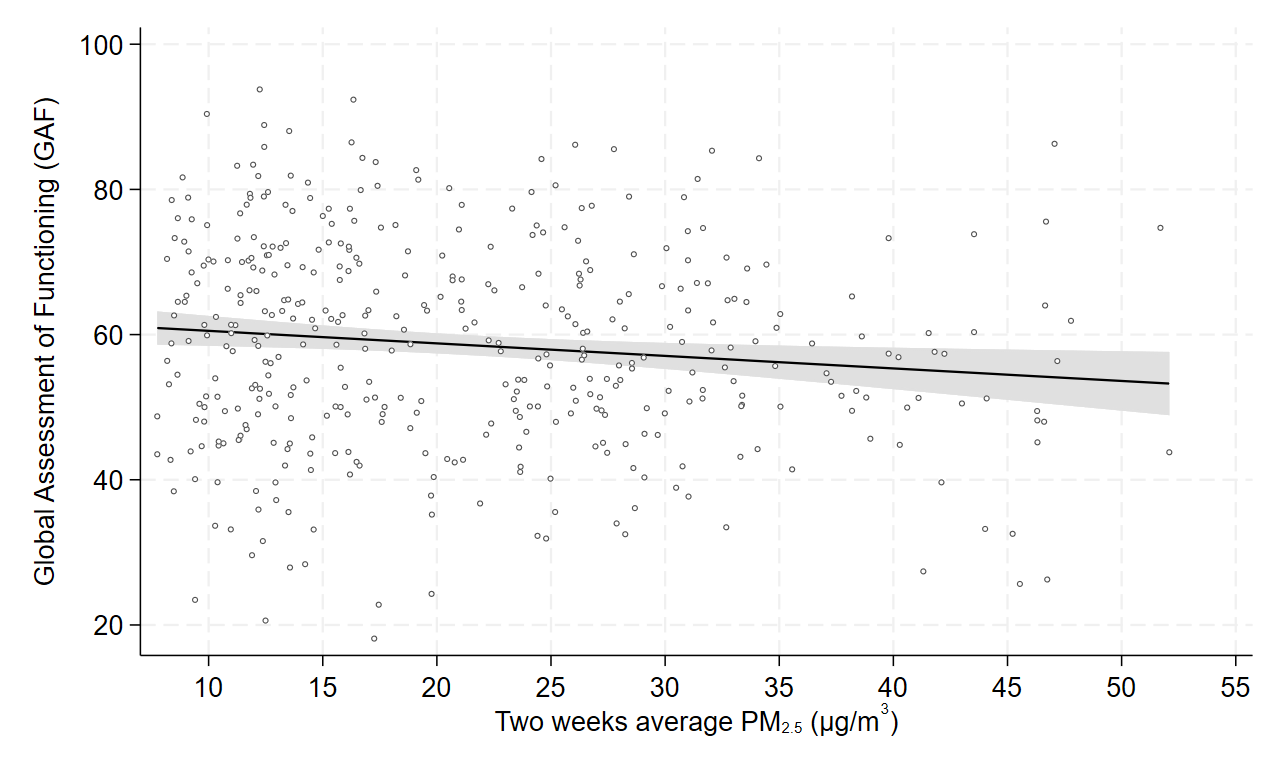 |
|  |
| **Supplementary Figure 6** |
| 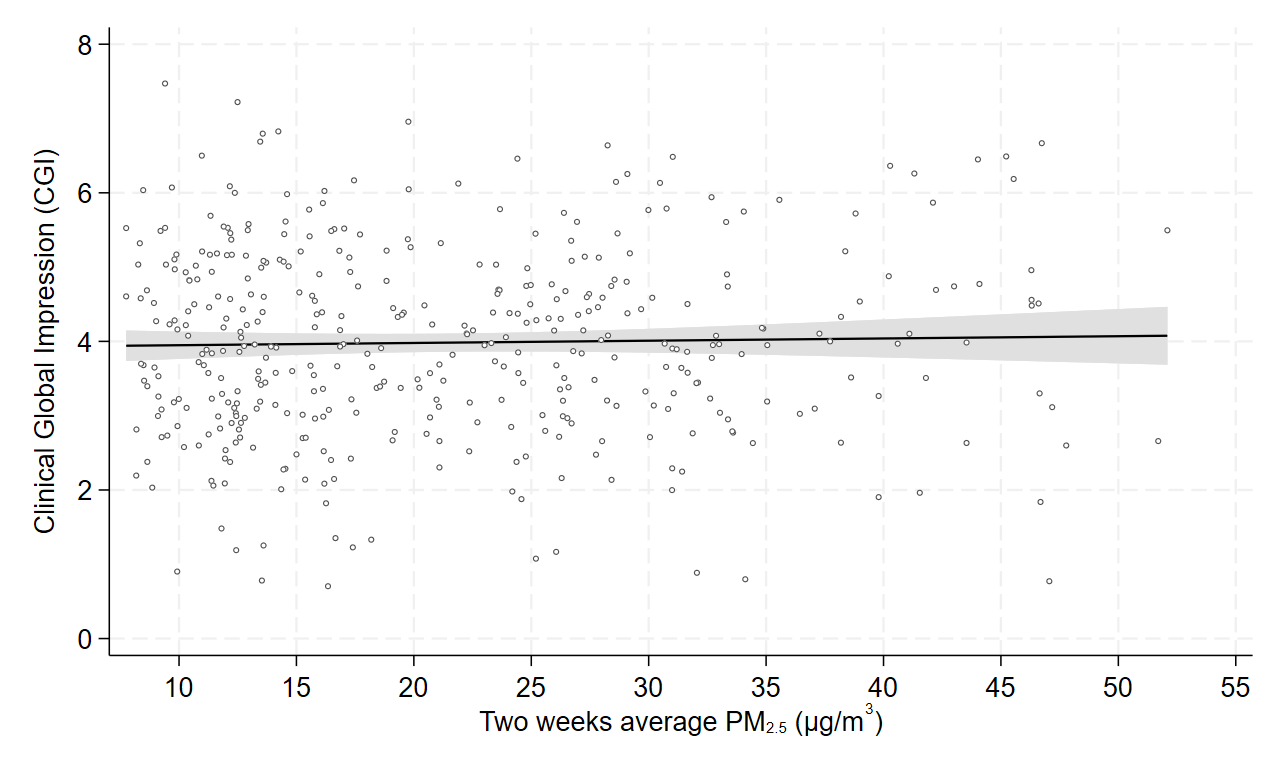 |

| **Supplementary Figure 7a** |
| --- |
| 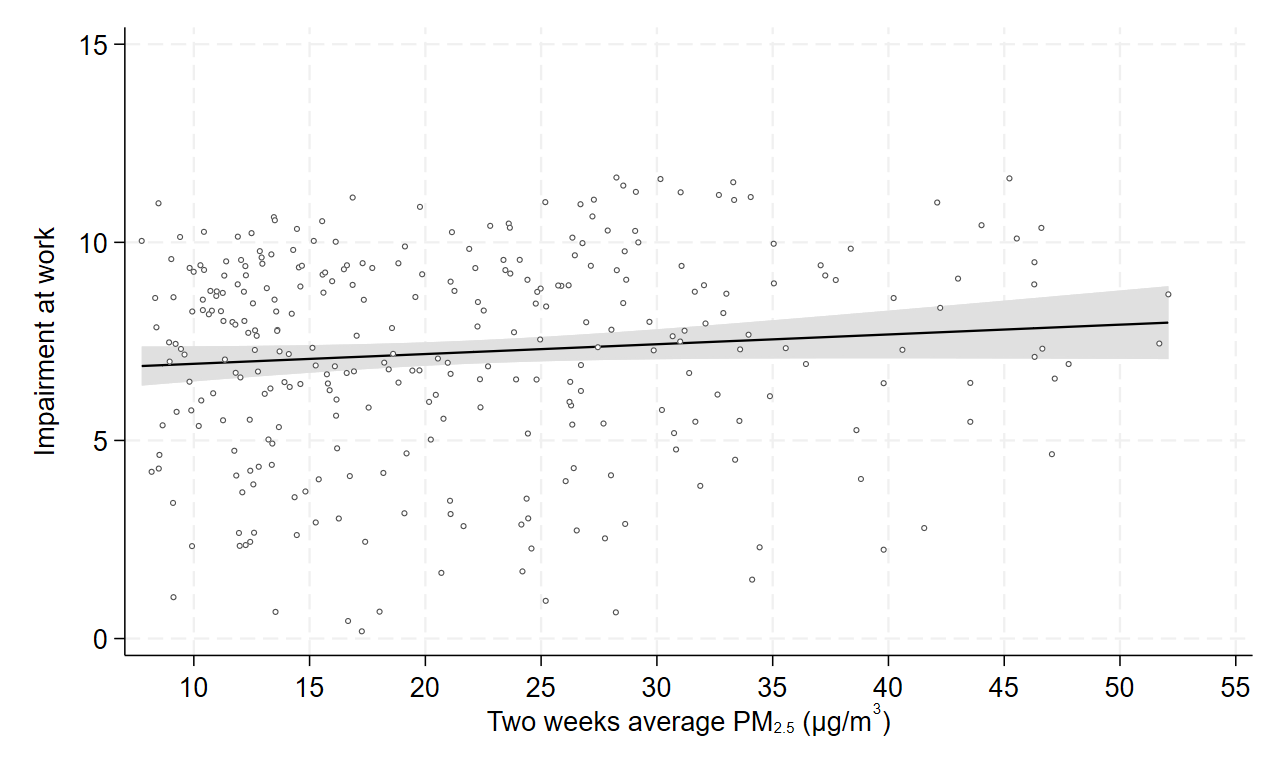 |
|  |
| **Supplementary Figure 7b** |
| 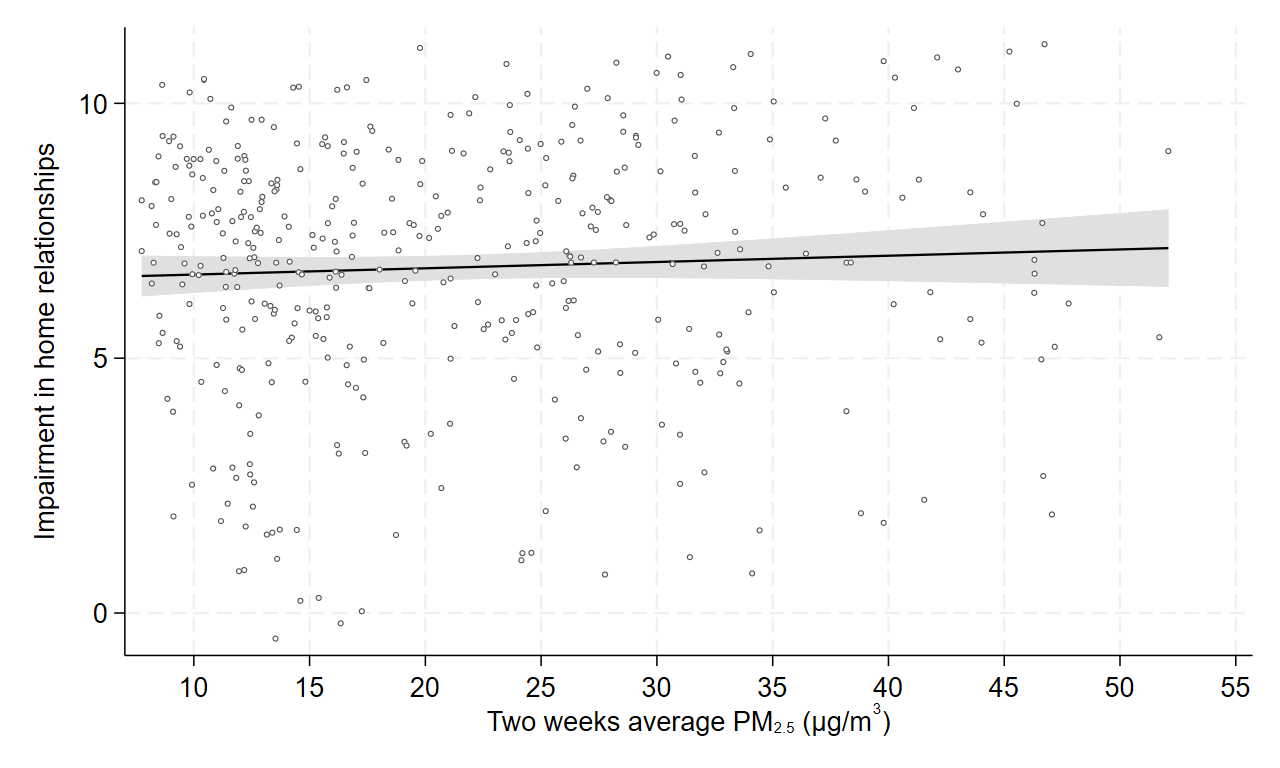 |

| **Supplementary Figure 7c** |
| --- |
| 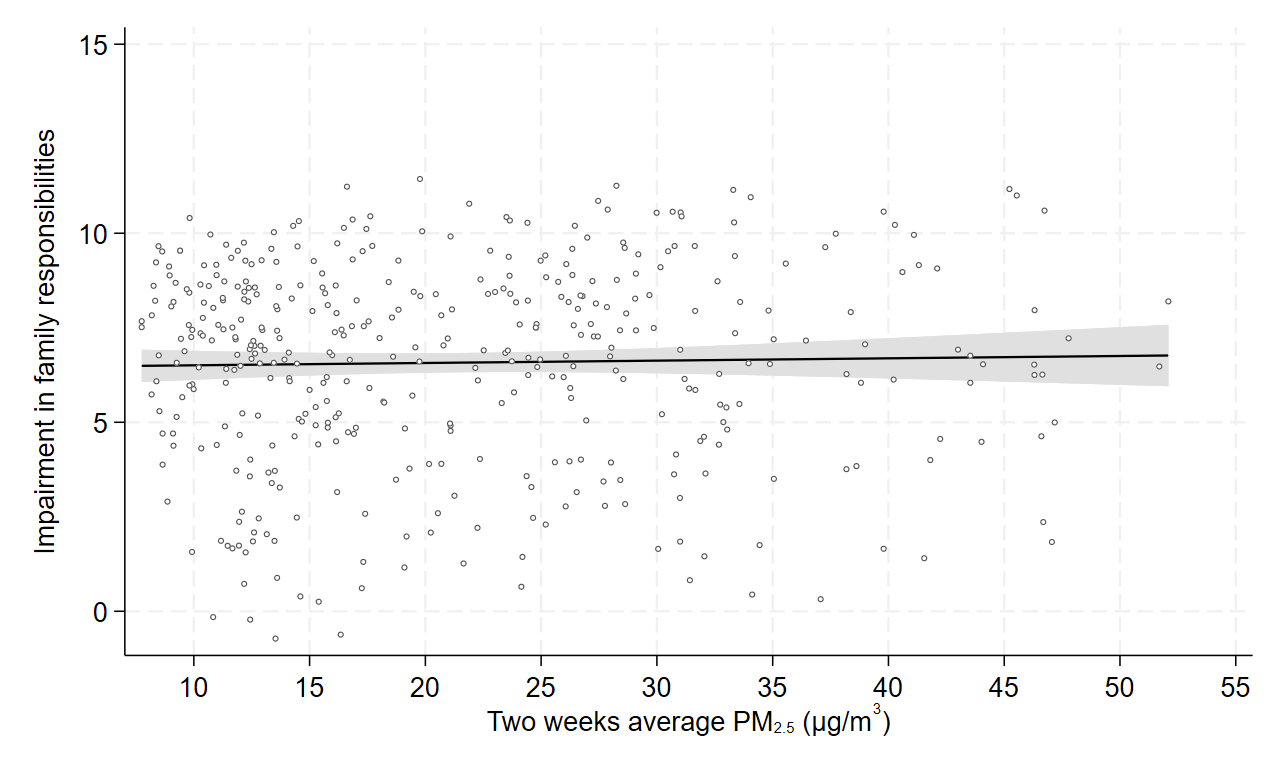 |
|  |
| **Supplementary Figure 7d** |
| 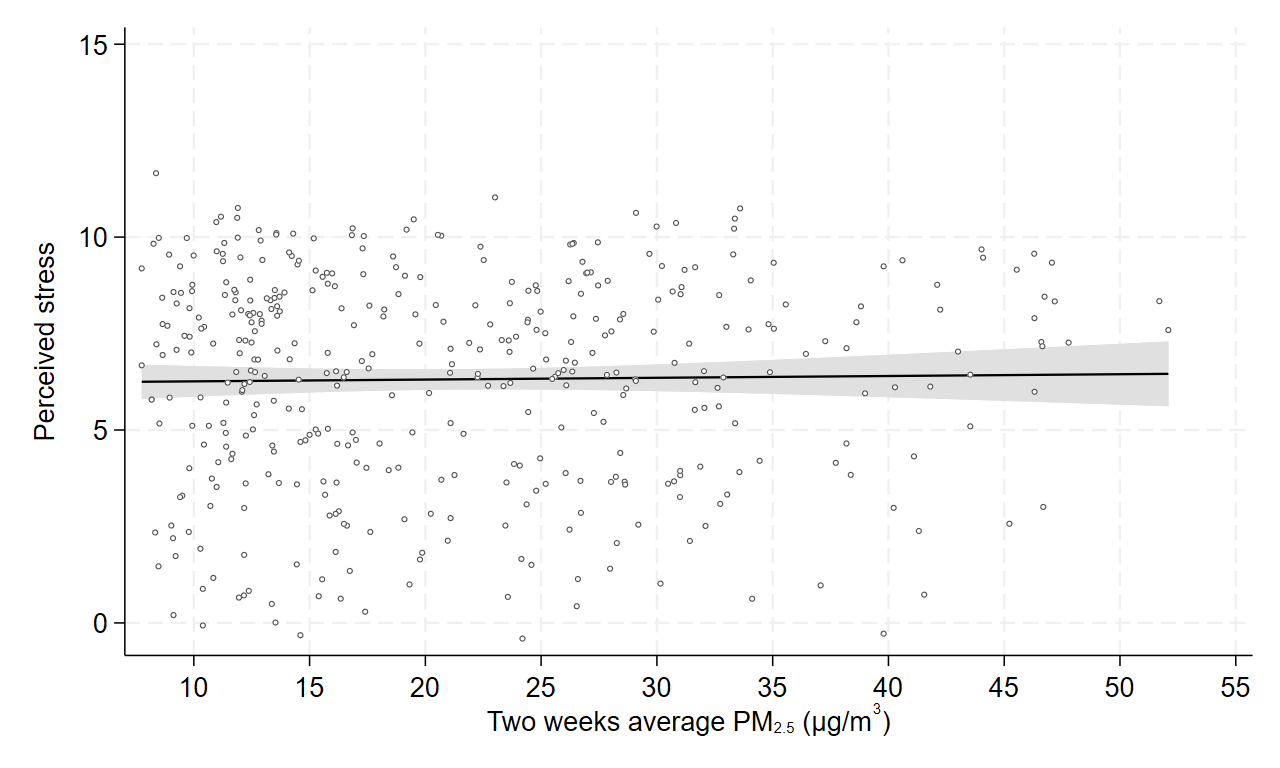 |

| **Supplementary Figure 7e** |
| --- |
| 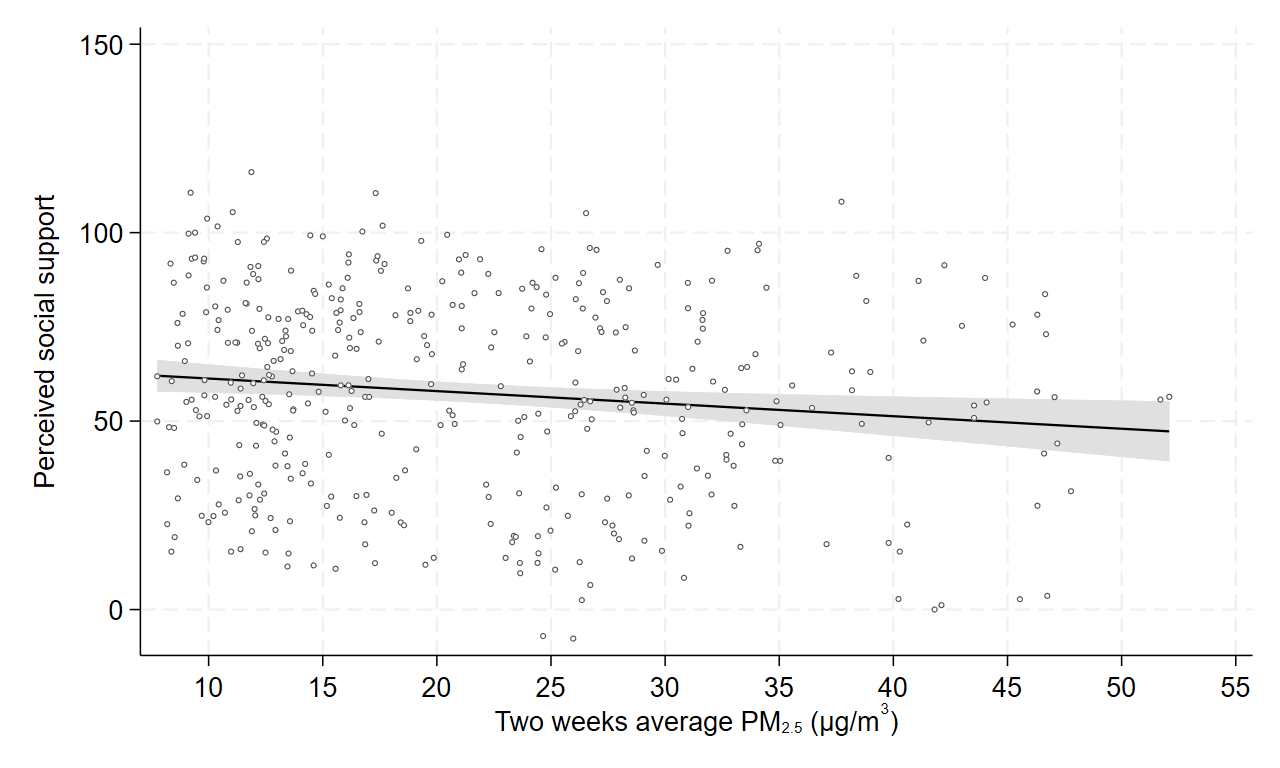 |

**Supplementary Figures 8-12**

Association between average NO_2_ exposure of the two weeks preceding recruitment and severity of depression, according to the rating scales MADRS (8), HAMD (9), GAF (10), CGI (11), and the five domains of the SDS (Impairment at work: 12a, Impairment in home relationships; 12b, Impairment in family responsibilities: 12c, Perceived stress: 12d, Perceived social support: 12e).

| **Supplementary Figure 8** |
| --- |
| 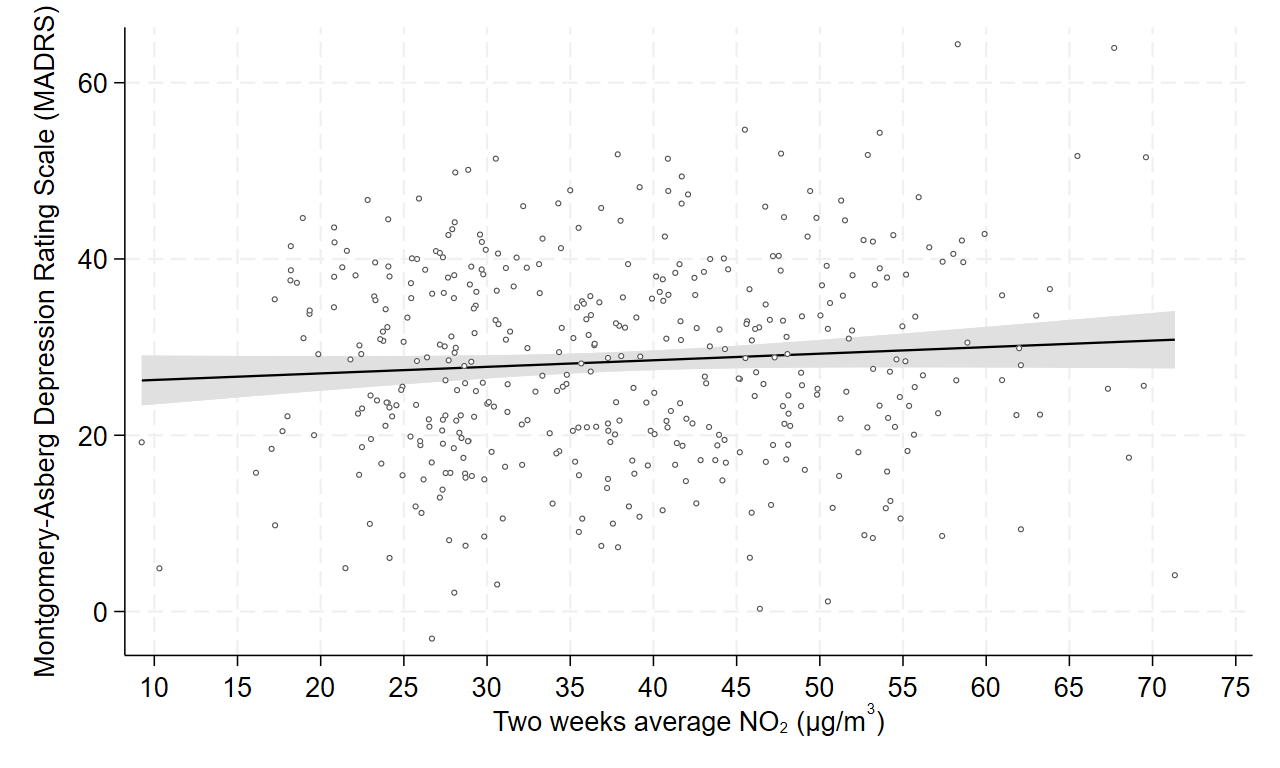 |
|  |
| **Supplementary Figure 9** |
| 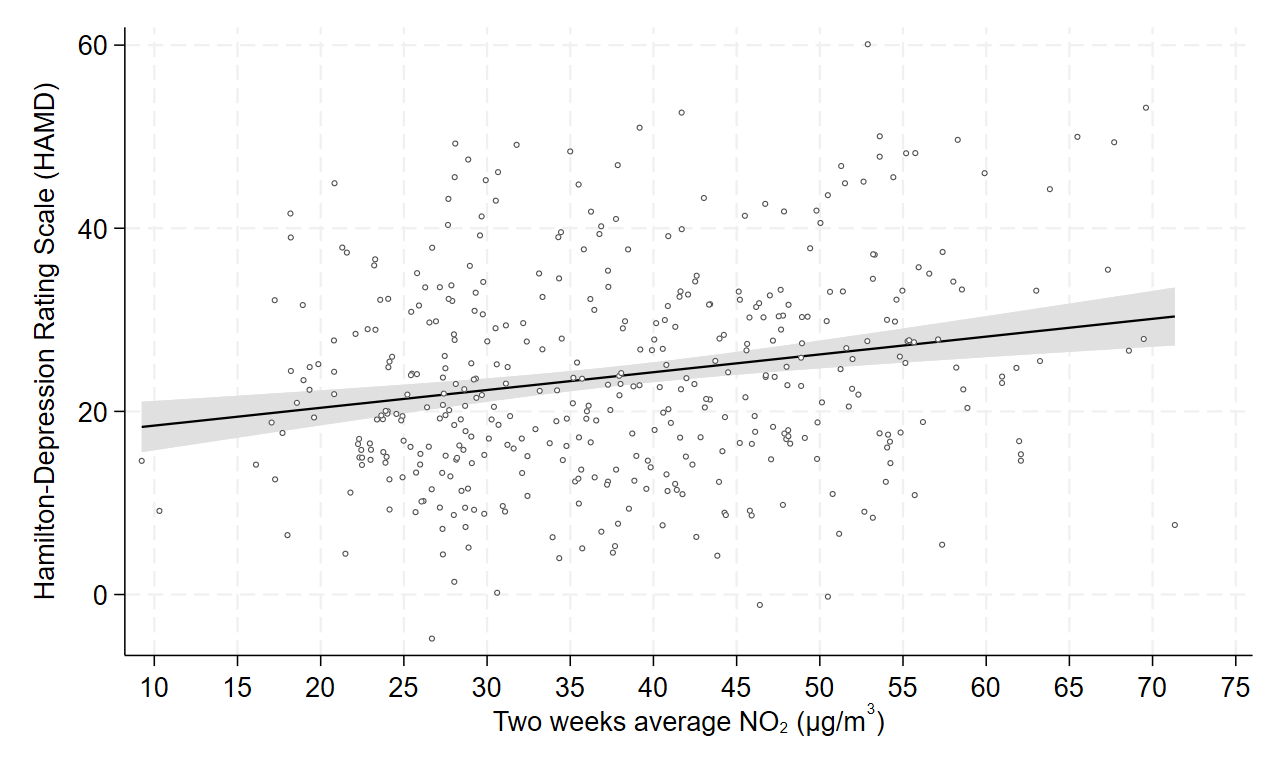 |

| **Supplementary Figure 10** |
| --- |
| 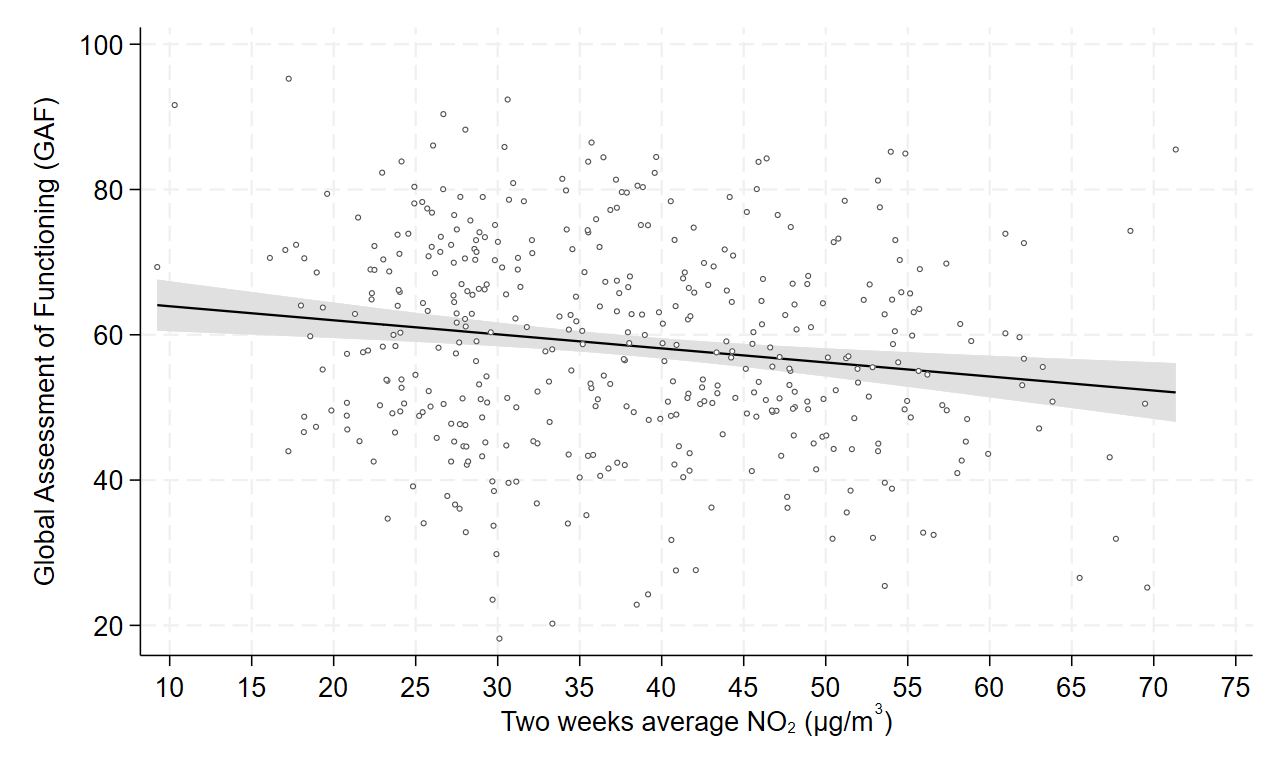 |
|  |
| **Supplementary Figure 11** |
| 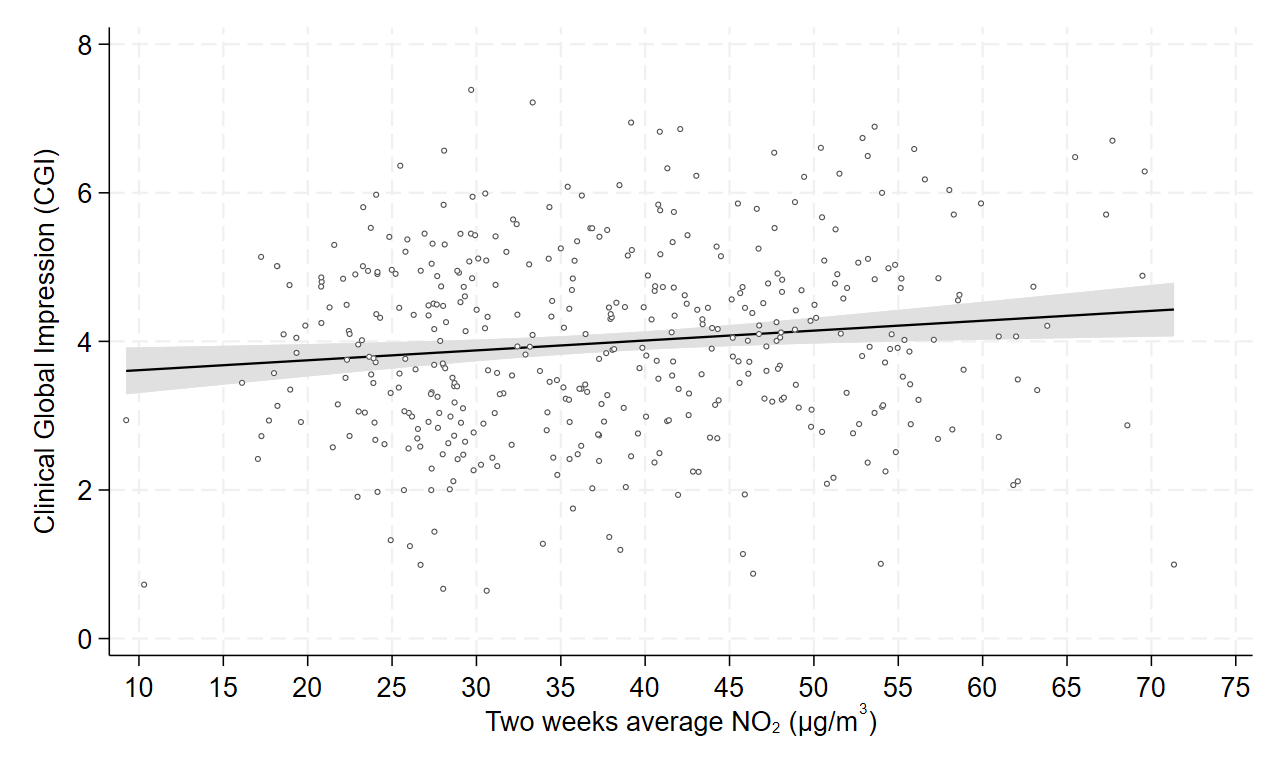 |

| **Supplementary Figure 12a** |
| --- |
| 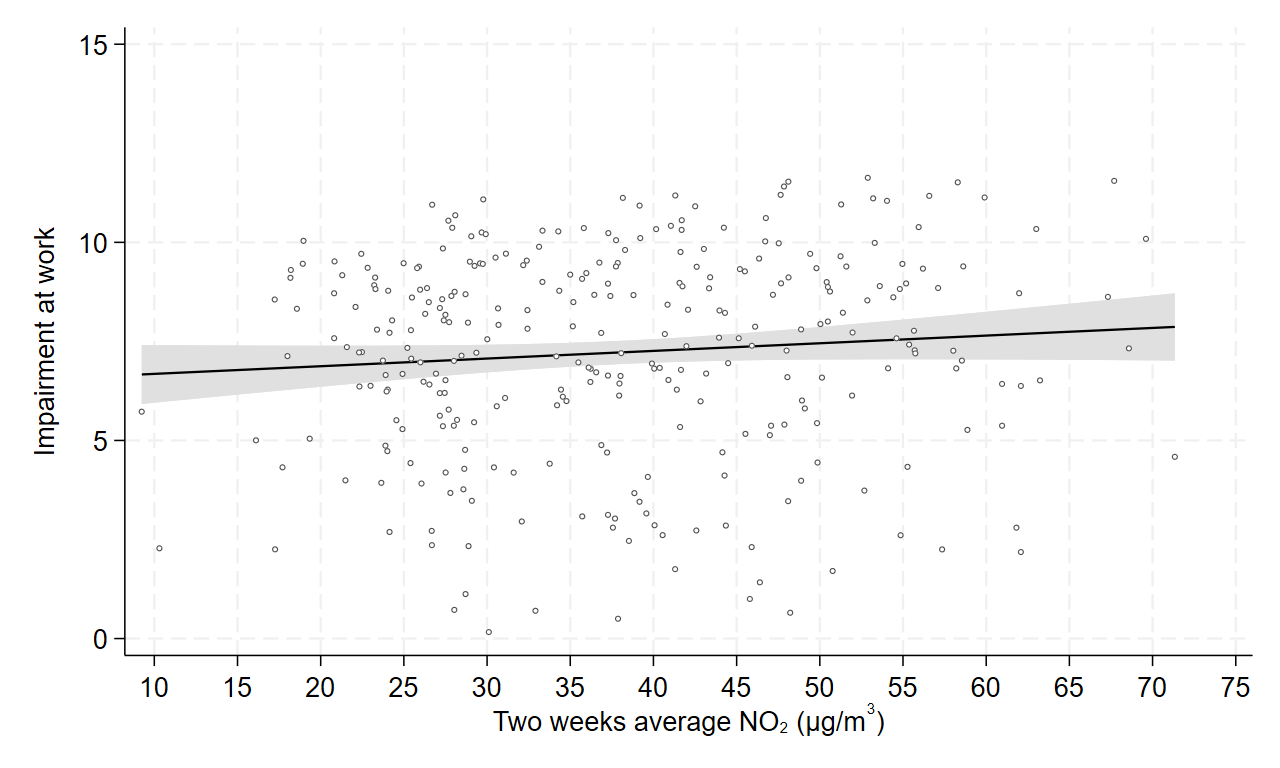 |
|  |
| **Supplementary Figure 12b** |
| 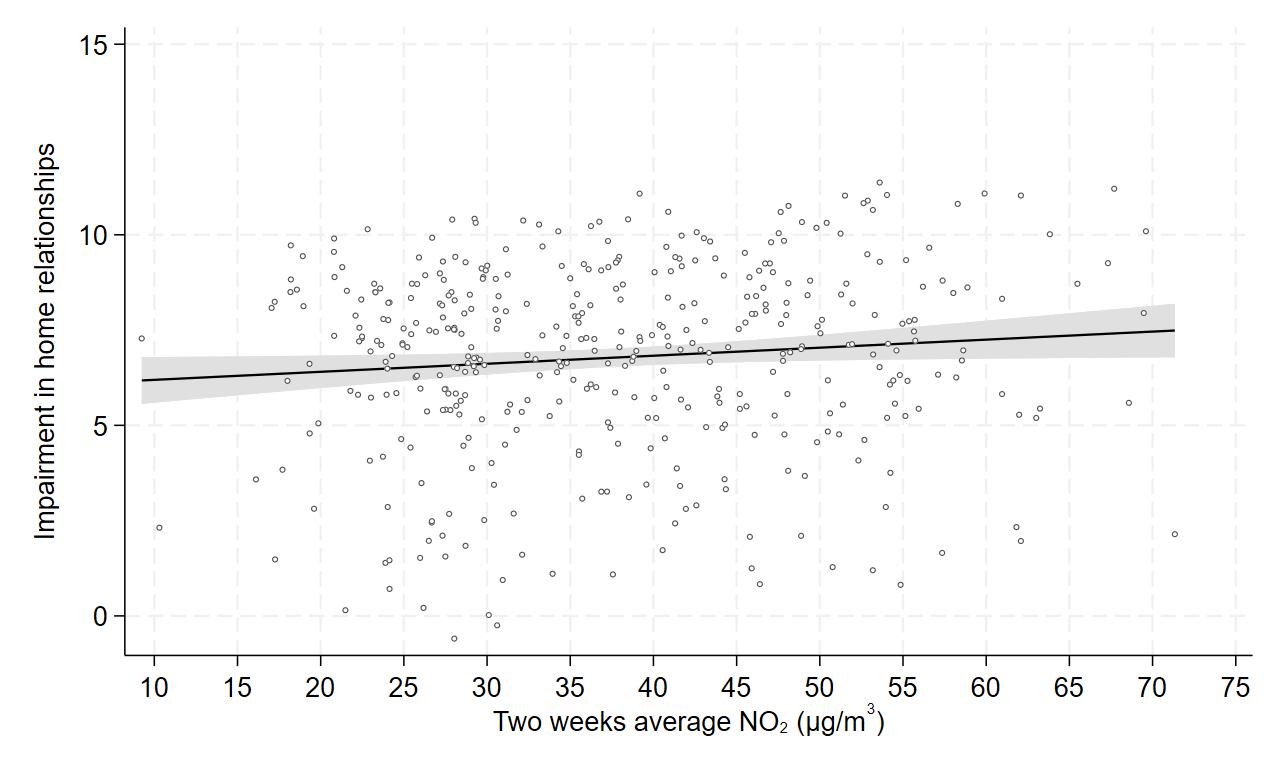 |

| **Supplementary Figure 12c** |
| --- |
| 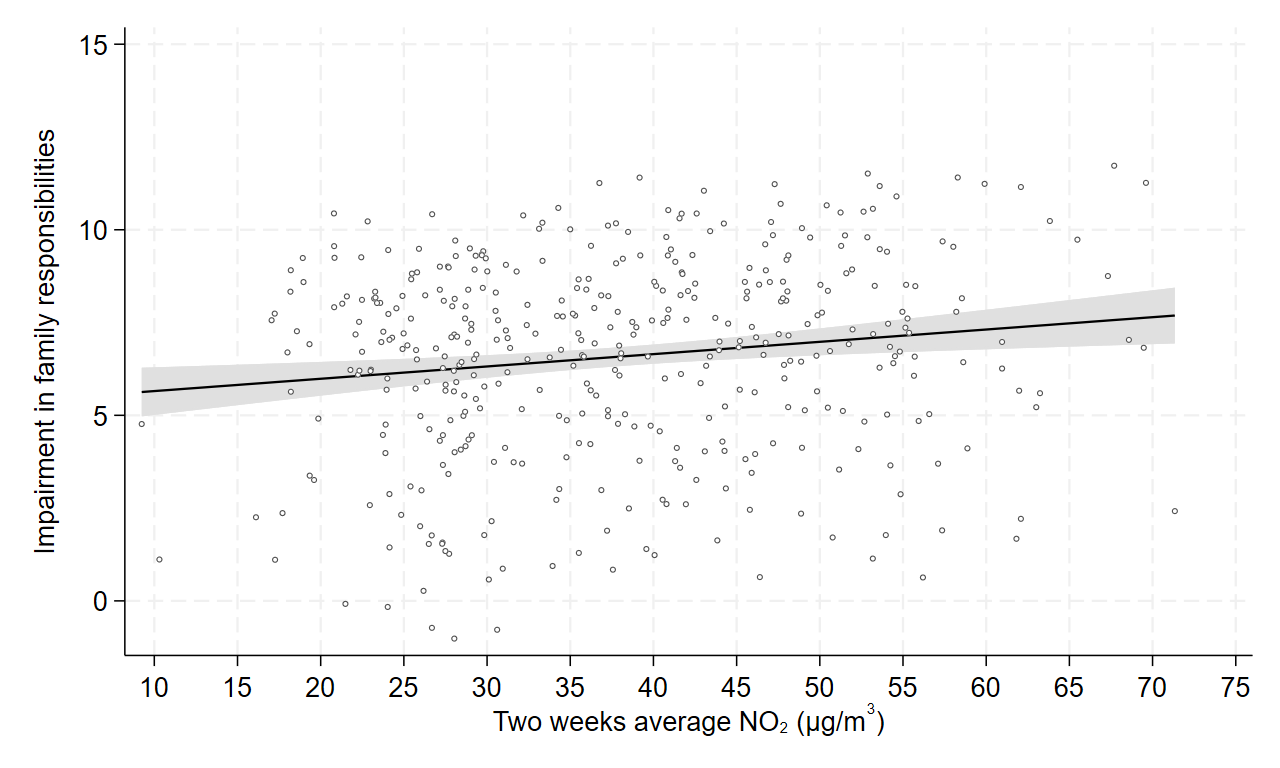 |
|  |
| **Supplementary Figure 12d** |
| 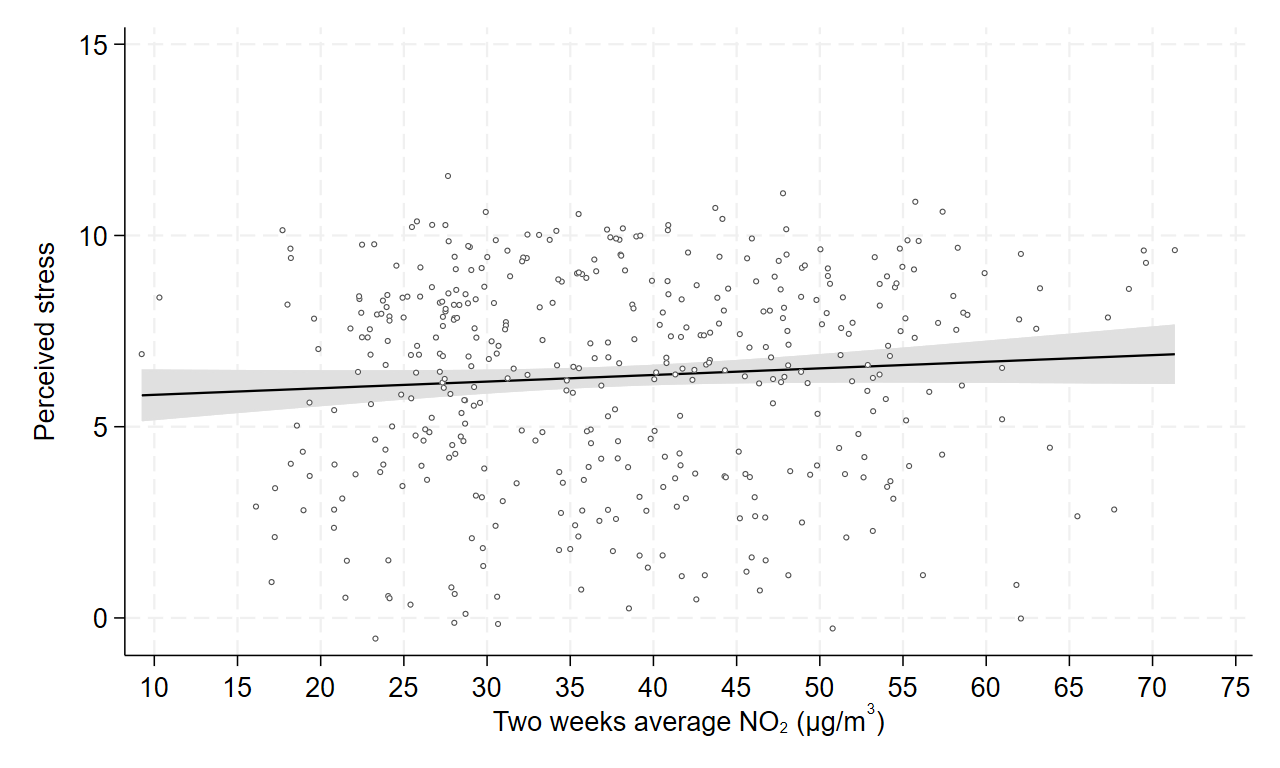 |

| **Supplementary Figure 12e** |
| --- |
| 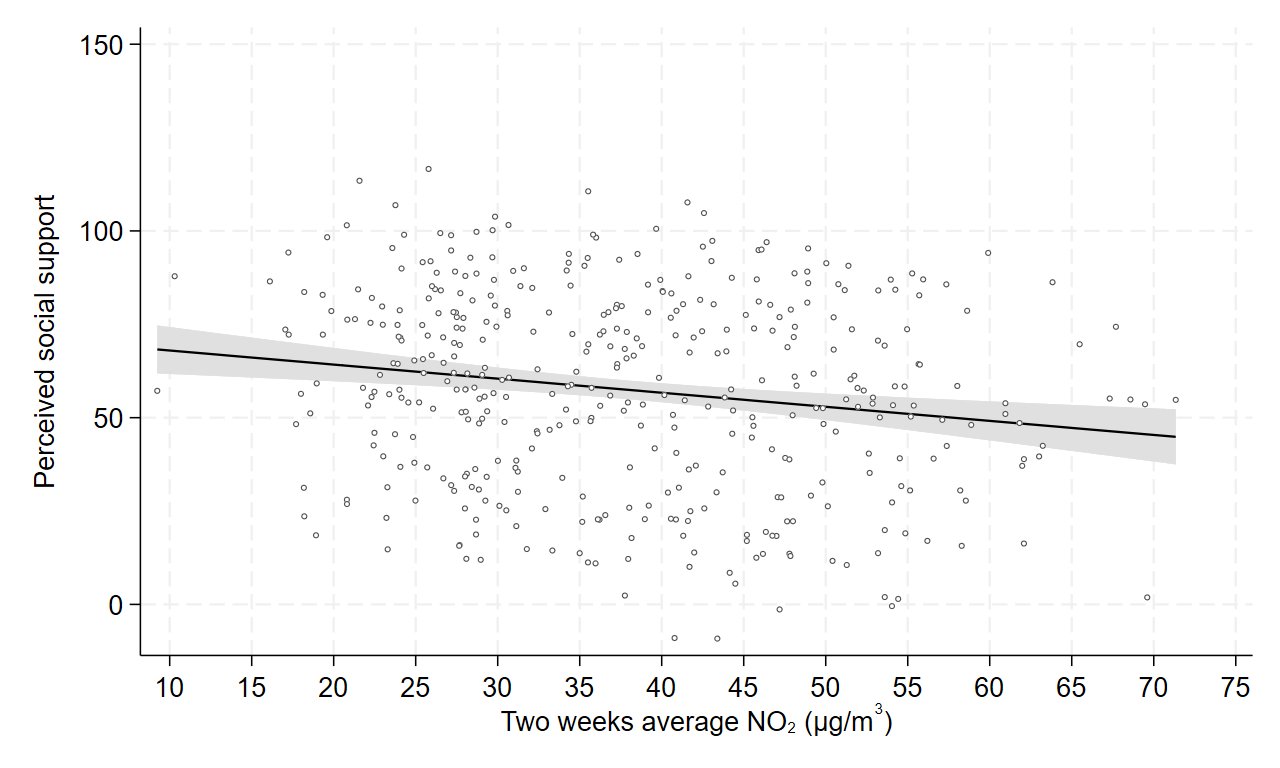 |

**Supplementary Figures 13-17**

Association between average PM2.5 exposure of the two weeks preceding recruitment and severity of depression, stratified by hypersusceptibility status, according to the rating scales MADRS (13), HAMD (14), GAF (15), CGI (16), and the five domains of the SDS (Impairment at work: 17a, Impairment in home relationships; 17b, Impairment in family responsibilities: 17c, Perceived stress: 17d, Perceived social support: 17e).

| **Supplementary Figure 13** |
| --- |
| 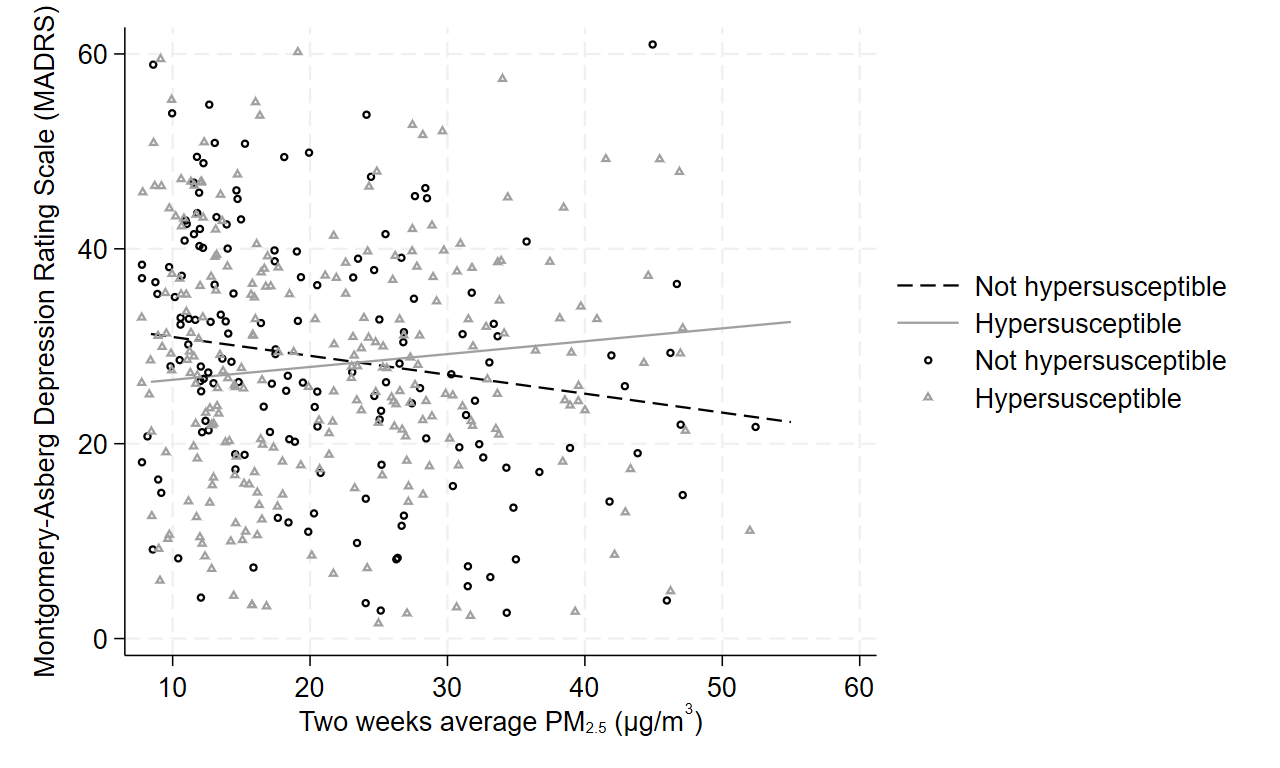 |
|  |
| **Supplementary Figure 14** |
| 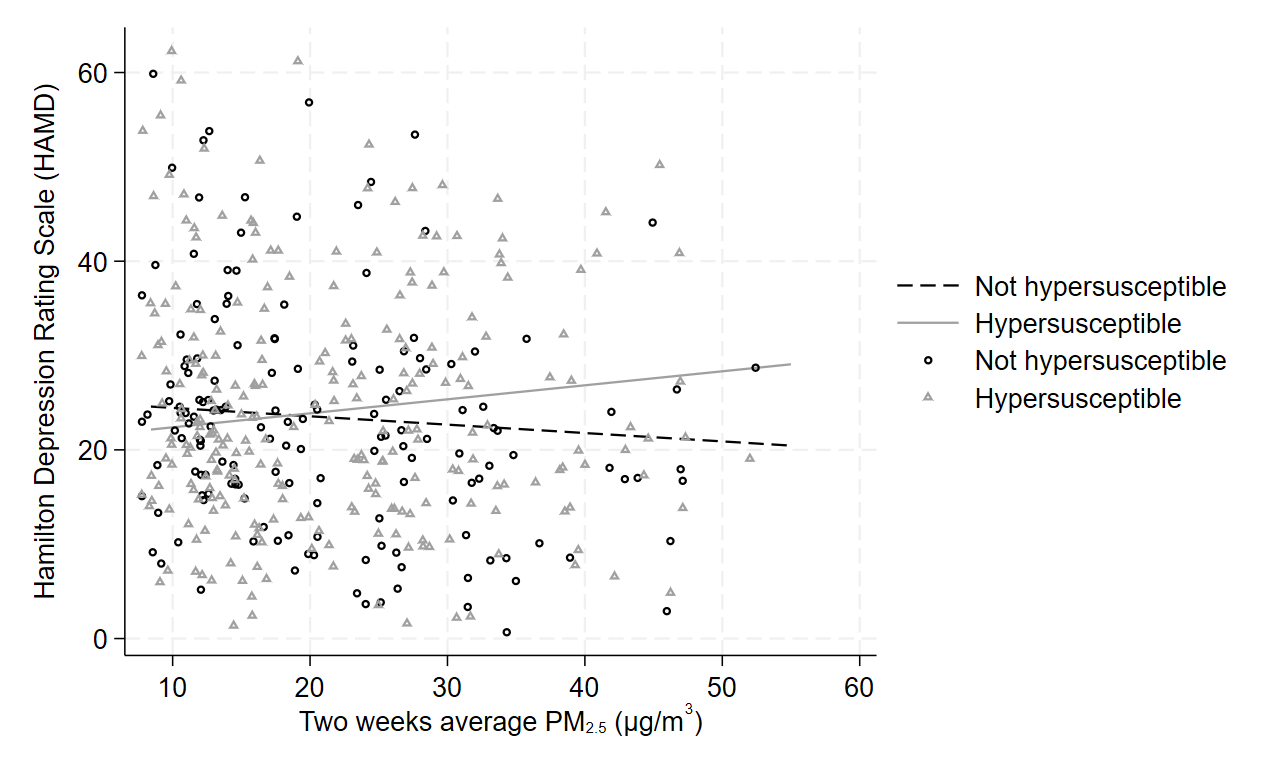 |

| **Supplementary Figure 15** |
| --- |
| 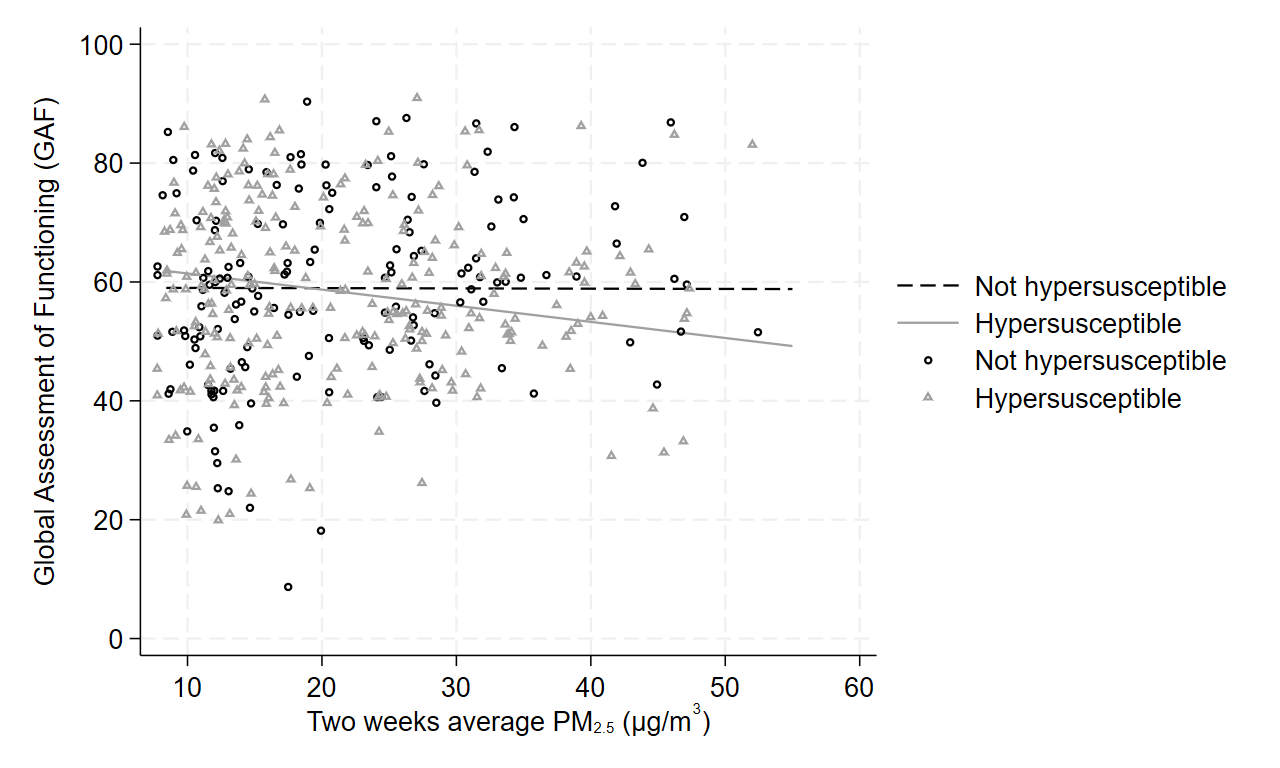 |
|  |
| **Supplementary Figure 16** |
| 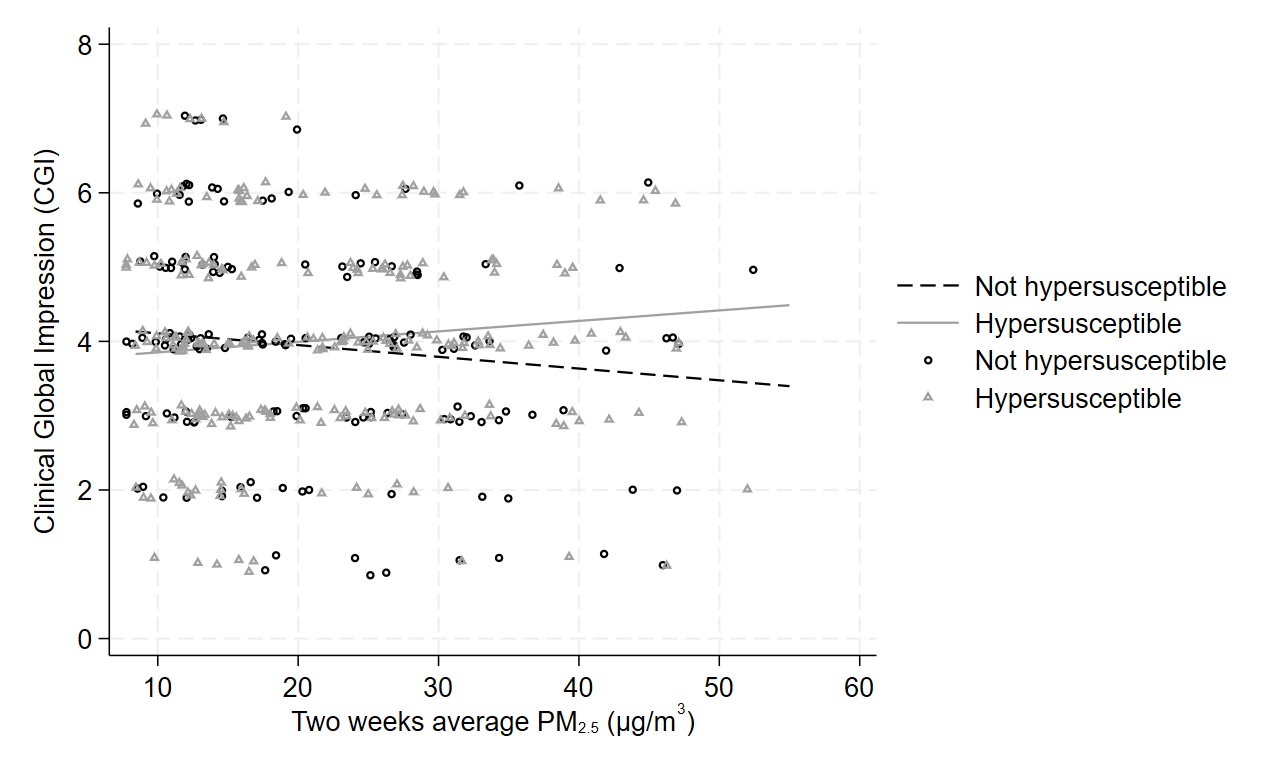 |

| **Supplementary Figure 17a** |
| --- |
| 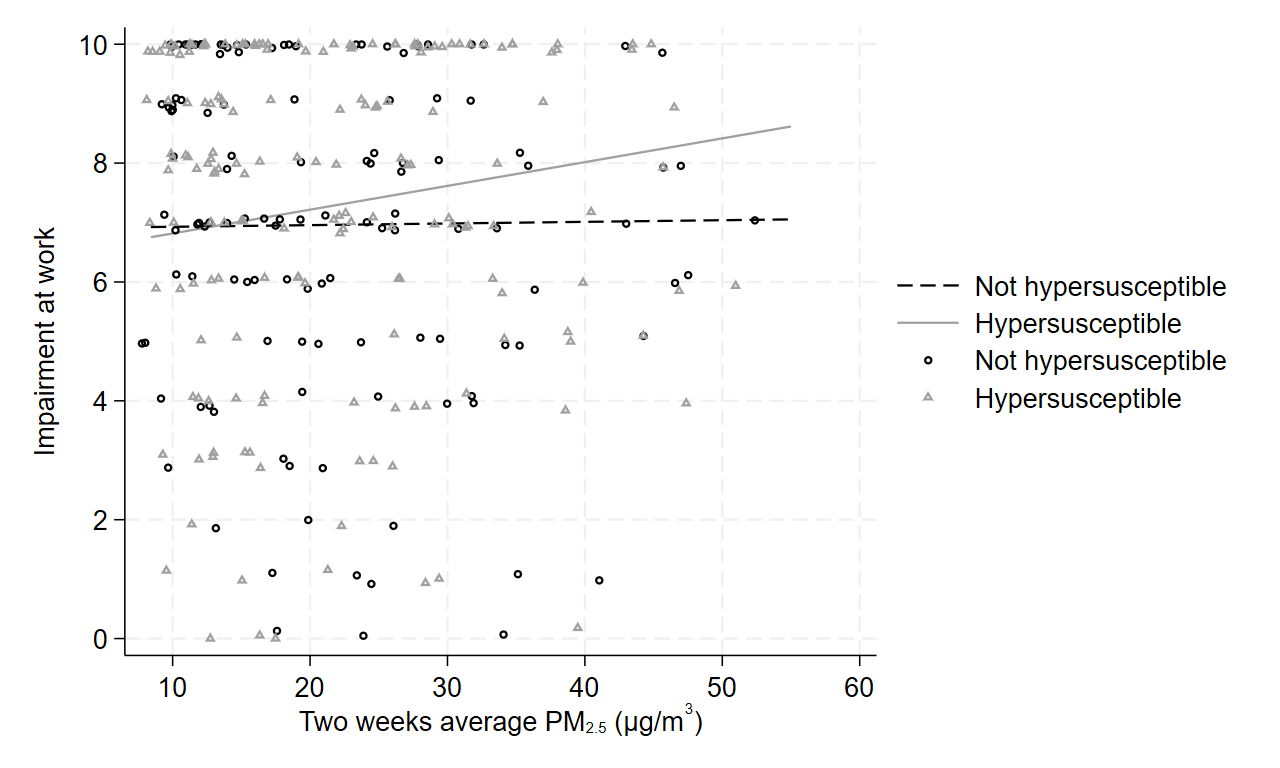 |
|  |
| **Supplementary Figure 17b** |
| 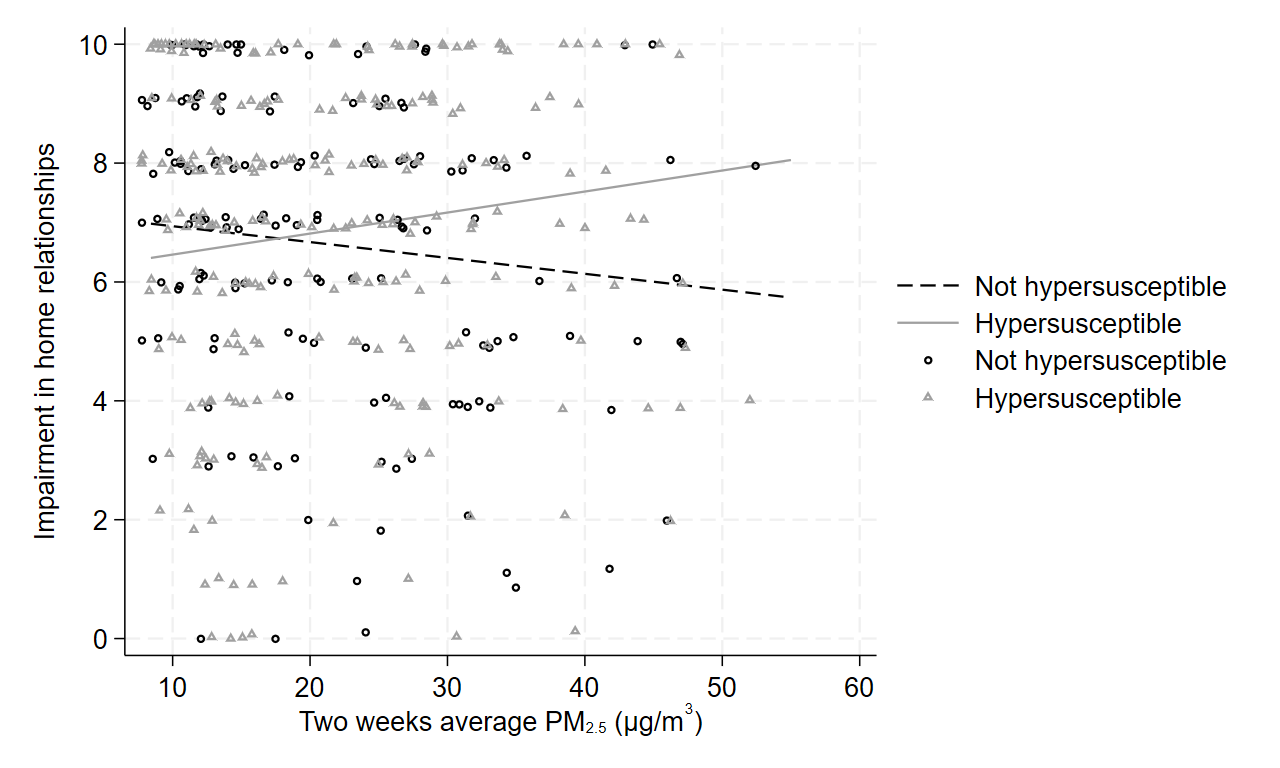 |

| **Supplementary Figure 17c** |
| --- |
| 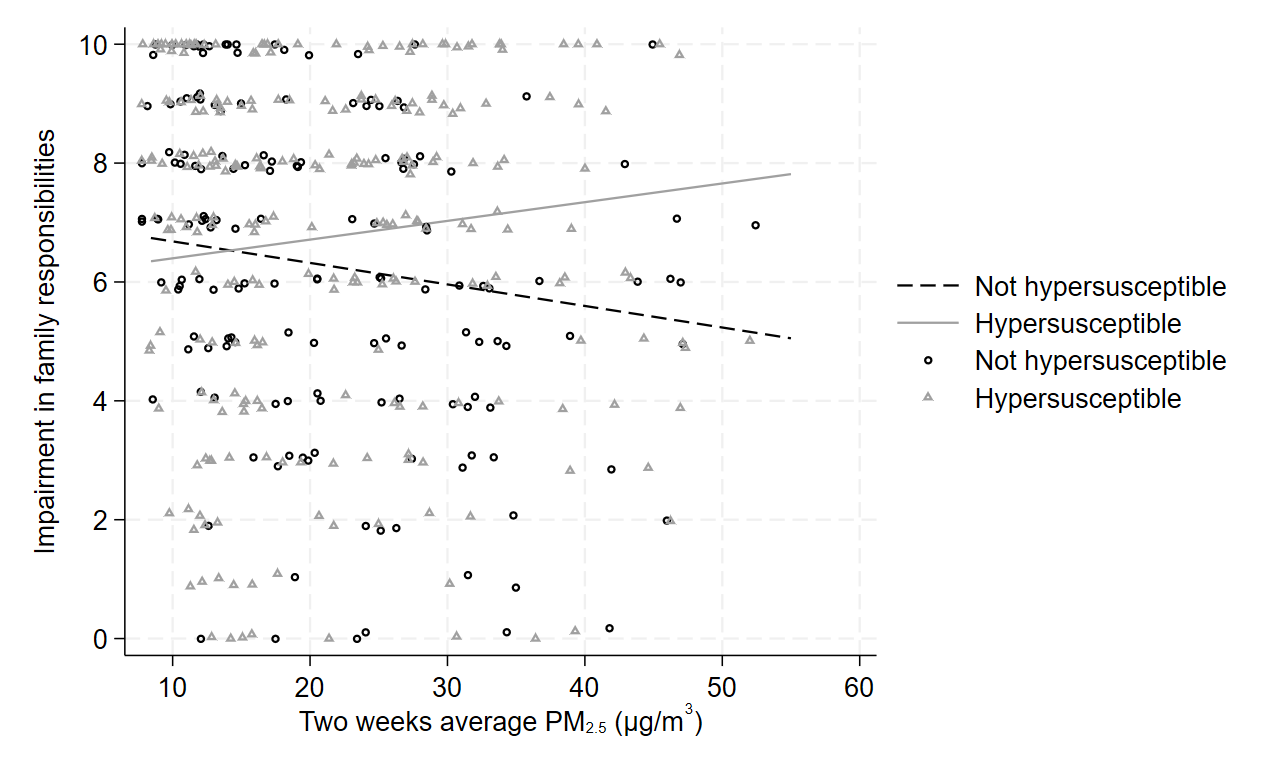 |
|  |
| **Supplementary Figure 17d** |
| 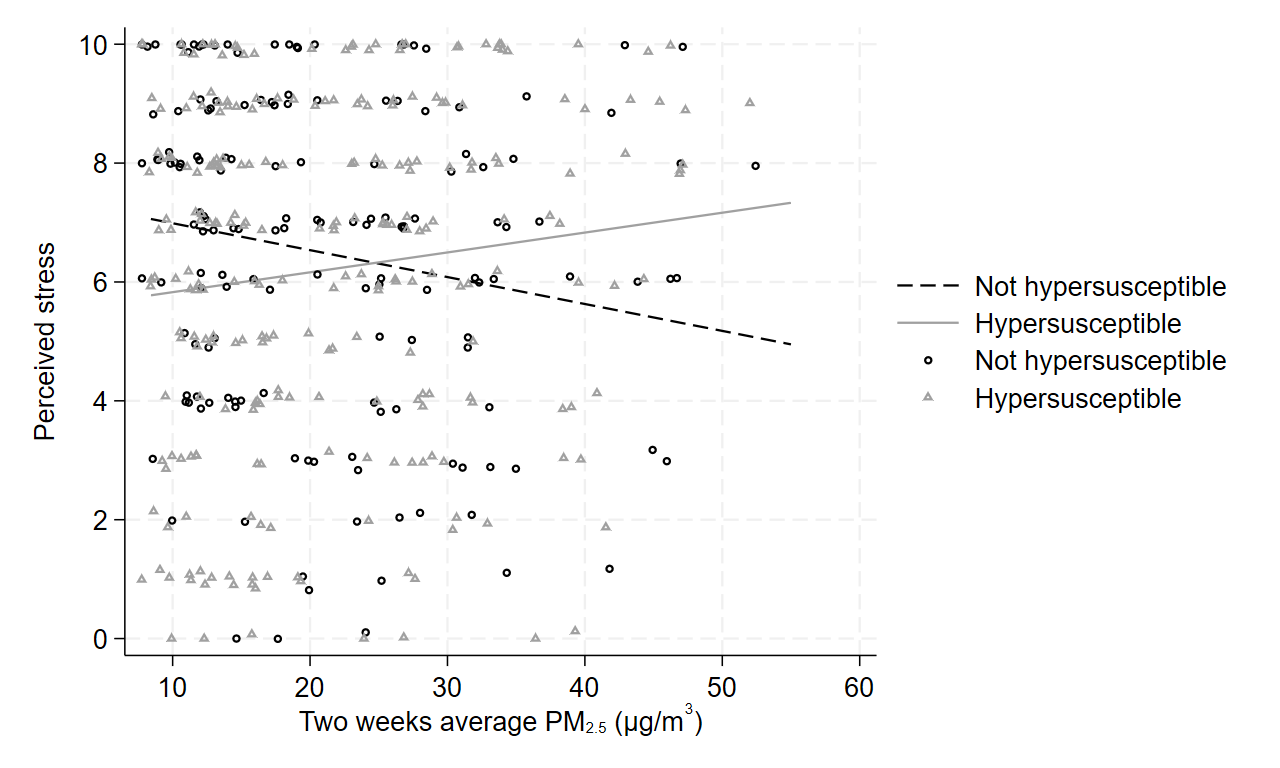 |

| **Supplementary Figure 17e** |
| --- |
| 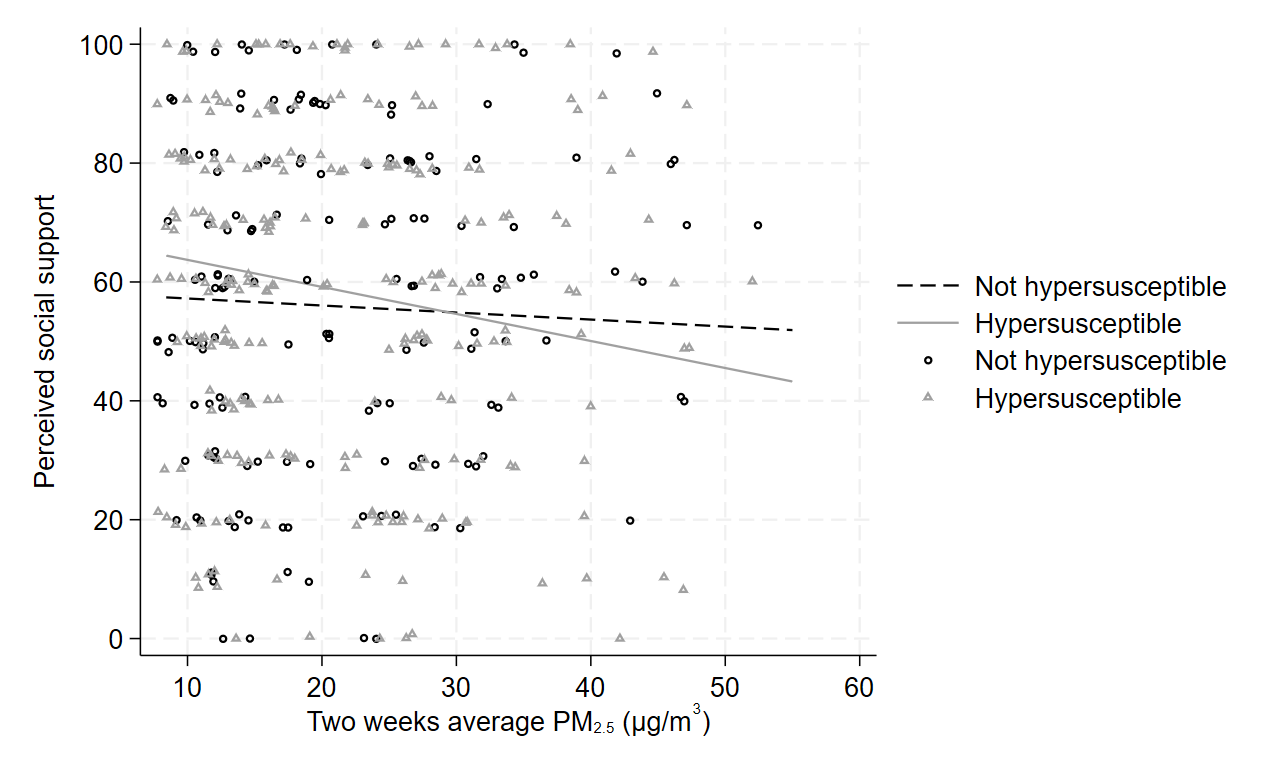 |

**Supplementary Figures 18-22**

Association between average NO_2_ exposure of the two weeks preceding recruitment and severity of depression, stratified by hypersusceptibility status, according to the rating scales MADRS (18), HAMD (19), GAF (20), CGI (21), and the five domains of the SDS (Impairment at work: 22a, Impairment in home relationships; 22b, Impairment in family responsibilities: 22c, Perceived stress: 22d, Perceived social support: 22e).

| **Supplementary Figure 18** |
| --- |
| 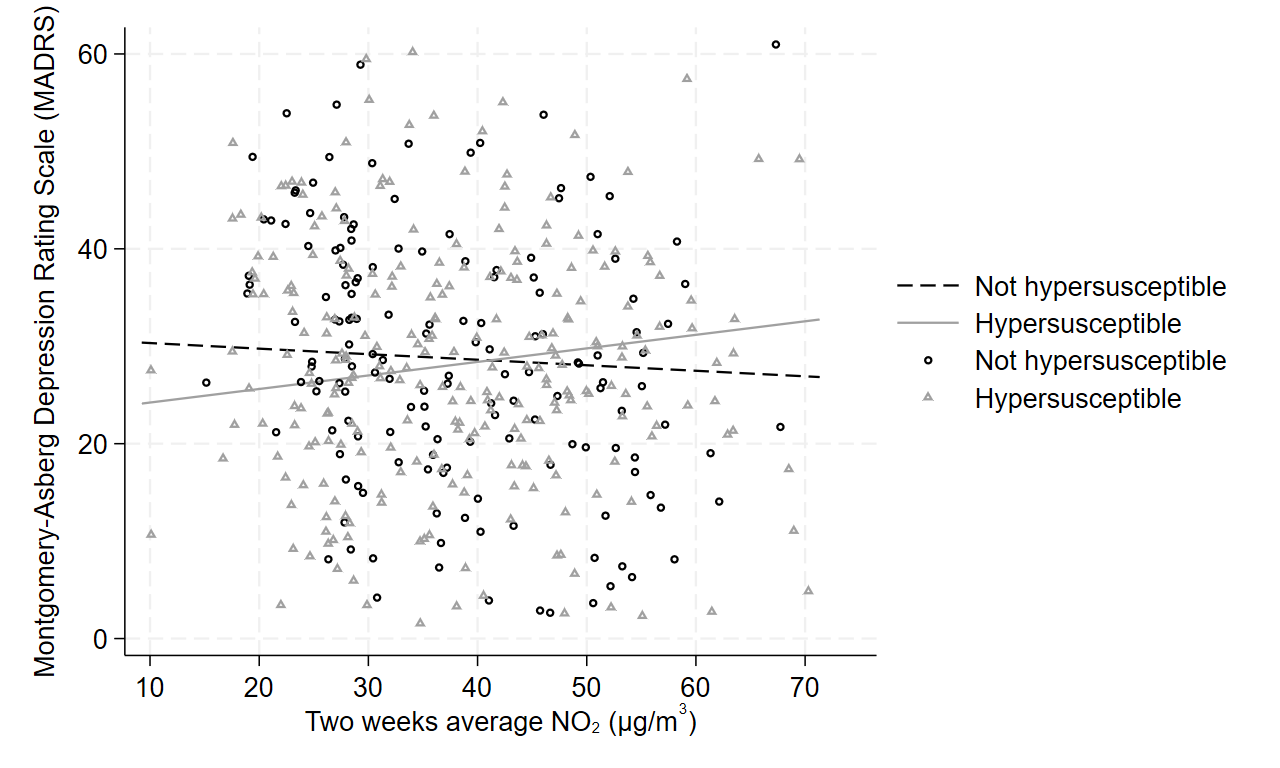 |
|  |
| **Supplementary Figure 19** |
| 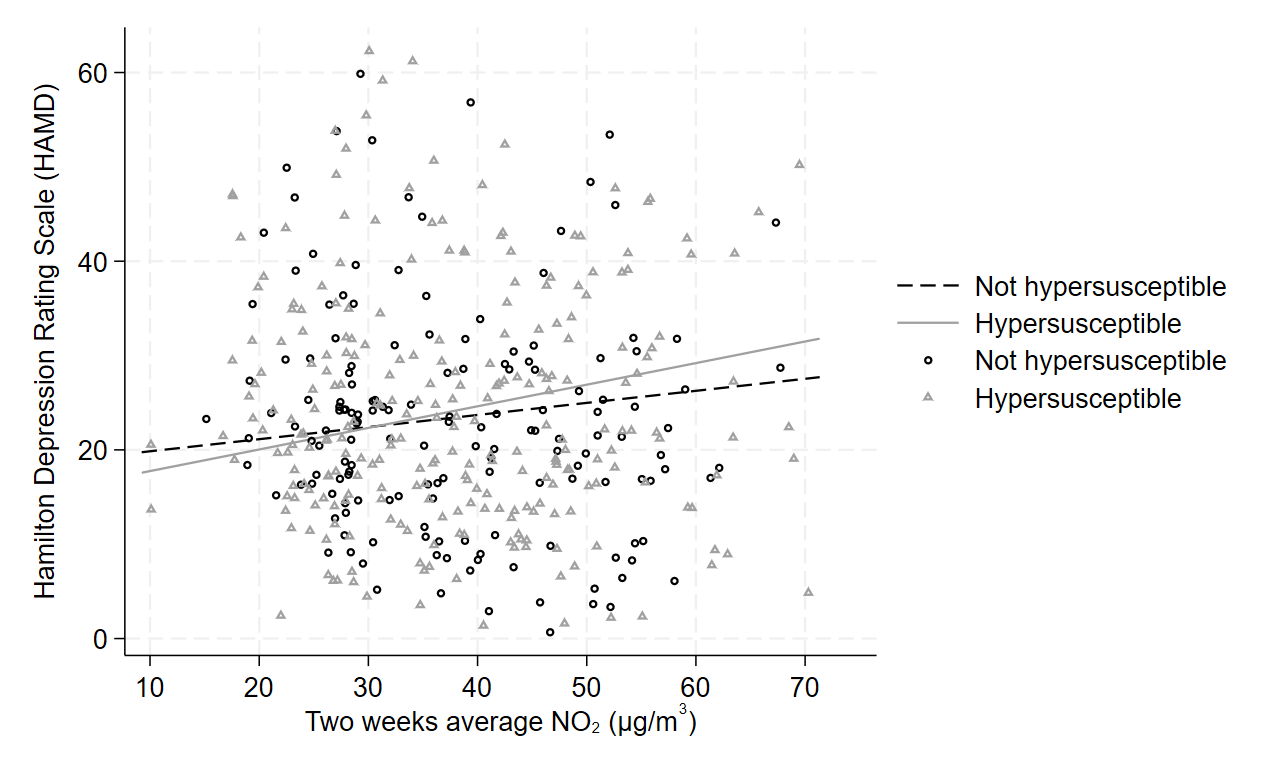 |

| **Supplementary Figure 20**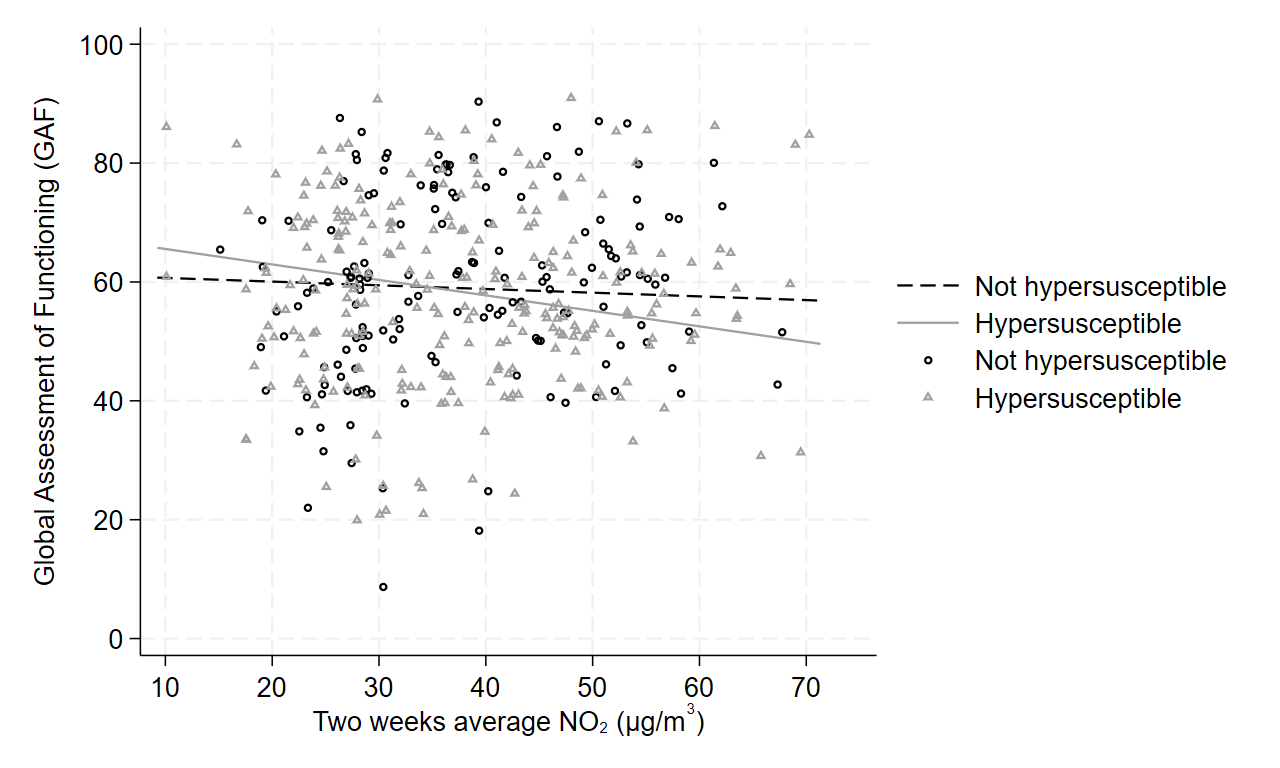 |
| --- |
|  |
|  |
| **Supplementary Figure 21** |
| 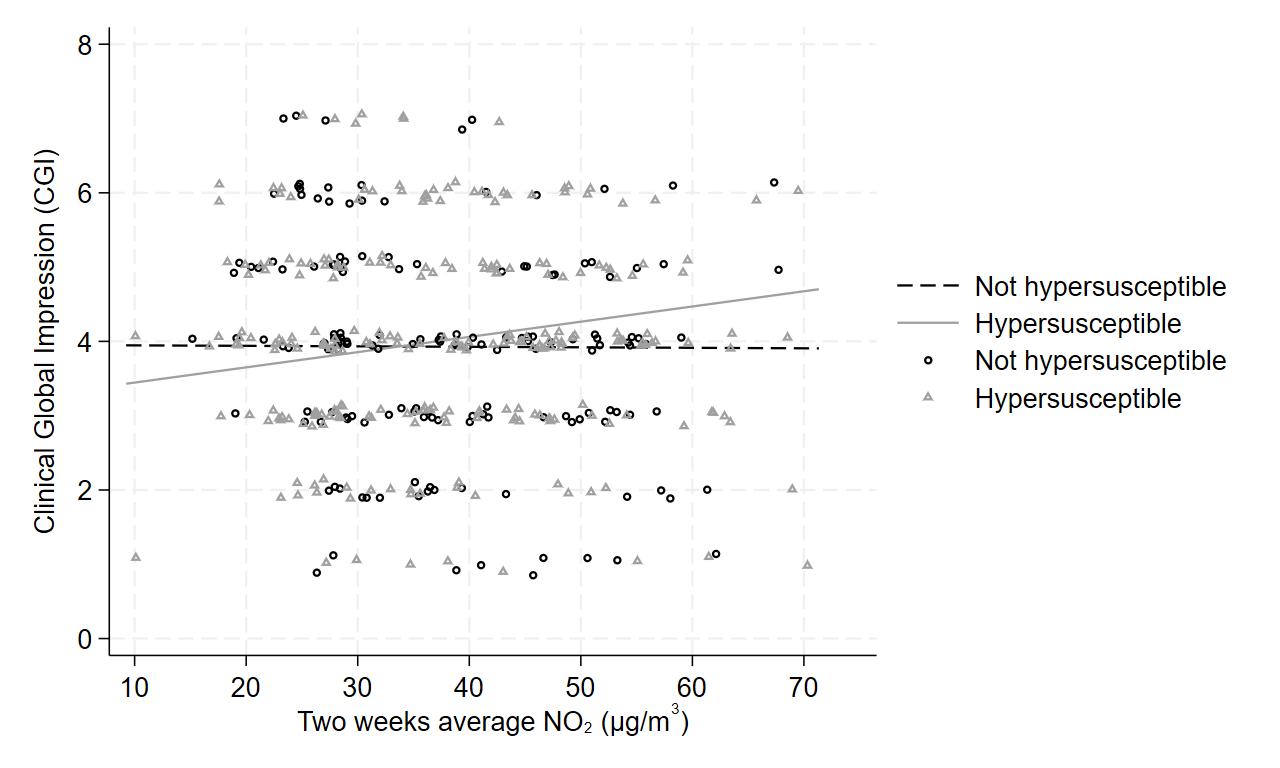 |

| **Supplementary Figure 22a** |
| --- |
| 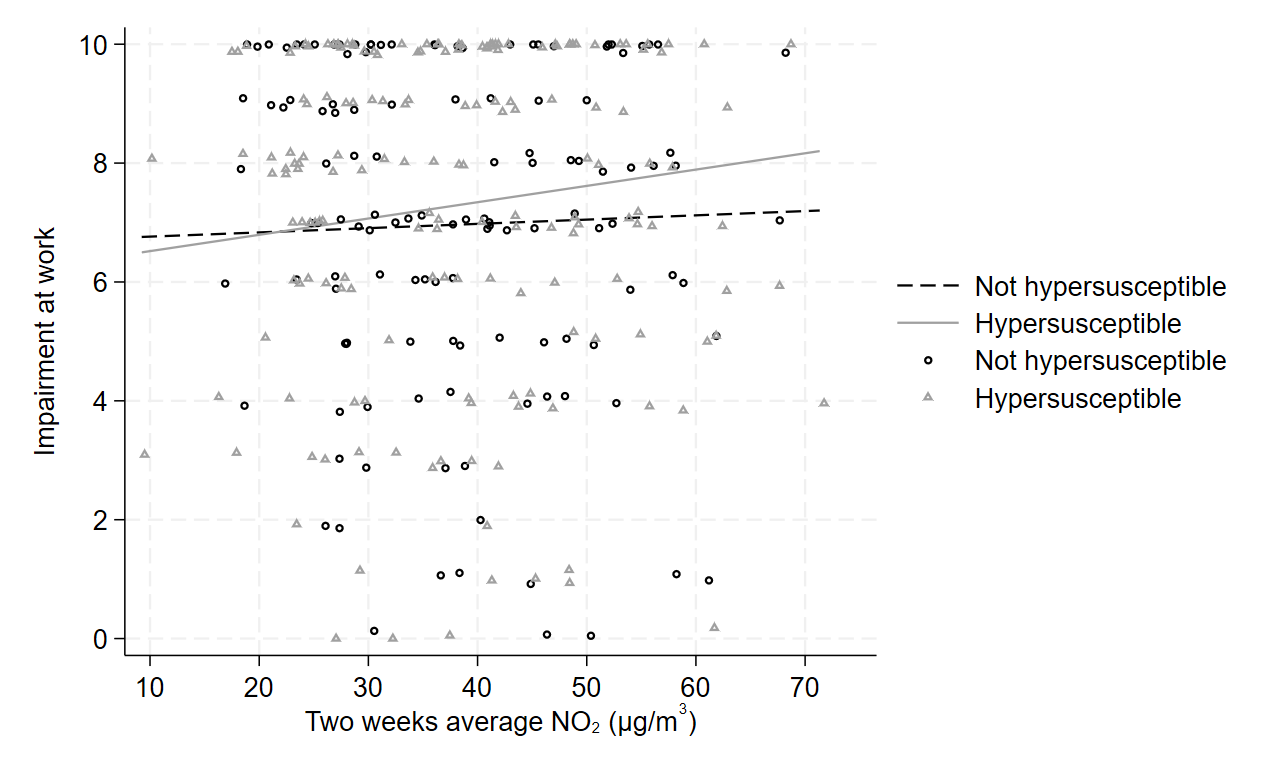 |
|  |
| **Supplementary Figure 22b** |
| 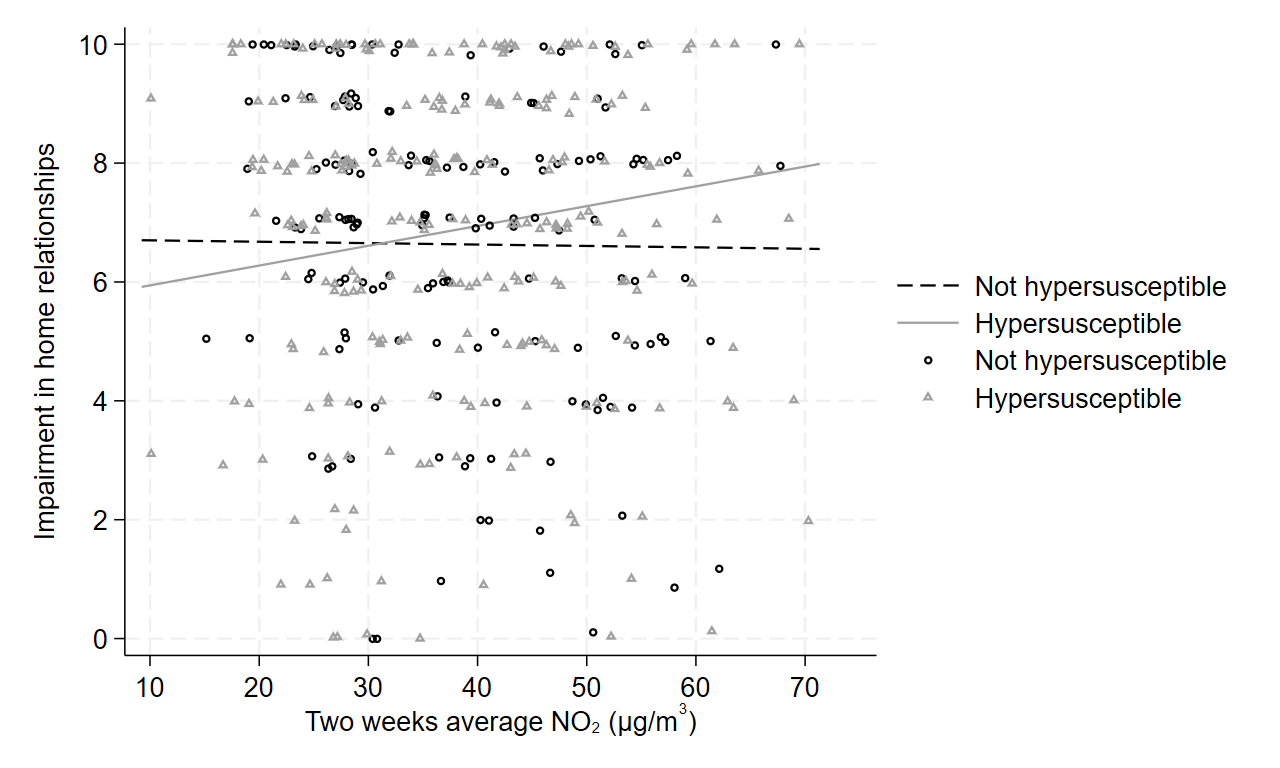 |

| **Supplementary Figure 22c** |
| --- |
| 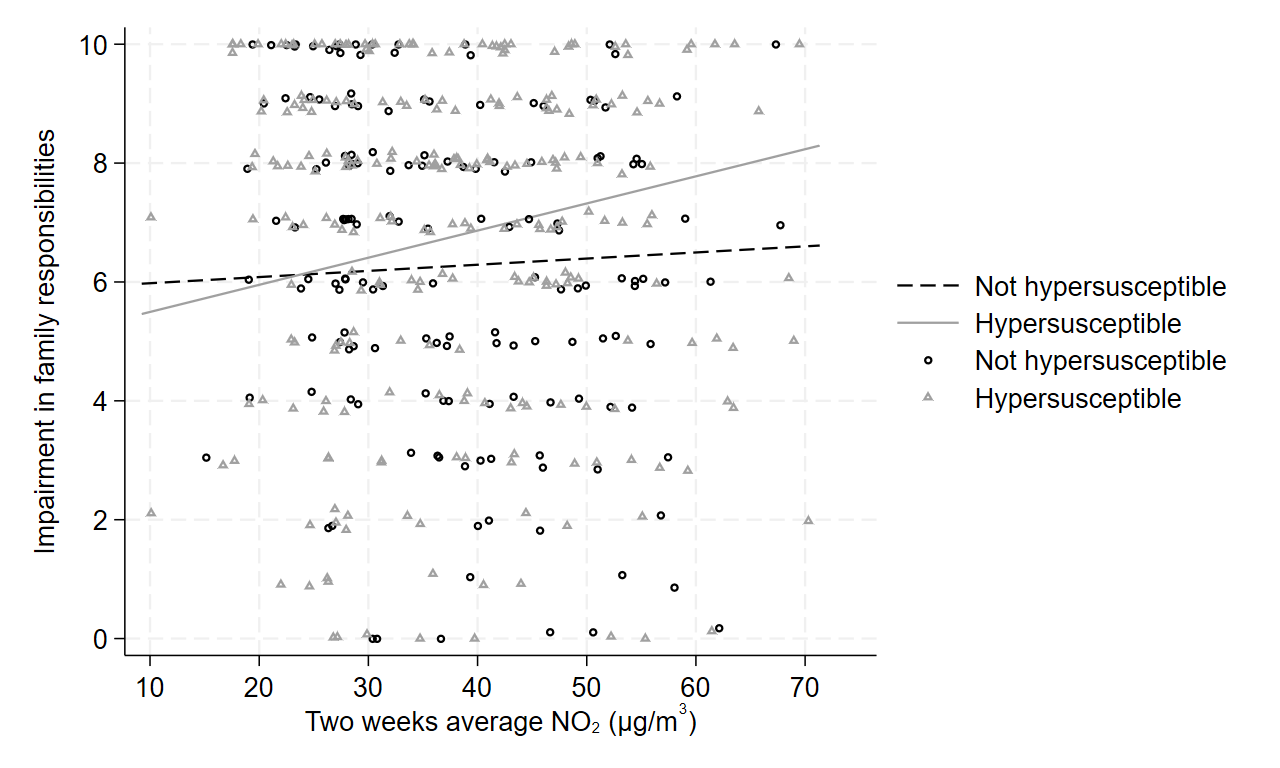 |
|  |
| **Supplementary Figure 22d** |
| 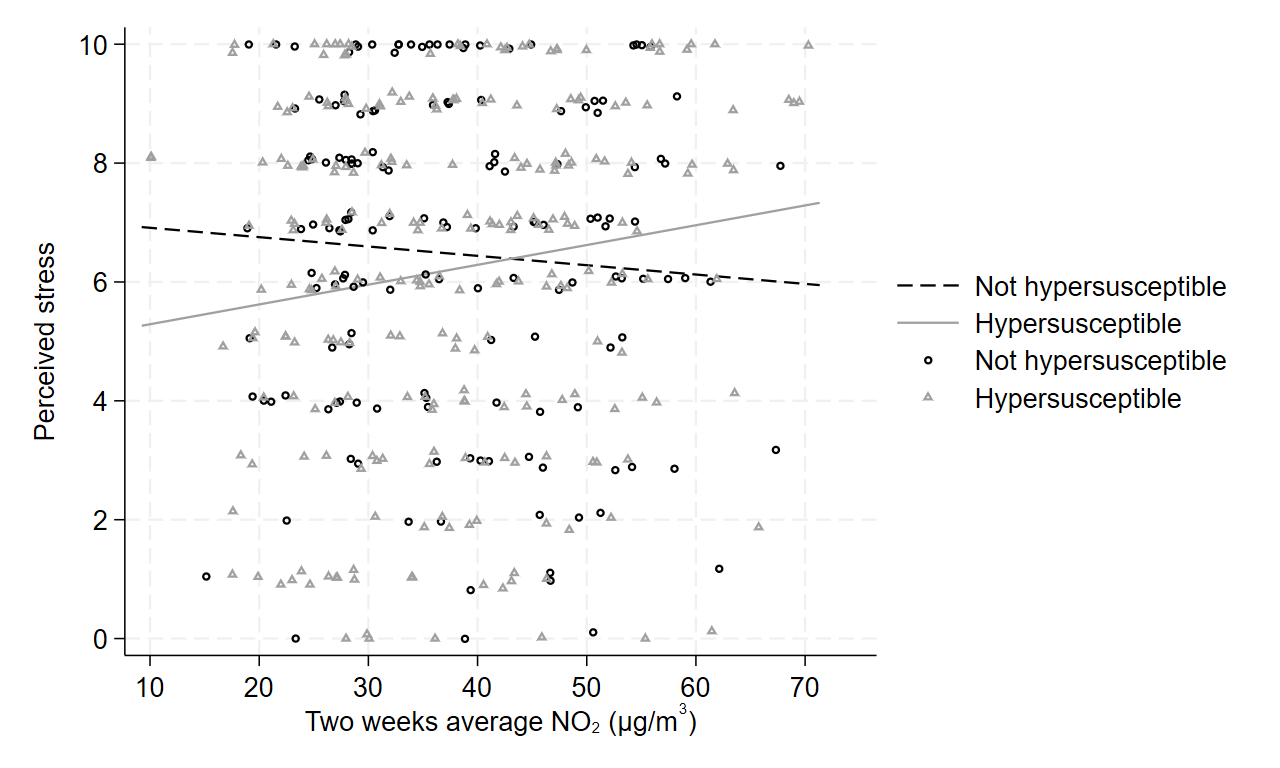 |

| **Supplementary Figure 22e** |
| --- |
| 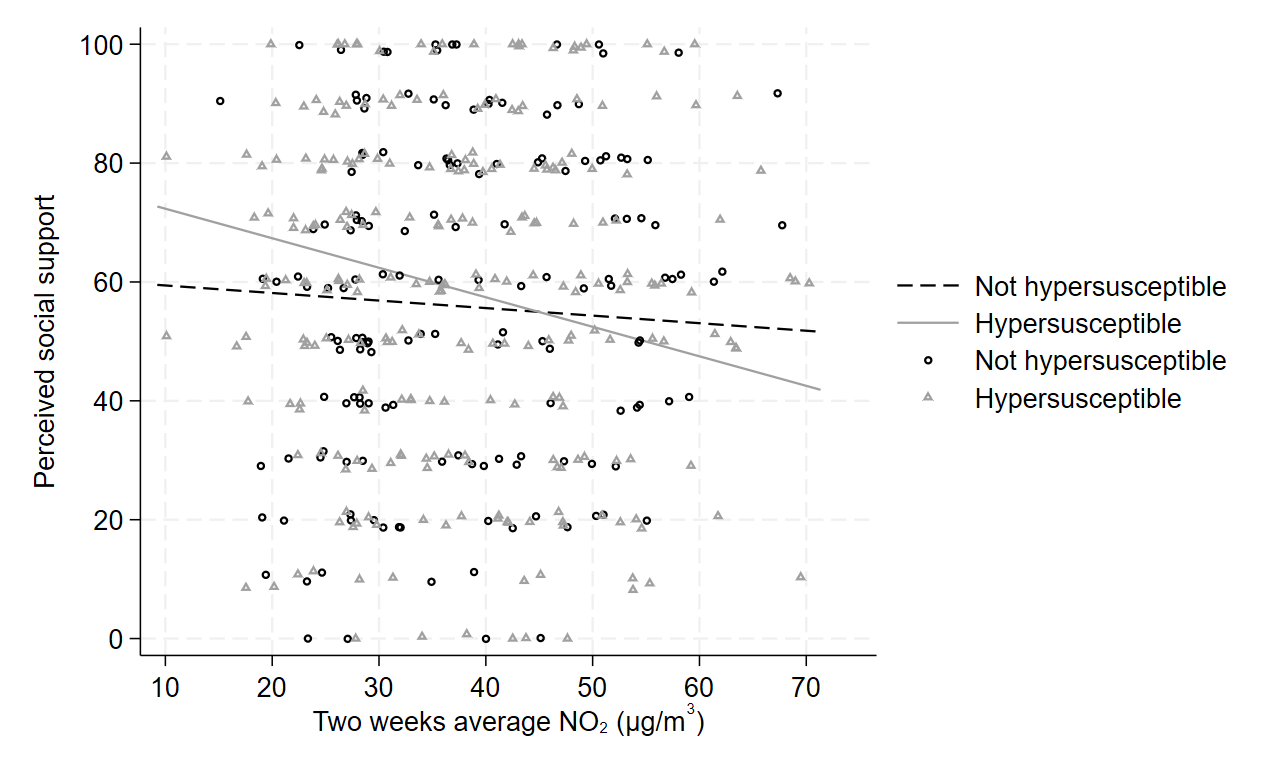 |

**Supplementary Figures 23-27**

Association between average PM2.5 exposure of the two weeks preceding recruitment and severity of depression, stratified by apparent temperature (AT, according to the rating scales MADRS (23), HAMD (24), GAF (25), CGI (26), and the five domains of the SDS (Impairment at work: 27a, Impairment in home relationships; 27b, Impairment in family responsibilities: 27c, Perceived stress: 27d, Perceived social support: 27e).

| **Supplementary Figure 23** |
| --- |
| 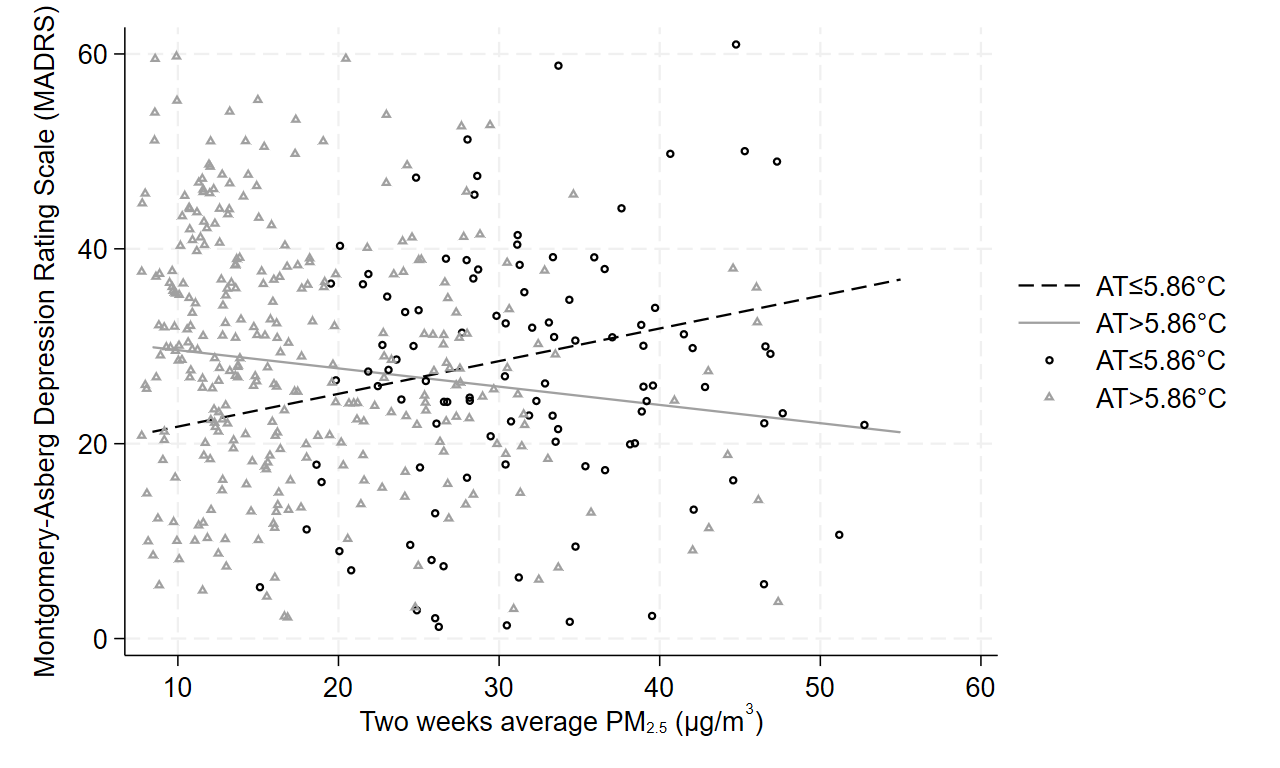 |
|  |
| **Supplementary Figure 24** |
| 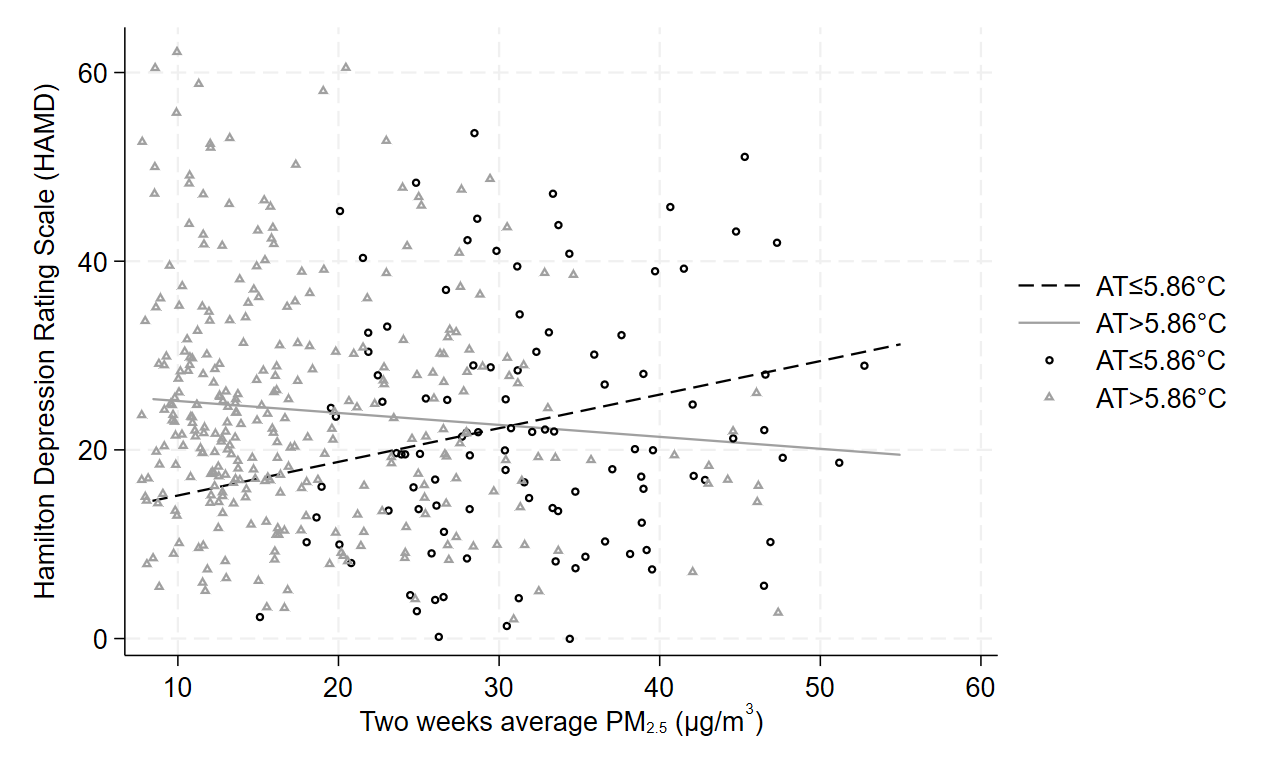 |

| **Supplementary Figure 25** |
| --- |
| 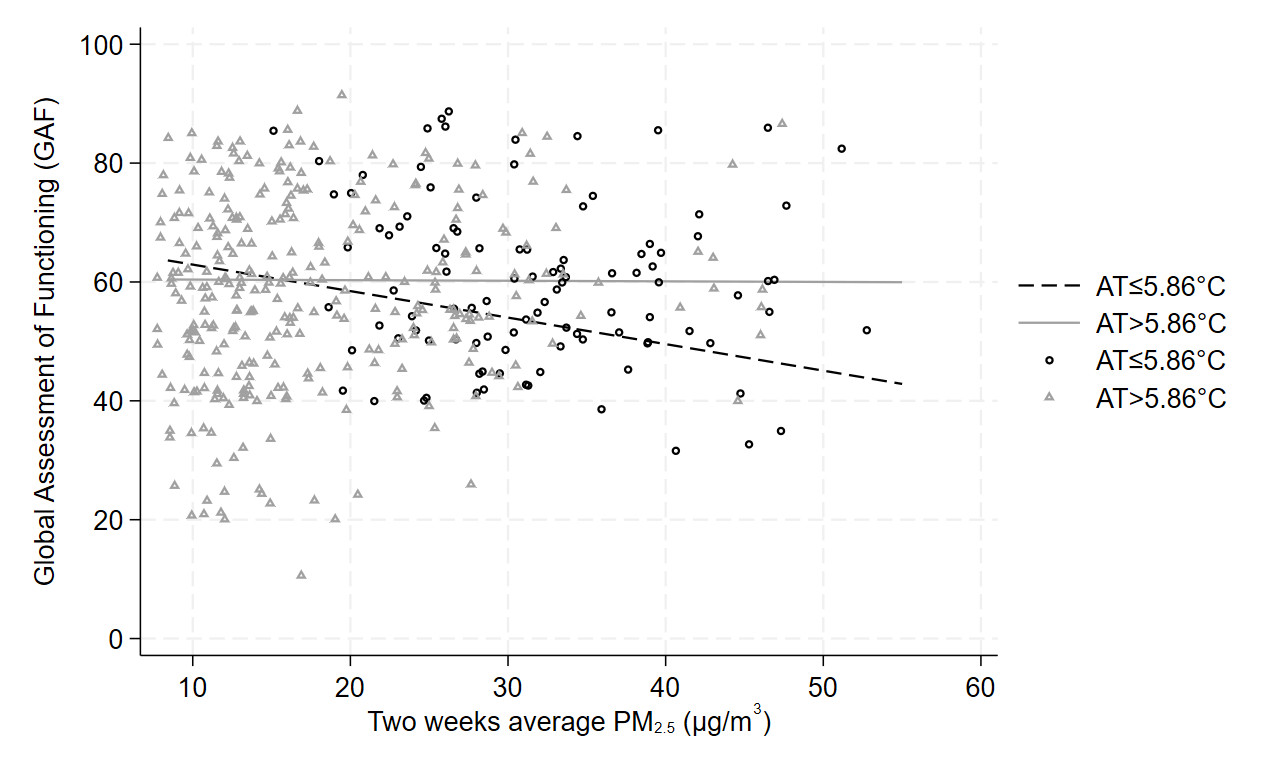 |
|  |
| **Supplementary Figure 26** |
| 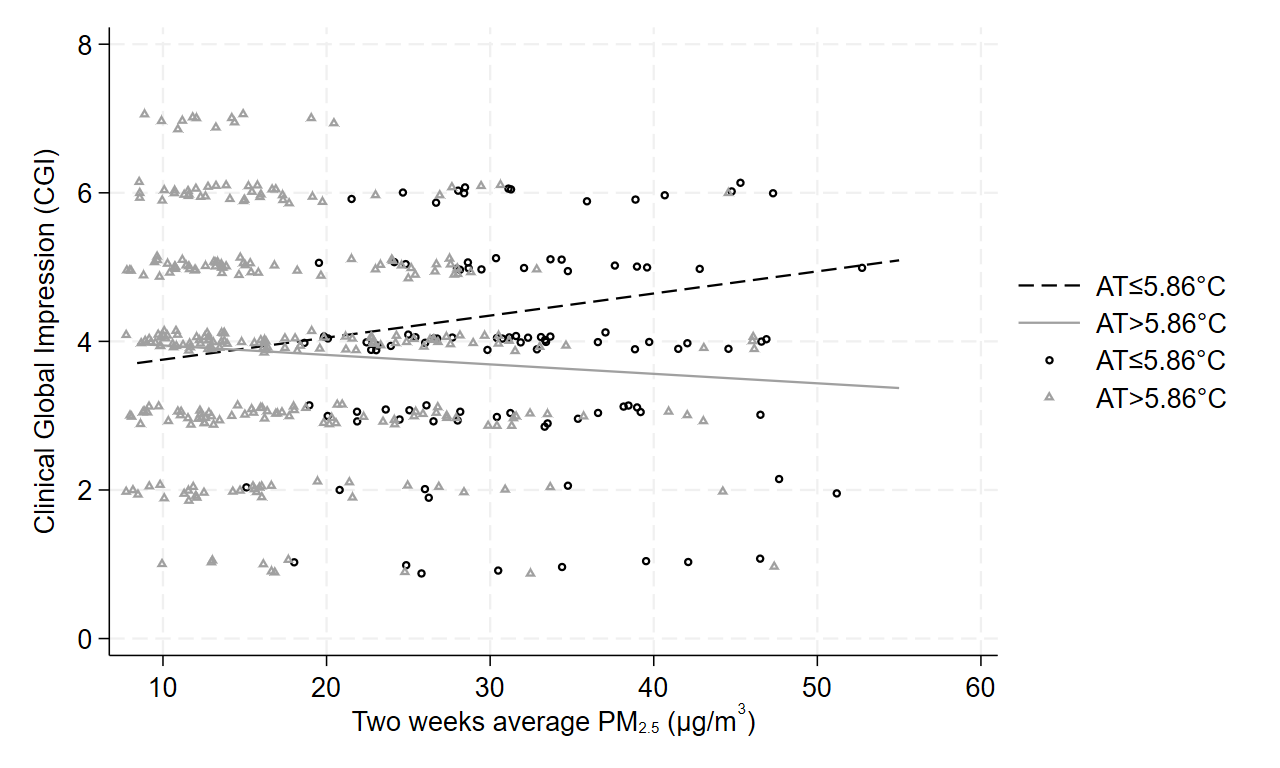 |

| **Supplementary Figure 27a** |
| --- |
| 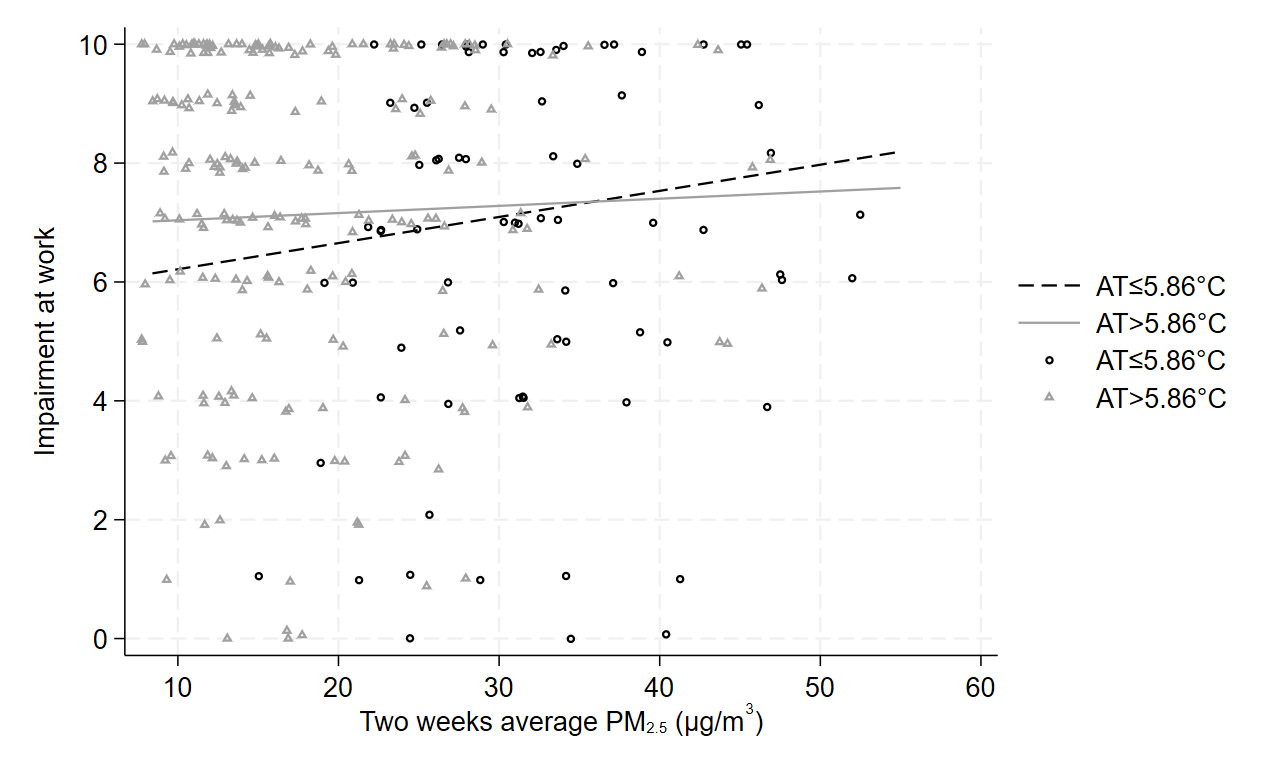 |
|  |
| **Supplementary Figure 27b** |
| 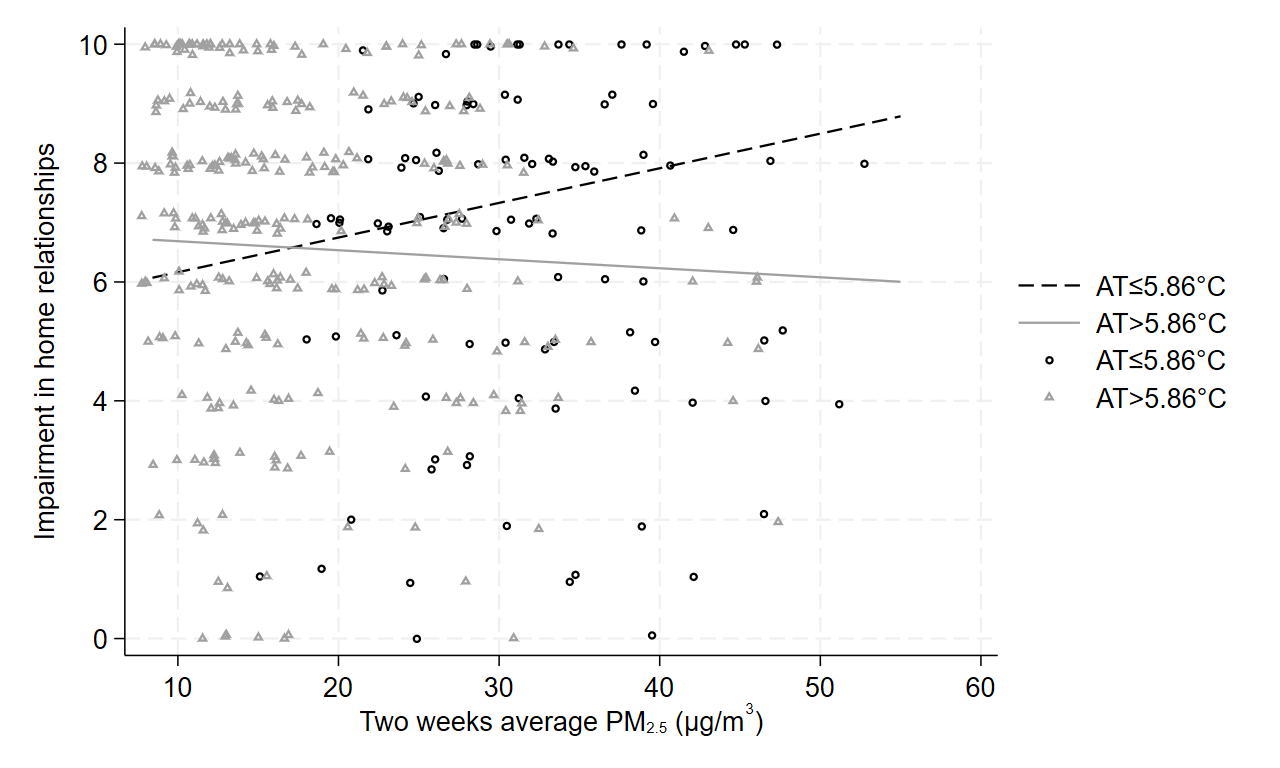 |

| **Supplementary Figure 27c** |
| --- |
| 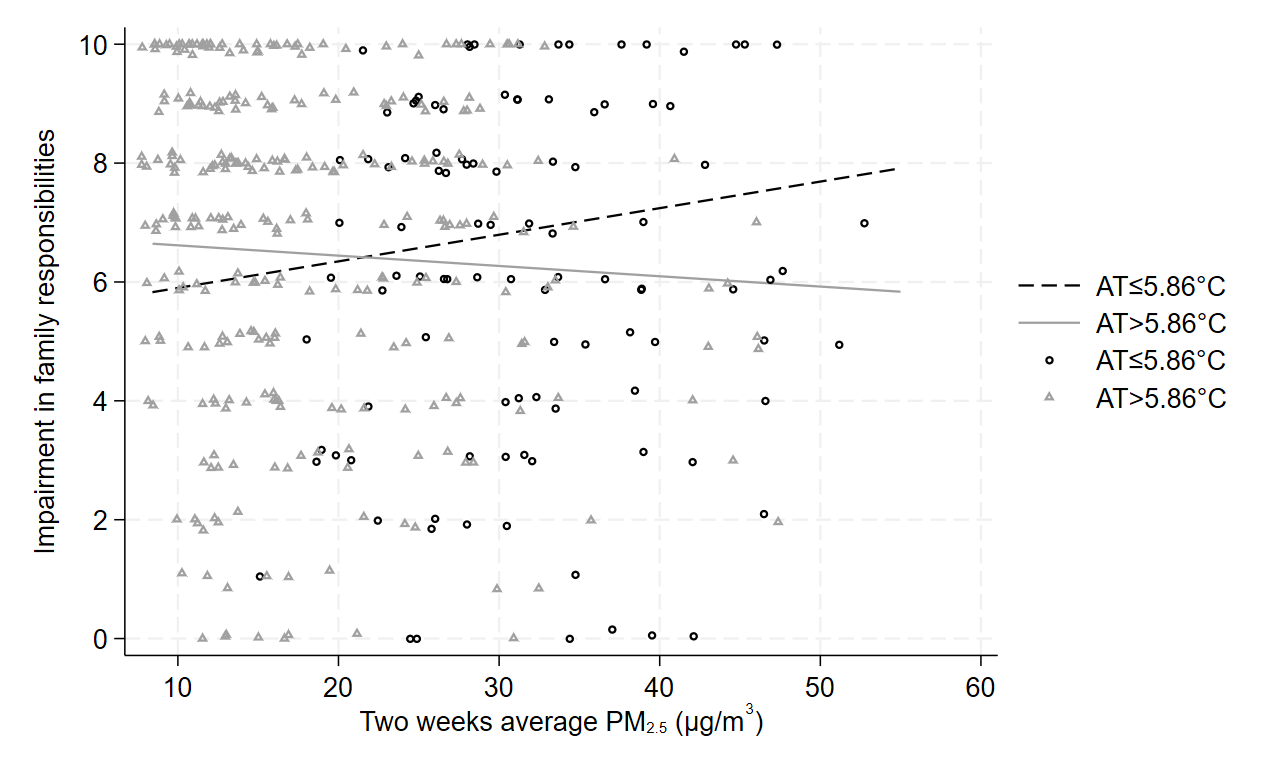 |
|  |
| **Supplementary Figure 27d** |
| 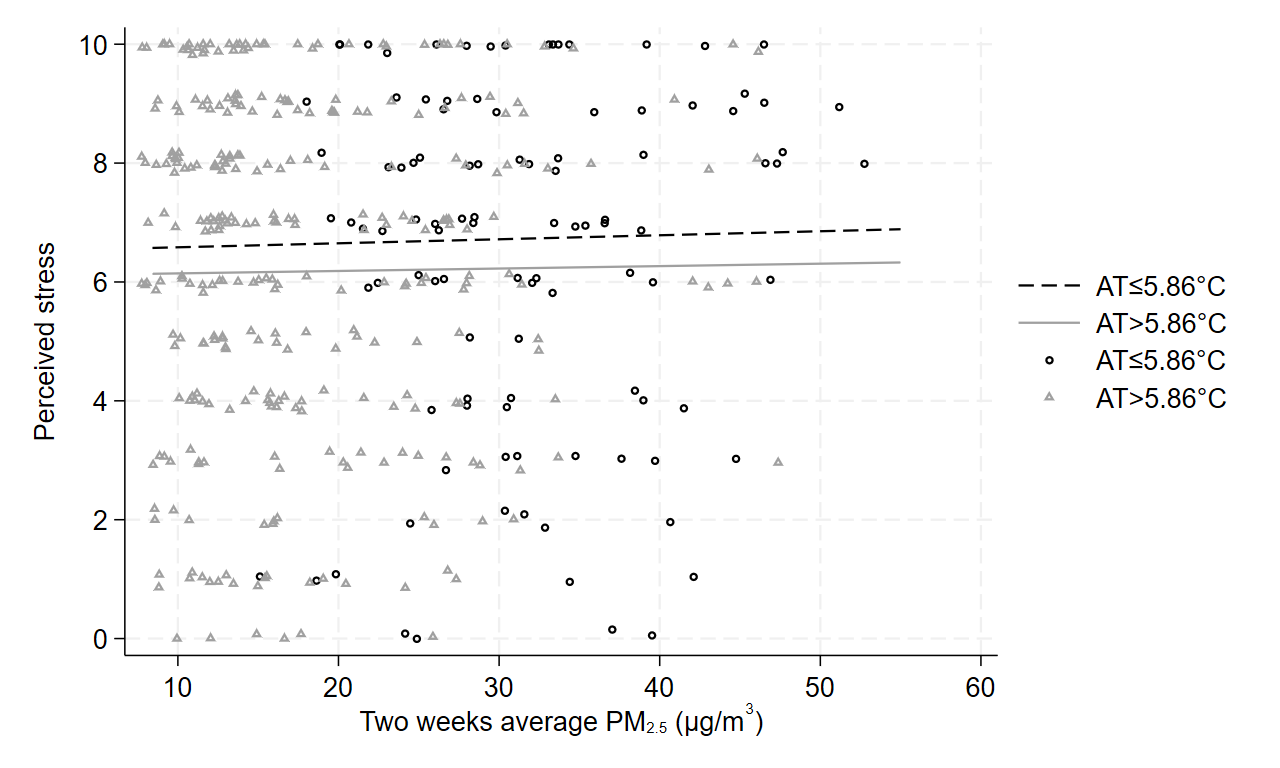 |

| **Supplementary Figure 27e** |
| --- |
| 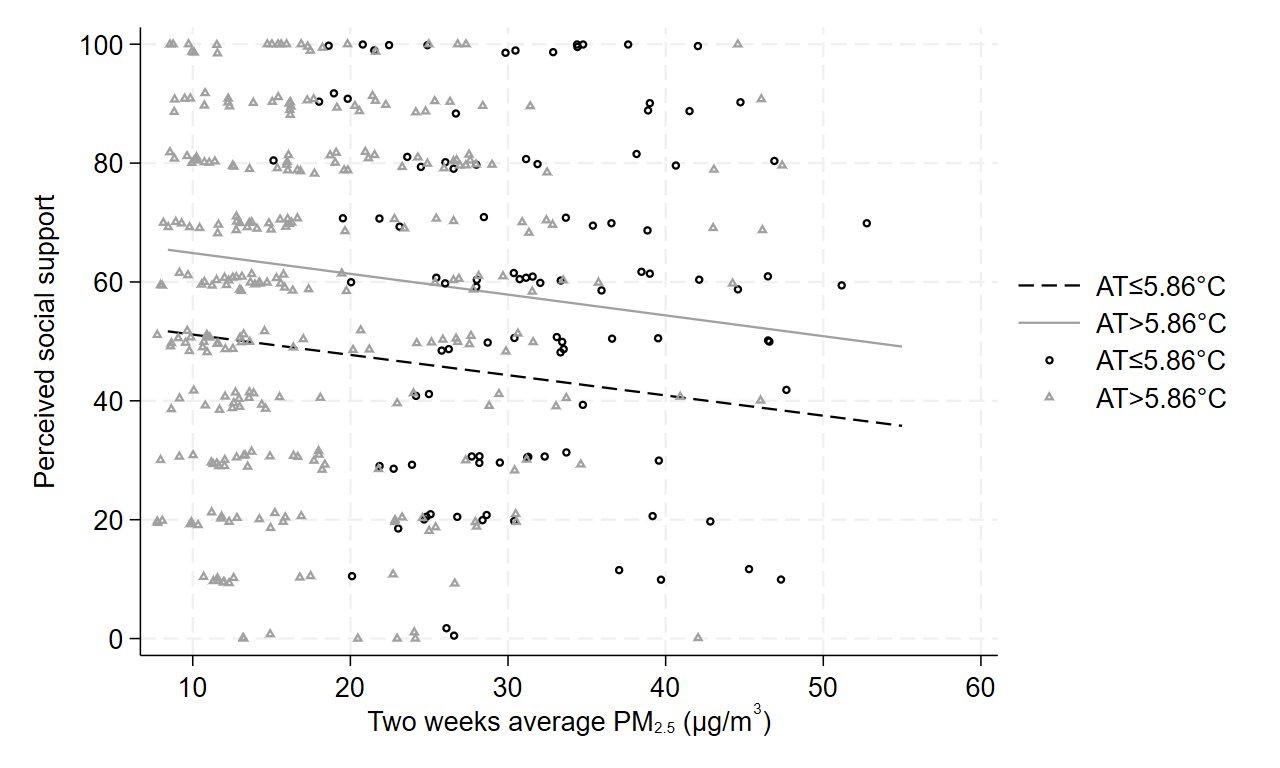 |

**Supplementary Figures 28-32**

Association between average NO_2_ exposure of the two weeks preceding recruitment and severity of depression, stratified by apparent temperature (AT), according to the rating scales MADRS (28), HAMD (29), GAF (30), CGI (31), and the five domains of the SDS (Impairment at work: 32a, Impairment in home relationships; 32b, Impairment in family responsibilities: 32c, Perceived stress: 32d, Perceived social support: 32e).

| **Supplementary Figure 28** |
| --- |
| 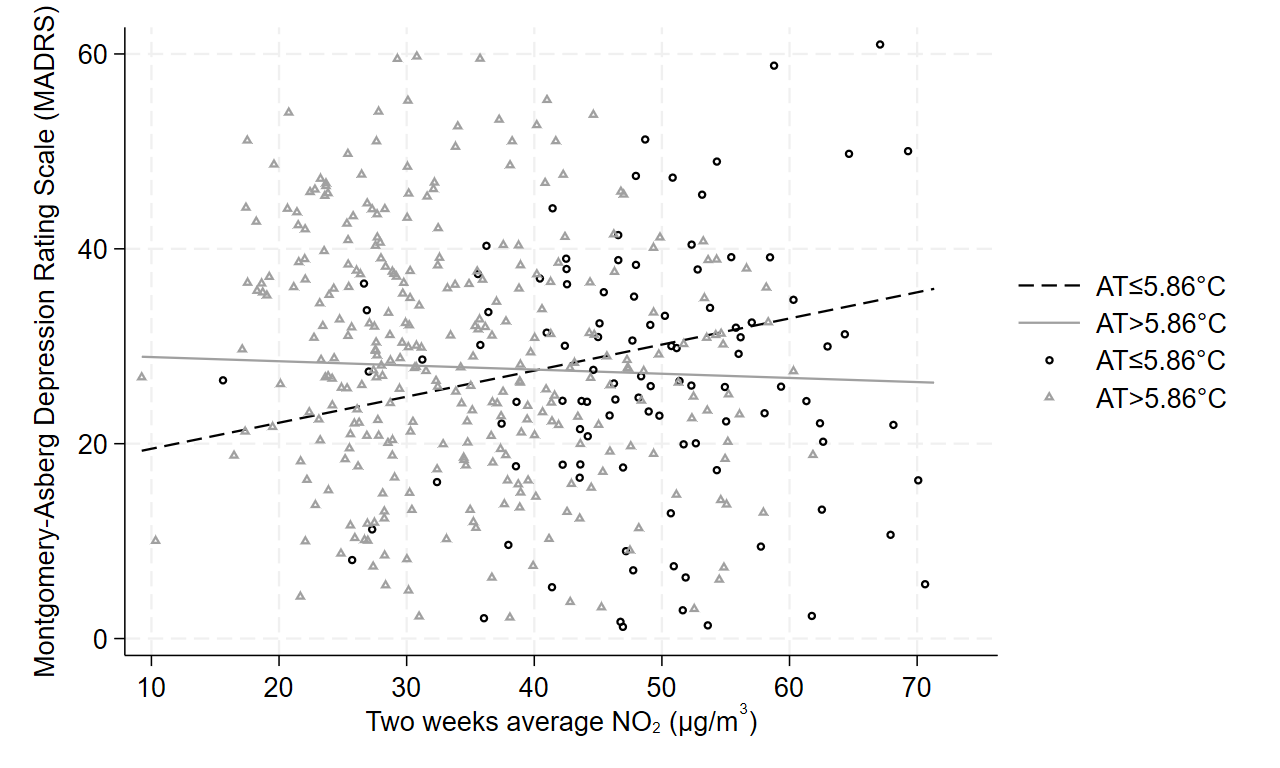 |
|  |
| **Supplementary Figure 29** |
| 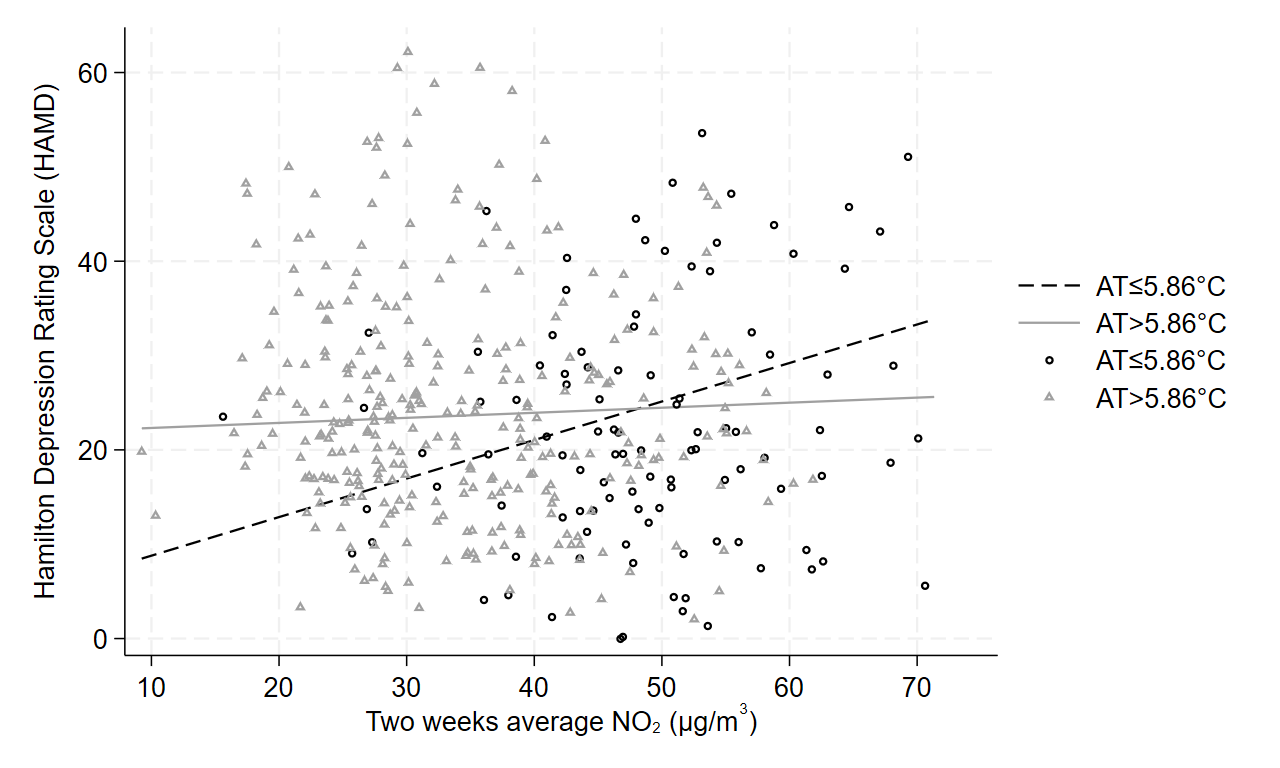 |

| **Supplementary Figure 30** |
| --- |
| 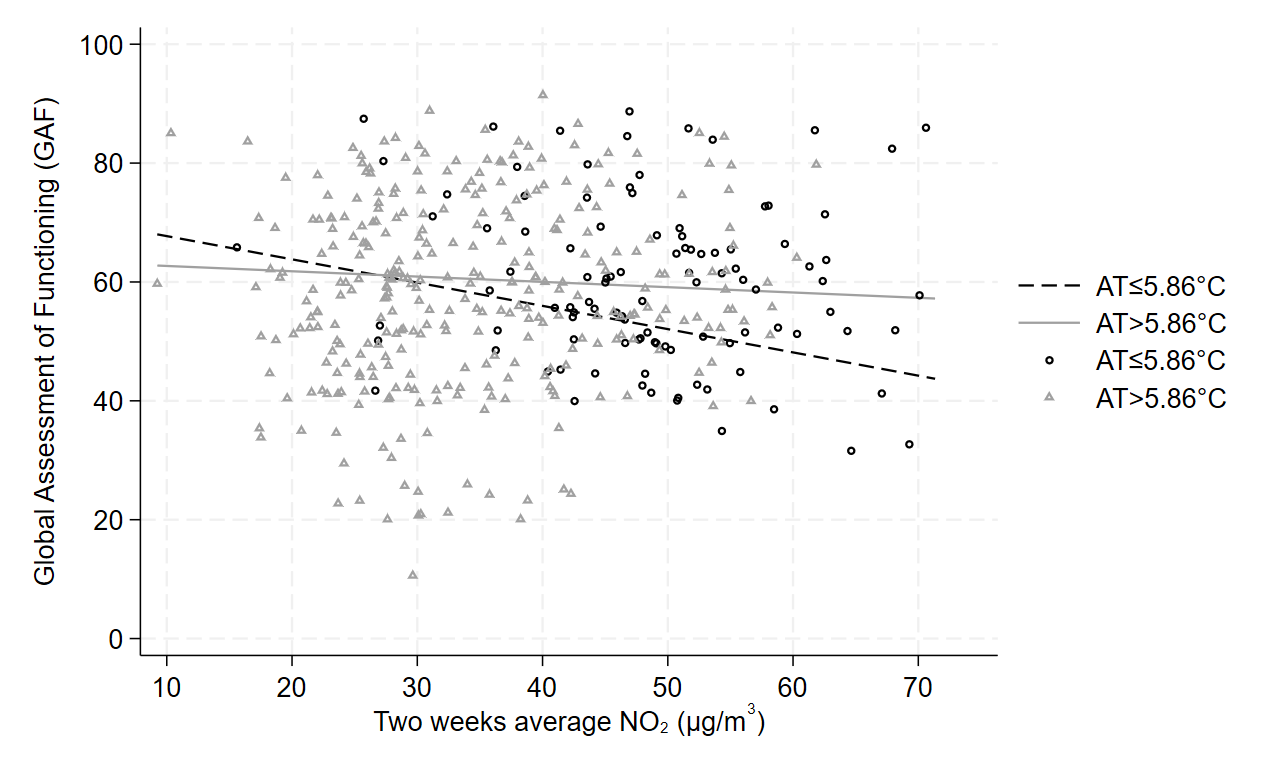 |
|  |
| **Supplementary Figure 31** |
| 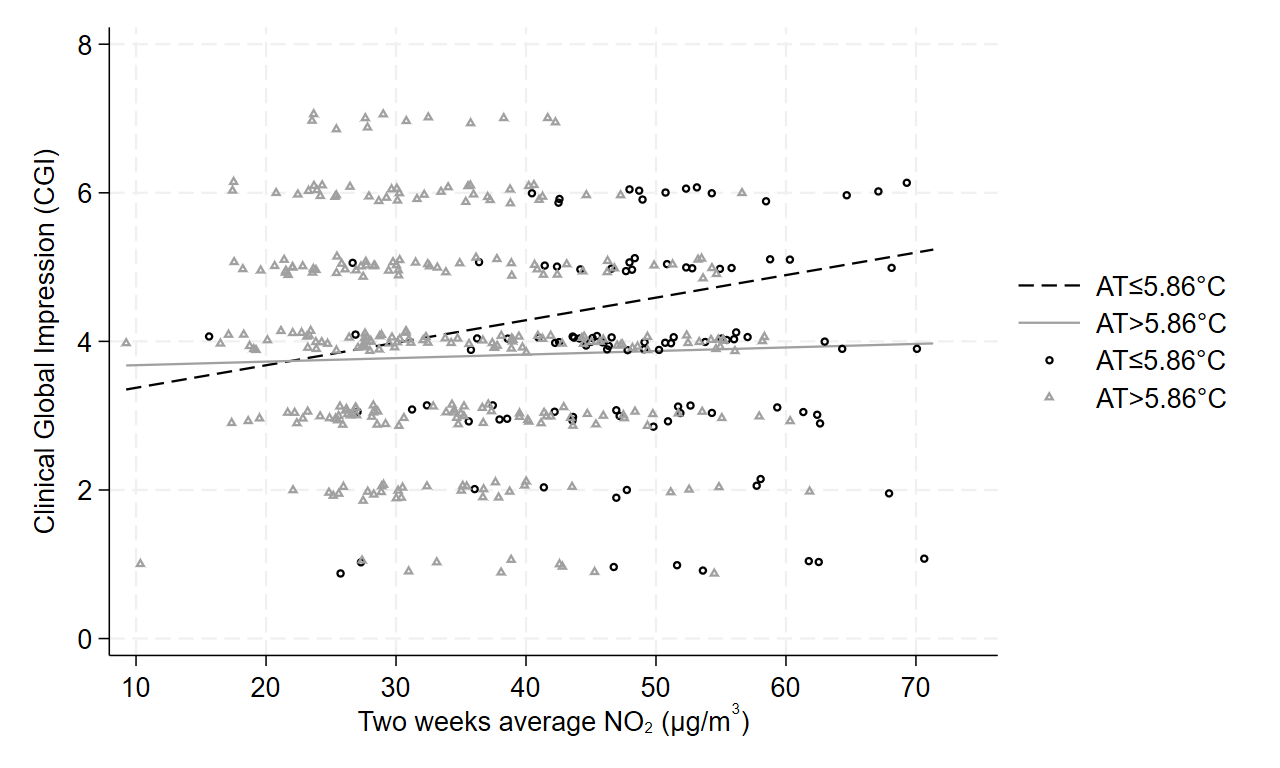 |

| **Supplementary Figure 32a** |
| --- |
| 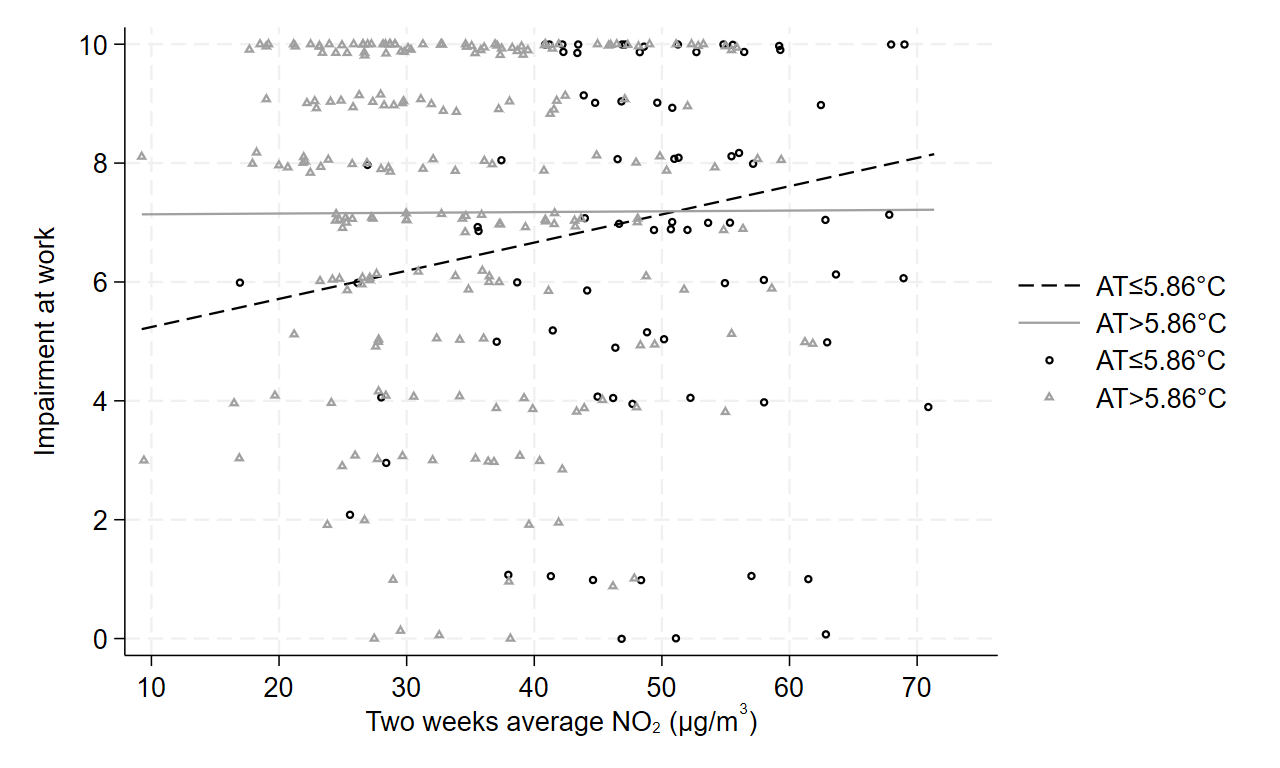 |
|  |
| **Supplementary Figure 32b** |
| 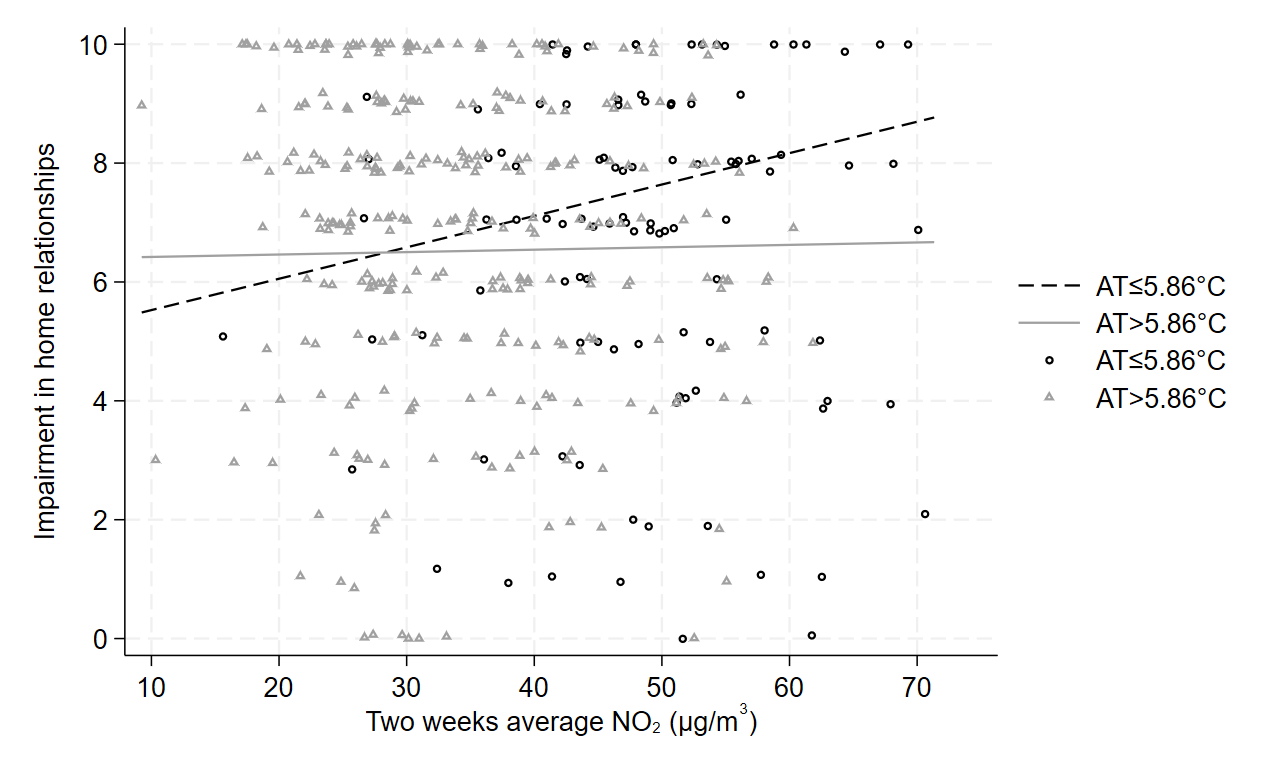 |

| **Supplementary Figure 32c** |
| --- |
| 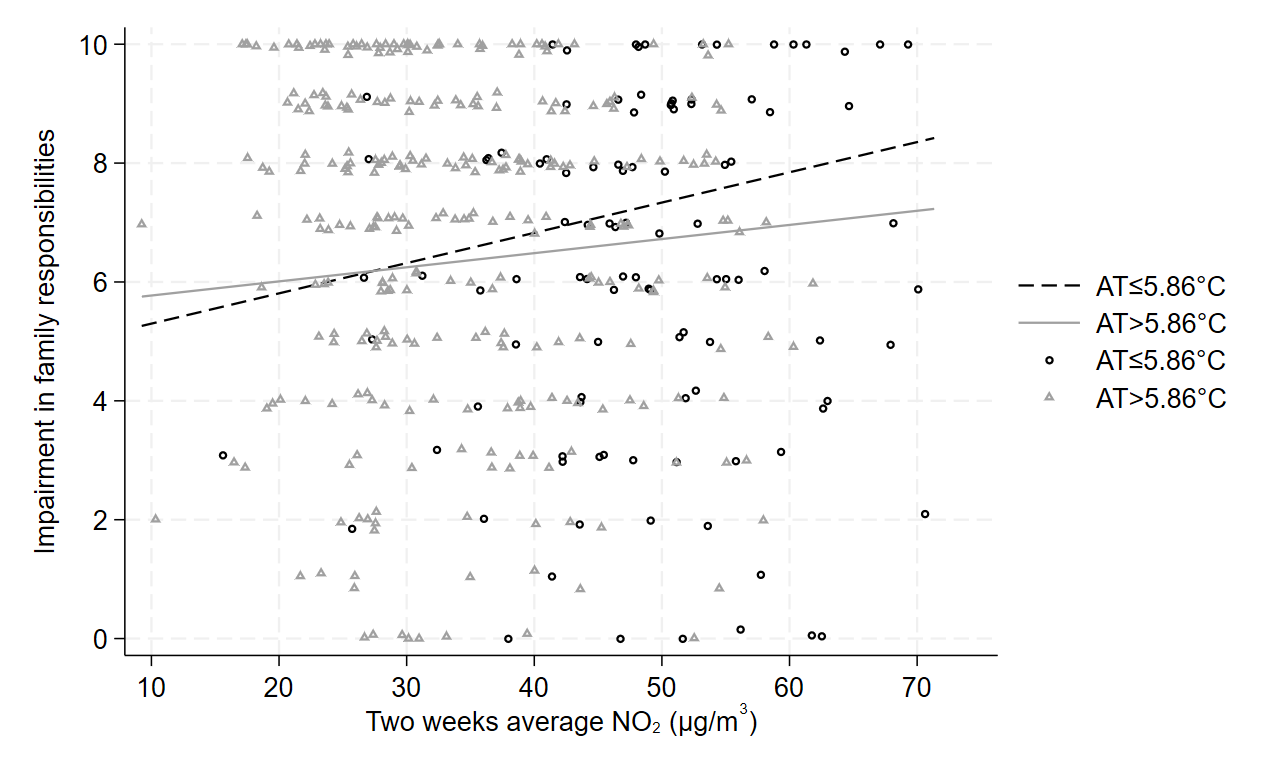 |
|  |
| **Supplementary Figure 32d** |
| 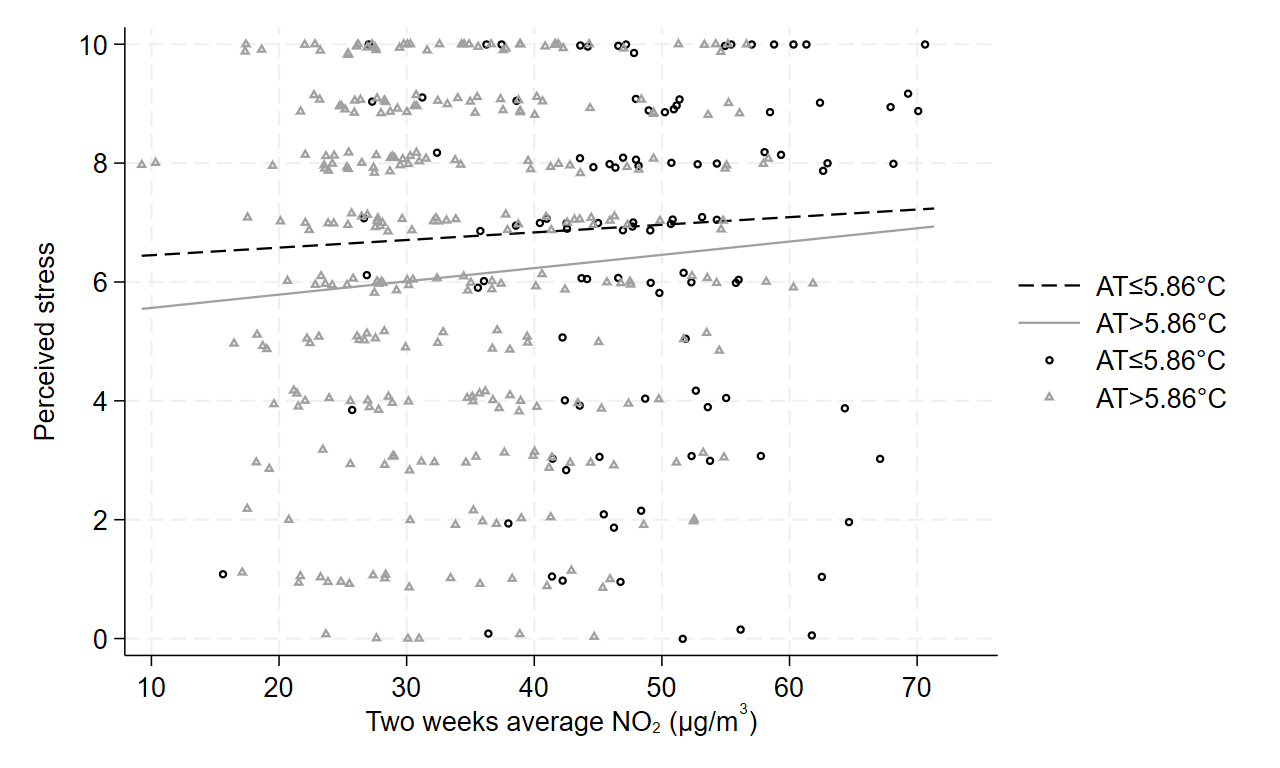 |

| **Supplementary Figure 32e** |
| --- |
| 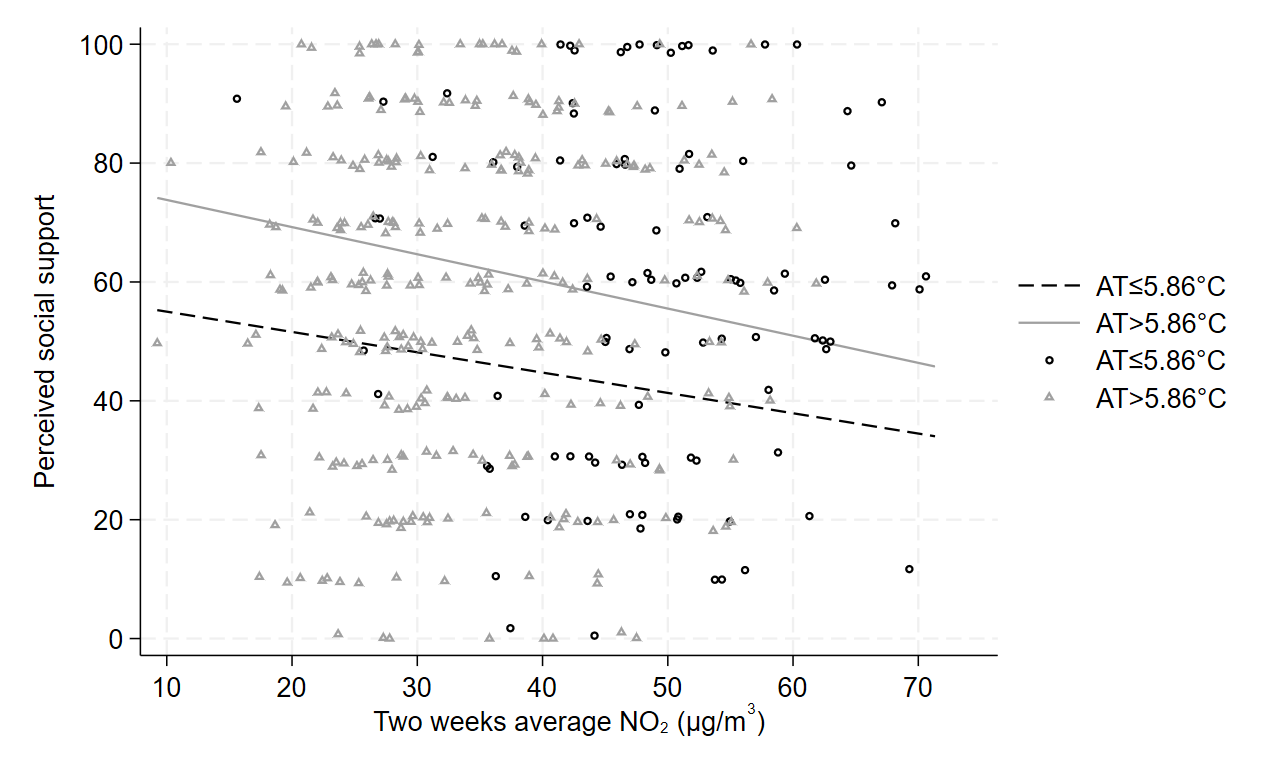 |

**Supplementary Figure 33**

Correlation between PM2.5 (left box) and NO2 (right box) FARM model estimates (X axis) vs. measurements from air quality monitoring stations (Y axis) at lag0 (i.e., daily average of the day recruitment).


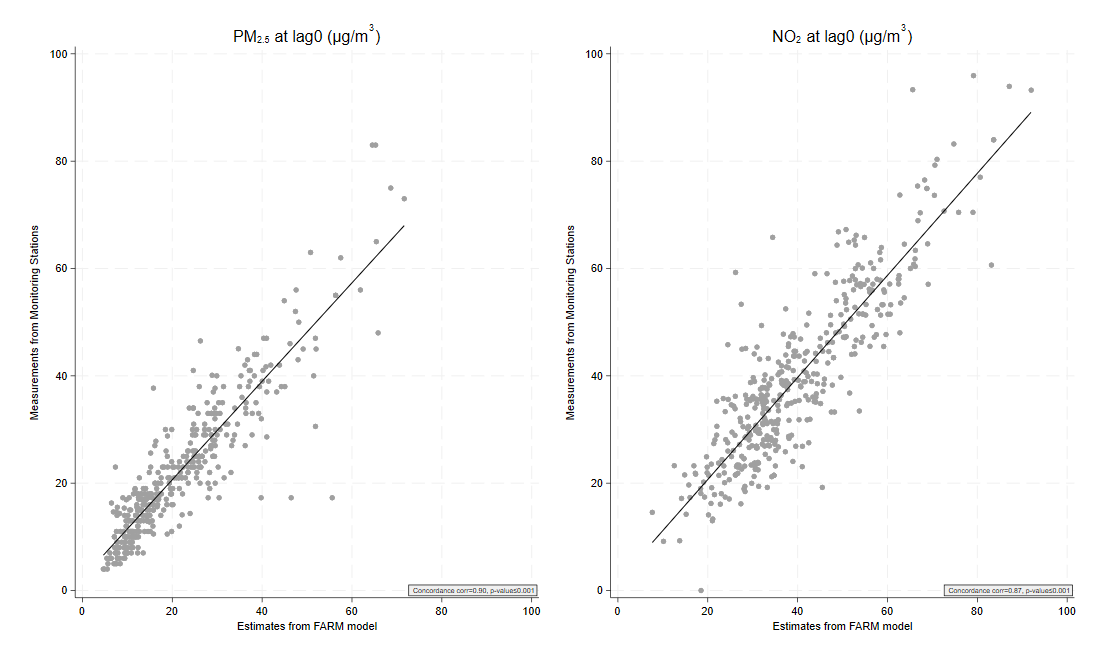

Supplement: Borroni et al. supplementary material 1 — Borroni et al. supplementary material [file S092493382401767Xsup001.docx]
