# Supplementary material for: Impact of air pollution exposure on the severity of major depressive disorder: Results from the DeprAir study
Source: Eur Psychiatry. 2024 Sep 27;67(1):e61. doi: 10.1192/j.eurpsy.2024.1767 (PMC11457114; doi:10.1192/j.eurpsy.2024.1767)
Supplement: Borroni et al. supplementary material 2 — Borroni et al. supplementary material [file S092493382401767Xsup002.docx]

**Supplementary Table 1:** Estimates (β) with corresponding 95% confidence intervals (95%CI), and p-values of the association between PM10 exposure (10 µg/m^3^ increase) and Major Depressive Disorder severity rating scales.

| **MDD Rating scale** | **β (95%CI) p-value** |
| --- | --- |
| MADRS | 0.49 (-0.93; 1.91) p=0.495 |
| HAMD | 0.75 (-0.63; 2.14) p=0.287 |
| GAF | -1.51 (-3.28; 0.26) p=0.094 |
| CGI | 0.07 (-0.15; 0.28) p=0.546 |
| SDS |  |
| Impairment at work | 0.20 (-0.18; 0.59) p=0.301 |
| Impairment in home relationships | 0.10 (-0.21; 0.41) p=0.513 |
| Impairment in family responsibilities | 0.04 (-0.29; 0.37) p=0.823 |
| Perceived stress | 0.10 (-0.24; 0.44) p=0.550 |
| Perceived social support | -3.08 (-6.31; 0.15) p=0.061 |

**Supplementary Table 2**: Estimates with corresponding confidence intervals and p-values of the association between air pollutant exposure (10 µg/m^3^ increase) assessed through air quality monitoring station data and Major Depressive Disorder severity rating scales.

| **MDD Rating scale** | **β (95%CI) p-value** | |
| --- | --- | --- |
|  | **PM2.5** | **NO_2_** |
| MADRS | -0.42 (-2.20; 1.37) p=0.644 | **1.43 (0.02; 2.84) p=0.046** |
| HAMD | 0.27 (-1.47; 2.02) p=0.759 | **1.94 (0.56; 3.31) p=0.006** |
| GAF | -0.34 (-2.57; 1.89) p=0.765 | **-1.76 (-3.52; 0.00) p=0.050** |
| CGI | -0.03 (-0.30; 0.24) p=0.822 | 0.18 (-0.03; 0.39) p=0.105 |
| SDS |  |  |
| Impairment at work | 0.17 (-0.31; 0.65) p=0.497 | **0.44 (0.06; 0.81) p=0.024** |
| Impairment in home relationships | -0.08 (-0.47; 0.31) p=0.680 | 0.30 (-0.00; 0.61) p=0.052 |
| Impairment in family responsibilities | -0.08 (-0.49; 0.33) p=0.702 | 0.32 (-0.00; 0.65) p=0.054 |
| Perceived stress | 0.18 (-0.25; 0.60) p=0.416 | 0.23 (-0.11; 0.56) p=0.191 |
| Perceived social support | -1.14 (-5.21; 2.93) p=0.582 | **-3.92 (-7.13; -0.71) p=0.017** |

Legend: MDD: Major Depressive Disorder; MADRS: Montgomery Asberg Depression Rating Scale; HAMD: Hamilton Depression Rating Scale; GAF: Global Assessment of Functioning; CGI: Clinical Global Impression; SDS: Sheehan Disability Scale; PM2.5: particulate matter with diameter less than or equal to 2.5; NO_2_: nitrogen dioxide; β: beta estimate; 95%CI: confidence interval at 95% level

**Supplementary Table 3**: Stratified estimates (β) by hypersusceptibility status (defined as presence of at least one of the following: obesity, hypercholesterolemia, hypertension, type II diabetes, current smoking), with corresponding 95% confidence intervals (95%CI), p-values, and interaction p-values of the association between PM10 exposure (10 µg/m^3^ increase) and Major Depressive Disorder severity rating scales.

| **MDD Rating scale** | **β (95%CI) p-value** | | ***Interaction p-value*** |
| --- | --- | --- | --- |
|  | **Hypersusceptible subjects** | **Not Hypersusceptible subjects** |  |
| MADRS | 1.24 (-0.32; 2.79) p=0.118 | -0.91 (-2.76; 0.95) p=0.337 | ***0.023*** |
| HAMD | 1.30 (-0.22; 2.83) p=0.094 | -0.24 (-2.06; 1.58) p=0.795 | *0.095* |
| GAF | -2.00 (-3.94; -0.05) p=0.044 | -0.63 (-2.95; 1.69) p=0.594 | *0.246* |
| CGI | 0.16 (-0.08; 0.40) p=0.190 | -0.12 (-0.41; 0.17) p=0.425 | *0.063* |
| SDS |  |  |  |
| Impairment at work | 0.29 (-0.13; 0.71) p=0.180 | 0.07 (-0.42; 0.55) p=0.782 | *0.362* |
| Impairment in home relationships | 0.25 (-0.08; 0.59) p=0.141 | -0.17 (-0.57; 0.23) p=0.411 | ***0.039*** |
| Impairment in family responsibilities | 0.18 (-0.18; 0.54) p=0.319 | -0.22 (-0.65; 0.21) p=0.322 | *0.067* |
| Perceived stress | 0.31 (-0.06; 0.68) p=0.101 | -0.29 (-0.73; 0.15) p=0.201 | ***0.008*** |
| Perceived social support | **-3.73 (-7.28; -0.18) p=0.040** | -1.80 (-6.03; 2.44) p=0.404 | *0.370* |

Legend: MDD: Major Depressive Disorder; MADRS: Montgomery Asberg Depression Rating Scale; HAMD: Hamilton Depression Rating Scale; GAF: Global Assessment of Functioning; CGI: Clinical Global Impression; SDS: Sheehan Disability Scale; PM_10_: particulate matter with diameter less than or equal to 10; β: beta estimate; 95% CI: confidence interval at 95% level

**Supplementary Table 4**: Stratified estimates (β) by apparent temperature (AT), with corresponding 95% confidence intervals (95%CI), p-values, and interaction p-values of the association between PM10 exposure (10 µg/m^3^ increase) and Major Depressive Disorder severity rating scales.

| **MDD Rating scale** | **β (95%CI) p-value** | | ***Interaction p-value*** |
| --- | --- | --- | --- |
|  | **AT ≤ 1^st^ quartile (5.86 °C)** | **AT > 1^st^ quartile (5.86 °C)** |  |
| MADRS | **2.54 (0.11; 4.98) p=0.041** | -0.89 (-2.59; 0.81) p=0.303 | ***0.023*** |
| HAMD | **3.21 (0.83; 5.58) p=0.008** | -0.89 (-2.54; 0.77) p=0.292 | ***0.005*** |
| GAF | **-3.27 (-6.30; -0.24) p=0.034** | -0.34 (-2.45; 1.77) p=0.753 | *0.117* |
| CGI | 0.30 (-0.09; 0.68) p=0.129 | -0.04 (-0.29; 0.22) p=0.771 | *0.155* |
| SDS |  |  |  |
| Impairment at work | 0.38 (-0.29; 1.05) p=0.267 | -0.03 (-0.48; 0.42) p=0.900 | *0.314* |
| Impairment in home relationships | 0.39 (-0.13; 0.92) p=0.144 | -0.09 (-0.46; 0.27) p=0.621 | *0.136* |
| Impairment in family responsibilities | 0.29 (-0.28; 0.86) p=0.318 | -0.16 (-0.56; 0.23) p=0.417 | *0.197* |
| Perceived stress | -0.04 (-0.62; 0.55) p=0.895 | 0.23 (-0.18; 0.64) p=0.271 | *0.458* |
| Perceived social support | -1.84 (-7.35; 3.67) p=0.511 | -3.96 (-7.79; -0.12) p=0.043 | *0.533* |

Legend: MDD: Major Depressive Disorder; MADRS: Montgomery Asberg Depression Rating Scale; HAMD: Hamilton Depression Rating Scale; GAF: Global Assessment of Functioning; CGI: Clinical Global Impression; SDS: Sheehan Disability Scale; PM_10_: particulate matter with diameter less than or equal to 10; β: beta estimate; 95% CI: confidence interval at 95% level
